# Input-Specific Gain Modulation by Local Sensory Context Shapes Cortical and Thalamic Responses to Complex Sounds

**DOI:** 10.1016/j.neuron.2016.05.041

**Published:** 2016-07-20

**Authors:** Ross S. Williamson, Misha B. Ahrens, Jennifer F. Linden, Maneesh Sahani

**Affiliations:** 1 Gatsby Computational Neuroscience Unit, University College London, London W1T 4JG, UK; 2 Centre for Mathematics and Physics in the Life Sciences and Experimental Biology, University College London, London WC1E 6BT, UK; 3 Department of Molecular and Cellular Biology, Harvard University, Cambridge, MA 02138, USA; 4 Computational and Biological Learning Lab, Department of Engineering, University of Cambridge, Cambridge CB2 1PZ, UK; 5 Ear Institute, University College London, London WC1X 8EE, UK; 6 Department of Neuroscience, Physiology and Pharmacology, University College London, London WC1E 6BT, UK

## Abstract

Sensory neurons are customarily characterized by one or more linearly weighted receptive fields describing sensitivity in sensory space and time. We show that in auditory cortical and thalamic neurons, the weight of each receptive field element depends on the pattern of sound falling within a local neighborhood surrounding it in time and frequency. Accounting for this change in effective receptive field with spectrotemporal context improves predictions of both cortical and thalamic responses to stationary complex sounds. Although context dependence varies among neurons and across brain areas, there are strong shared qualitative characteristics. In a spectrotemporally rich soundscape, sound elements modulate neuronal responsiveness more effectively when they coincide with sounds at other frequencies, and less effectively when they are preceded by sounds at similar frequencies. This local-context-driven lability in the representation of complex sounds—a modulation of “input-specific gain” rather than “output gain”—may be a widespread motif in sensory processing.

## Highlights

• Gain of neuronal responses to sound components varies with immediate acoustic context
• “Contextual gain fields” can be estimated from neuronal responses to complex sounds
• Coincident sound at different frequencies boosts gain in cortex and thalamus
• Preceding sound at similar frequency reduces gain for longer in cortex than thalamus

## Teaser

Williamson et al. (2016) show how encoding of individual components within complex sounds depends on the immediate acoustic neighborhood surrounding each component. These findings challenge the model that nonlinearity only follows integration, highlighting instead fine-grained nonlinear interactions within the receptive field.

## Introduction

**Figure 1:**
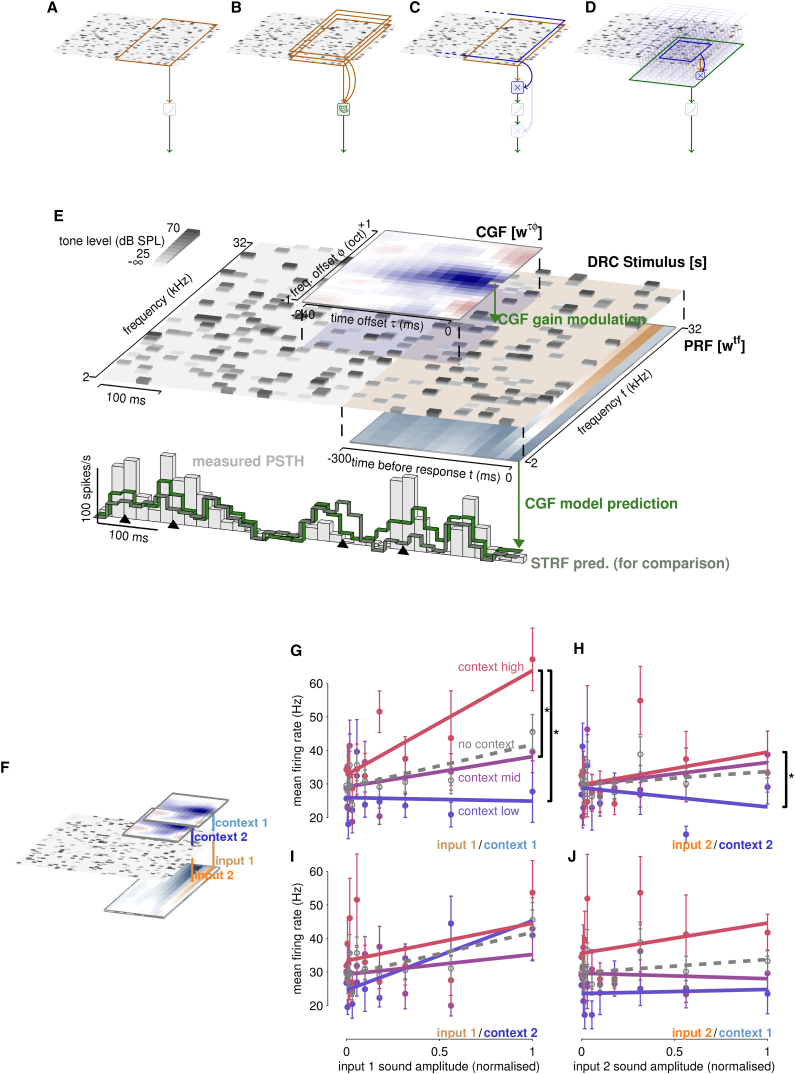
Local Context Shapes Input-Specific Gain (A–D) Cartoon illustrations of receptive field integration mechanisms. (A) In the most basic scheme, input stimuli (gray-level spectrogram) are integrated by a single set of fixed weights (orange). Pointwise nonlinear transforms may apply to each specific input (not shown) or to the integrated weights (light green). (B) Multidimensional LNP models include a small number of differently weighted overlapping integration fields, with outputs combined by a multi-imensional nonlinearity (green). Methods such as MID and STC are designed to characterize such models. (C) Normalization, or other variable global gain, involves the output of one field (blue) modulating the gain of the integrated response to the other (orange). The normalization field may extend well beyond the integration field, so that the effective gain reflects global statistical properties of the stimulus. A further nonlinear transformation (light green) may act before or after gain modulation (light blue). (D) In the phenomenon described here, local context (blue) around each input shapes the gain of response to that specific input. Each input experiences a different context and thus a potentially different gain. Gain-modulated inputs are integrated (green), with a possible further nonlinear transformation (light green). (E) The CGF model. The contextual input-specific gain model incorporates two sets of time-freqency weights. The Principal Receptive Field (PRF; $w^{tf}$) describes the basic sensitivity of the neuron to spectrotemporal energy at all frequencies within a short time window, analogous to the STRF. The Contextual Gain Field (CGF; $w^{\tau \phi }$) describes how each sensitivity is modified by its local acoustic context. The model can be viewed as acting in two stages. First, the stimulus spectrogram is convolved with the CGF in both time and frequency to estimate the local input-specific gain at each spectrotemporal point (upper green arrow). The local stimulus power is then scaled by the corresponding gain and these scaled values, weighted by the PRF, are summed to model the neural response (lower green arrow). The measured response (peri-stimulus-time histogram or PSTH) for one example cortical neuron is shown (gray bars) along with the rates predicted by the CGF model (bright green) and an unmodified STRF (dull gray-green). Differences in prediction (black *triangles*) show that local contextual gain effects both increase and decrease firing rates relative to the STRF model of static sensitivities. (F–J) Local input-specificity of contextual gain effects. The relationships between the measured responses of one example unit and the sound level at two spectrotemporal locations within the unit’s PRF far enough apart in time and frequency to be subject to different local sound contexts (F) are shown without reference to local context (gray open circles and dashed lines); sorted by whether the integrated contextual energy in a local window around that spectrotemporal location fell within a low, middle or high quantile (G and H, colored circles and lines); or, as a control, sorted according to “distant” contextual energy — i.e., integrated energy around the other of the two input locations (I and J, colored circles and lines). Error bars indicate standard error in the mean; lines are fit to the empirical data. The slopes of the input-response relationships differ when sorted by local spectrotemporal context (black bars with asterisks indicate significance), but not when sorted by contextual energy at the spectrotemporally distant location.

For decades, the linearly weighted receptive field has been used to describe sensory neural responses to complex stimuli. Neurons in the central auditory system integrate sound over time and frequency, making the linear model of choice the spectrotemporal receptive field or STRF (e.g., Aertsen et al., 1980, Eggermont et al., 1983, Eggermont, 1993, deCharms et al., 1998, Depireux et al., 2001). Features of the STRF have been used to investigate neural representations in different brain areas (Nelken et al., 1997, Miller et al., 2002, Escabi and Schreiner, 2002, Linden et al., 2003, Woolley et al., 2005), and changes in the shape or overall gain of the STRF have been used to examine how auditory encoding varies with stimulus type (Gill et al., 2006), sound density (Blake and Merzenich, 2002, Valentine and Eggermont, 2004), spectrotemporal contrast (Rabinowitz et al., 2011, Rabinowitz et al., 2012), and behavioral task (Fritz et al., 2003, Fritz et al., 2007, David et al., 2012). One or more STRF-like weighted fields also lie at the heart of linear-nonlinear (LN) cascades, including generalized linear point-process models (Chornoboy et al., 1988) and linear-nonlinear-Poisson models estimated by spike-triggered characterization, maximally informative dimensions and similar methods (Figures 1A–1C; for reviews, see Schwartz et al., 2006, Paninski et al., 2007, Sharpee, 2013).

Despite its wide use, the STRF is known to be an incomplete description of neural responses. Linear STRF predictions capture less than half of the reliable response variance to a complex stimulus in the primary auditory cortex, even without adaptive or task-dependent changes (Sahani and Linden, 2003, Machens et al., 2004). More fundamentally, the crucial assumption of linear weighting—that the sensitivity of the neuron to a local element of the stimulus is independent of the rest of the stimulus—is challenged by many reports of nonlinear combination sensitivity. Such nonlinearities include “forward suppression” of the response to the second tone in a pair (Brosch and Schreiner, 1997, Wehr and Zador, 2005), more complex combination effects for spectrally offset tone pairs (Kadia and Wang, 2003, Sadagopan and Wang, 2009), quadratic sensitivity to the distribution of spectral energy in random-spectrum noise (Yu and Young, 2000, Young and Calhoun, 2005), and nonlinear sensitivity to parts extracted from simple vocalizations (Bar-Yosef et al., 2002, Bar-Yosef and Nelken, 2007).

How do these, and perhaps other, nonlinearities combine over frequency and time to shape responses to complex sounds at different stages of auditory processing? Are time-frequency sensitivities modified substantially by these nonlinear local interactions? Or might the local contextual nonlinearities average away to leave a broadly linear response that is qualitatively, if not quantatively, well described by the STRF?

To address these questions, we extended the multilinear model of Ahrens et al. (2008a) to study the impact of local acoustic context on cortical and thalamic responses.

## Results

### Modeling Local Contextual Input-Specific Gain

We modeled responses of neurons in the auditory cortex and thalamus to statistically stationary, spectrotemporally rich, dynamic random chord (DRC) stimuli using a multilinear approach in which local acoustic context could modify the sensitivity of the neuron to sound level (Figure 1D). The model, as we applied it, combines two matrices of weights. The first is an STRF-like principal receptive field (PRF; $w^{tf}$) with weights defined in absolute frequency and time-lag preceding the response. These weights represent the spectrotemporal sensitivities of the neuron in the absence of local contextual influences. In principle, they would correspond to responses evoked by brief isolated tones with no acoustic energy at nearby frequencies and times—although they were fit using responses to the rich DRC stimulus. These PRF sensitivities are multiplicatively modulated through the action of the second matrix, a contextual gain field (CGF; $w^{\tau \phi }$) with weights defined in terms of *relative* offsets of time (τ) and frequency (ϕ). The CGF defines an acoustic neighborhood or local context around each time-frequency element or “tile” of the discretised stimulus spectrogram. The pattern of energy that falls within that neighborhood is weighted by the entries of the CGF and summed, and this term then multiplies the effect of the energy within the anchoring time-frequency tile on the neural response (Figure 1E), providing “input-specific gain.”

Thus, for a sound with spectrotemporal energy at time t in frequency channel f given by s(t,f), the modeled firing rate $\hat{r}$ at time i was expressed by the equation

(1) $$\hat{r}\left(i\right)=c+\sum_{j=0}^{J}\sum_{k=1}^{K}w_{j+1,k}^{tf}s\left(i-j,k\right)\left(1+\sum_{m=0}^{M}\sum_{n=-N}^{N}w_{m+1,n+N+1}^{\tau \phi }s\left(i-j-m,k+n\right)\right),$$

where the constant c sets a baseline firing rate. The zero-offset CGF weight $w_{1,N+1}^{\tau \phi }$ (note the unconventional summation limits for the indices m and n) was fixed to 0 so that no time-frequency energy contributed to its own context, preserving a linear model response to isolated tones.

The CGF in this model sets a different context-dependent gain at each spectrotemporal tile of the stimulus. This input-specific gain enhances or suppresses the PRF-mediated effect of the stimulus but, provided that the term in parentheses in Equation 1 remains positive, maintains its sign. Thus, the sign of a CGF weight, unlike that of a PRF or STRF weight, does not directly indicate whether sound energy excites or inhibits the neuron. Instead, a positive CGF weight at a particular time-frequency offset indicates that if an input within the PRF were paired with energy only at this relative offset, then the gain with which the PRF-input influenced firing is boosted above 1; thus, for a positive PRF weight firing would be further enhanced, whereas for negative PRF weights, activity would be more suppressed. The obverse holds if the CGF weight is negative; gain is reduced and so the input within the PRF would drive less excitation if positive, and less inhibition if negative. In a complex stimulus, the influence of energy at all offsets around each input in the PRF is linearly combined through the CGF to yield a single gain for that specific input, and the gain-modulated inputs are linearly combined through the PRF to model the neuronal firing rate.

We fit the CGF model to DRC-evoked responses recorded extracellularly from neurons in the auditory cortex and thalamus of anaesthetised CBA/Ca mice. The final analysis database included 64 prolonged continuous recordings from auditory cortex and 101 from auditory thalamus. Cortical recordings corresponded to a subset of the DRC stimulus recordings previously used for STRF analysis by Linden et al. (2003); see Experimental Procedures for details.

### Input Gain Is Specific to Local Context

We found that local context played a substantial role in shaping input-specific gain. To illustrate the effect, we chose two spectrotemporal positions within the responsive region of an example unit’s STRF, separated by about an octave to minimize overlap in local context (Figure 1F). Plots of the average response as a function of the sound level at each of these two positions revealed roughly linear relationships, the (positive) slopes of which were essentially unregularised estimates of the corresponding (excitatory) STRF weights (Figure 1G–1J, “no context,” gray). We then asked whether the slope of this relationship at each time and frequency could be modulated by acoustic context either immediately surrounding the specific chosen time-frequency input or distant from it. We calculated, moment by moment, the integrated sound energy within a local window surrounding each of the chosen time-frequency points, weighted using a CGF estimated by a cross-validation procedure (see Supplemental Experimental Procedures 2). When the integrated energy at position 1 was within the bottom third of its range (“context low,” blue) the slope of the stimulus-response relationship fell to almost 0; if in the middle third (“context mid,” magenta) the slope was roughly the same as when context was ignored; and if in the highest third (“context high,” red) the gain was boosted substantially (Figure 1G) and significantly (permutation tests: low to mid, p=0.20; mid to high, p=0.027; low to high, p=0.0030). The same trend was evident in the relationships between the response and the sound level at position 2, when grouped by the integrated contextual energy at position 2 (Figure 1H; low to mid, p=0.073; mid to high, p=0.38; low to high, p=0.041). However, the slope of the response to sound level at input 1 did not vary with the context at position 2 (Figure 1I; low to mid, p=0.90; mid to high, p=0.35; low to high, p=0.81), nor vice versa (Figure 1J; low to mid, p=0.63; mid to high, p=0.12; low to high, p=0.21).

Thus, only local, not distant, acoustic context affected the gain with which a specific time-frequency input drove firing. This observation is inconsistent with a single STRF-like integration field followed by a static output nonlinearity (Figure 1A) or modulated by a single global gain factor (Figure 1C). It also argues against the sufficiency of a low-dimensional LN model (Figure 1B), as the input-specific context could only be captured by a separate linear filter around each input. However it does not necessarily require that each local context filter is a translated copy of the same CGF weights. This assumed structure (Equation 1) was tested by explicit comparison to alternative nonlinear models described later.

### Contextual Input-Specific Gain Shapes Cortical and Thalamic Responses

Before evaluating the CGF model against nonlinear alternatives, we measured the contribution of contextual input-specific gain modulation to neuronal output by quantifying predictive accuracy relative to the linear STRF model. In doing so, it was necessary to rule out the possibility that any improved prediction came from “overfitting” of the additional parameters of the CGF. We used two approaches.

**Figure 2:**
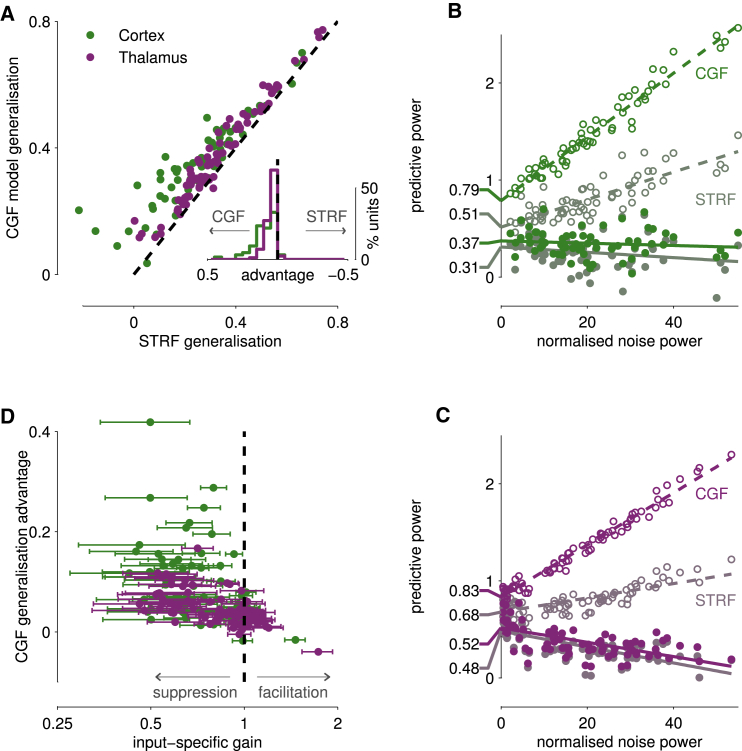
Contextual Input-Specific Gain Shapes Both Cortical and Thalamic Responses (A) Scatterplot of generalization performance for the CGF and STRF models in cortex and thalamus measured by cross-validation; inset shows histogram of differences in favor of the CGF model (left) or STRF model (right). Black dashed lines indicate equal performance. The CGF model almost always generalizes more accurately than the STRF, showing that contextual input-specific gain plays a substantial role in shaping responses in both brain structures. (B and C) Predictive power extrapolations for CGF model (bright colors) and STRF model (dull, greyed colors) in cortex (B) and thalamus (C). Filled circles and solid lines indicate generalization performance on test data, assessed by cross-validation; open circles and dashed lines show predictive performance on training data. In the zero-noise limit, extrapolated intercepts (indicated on the left) are all higher for the CGF model. See Supplemental Experimental Procedures for further explanation. (D) Effective input-specific gains and predictive advantage. Each dot and horizontal bar indicates the median and interquartile range of the distribution of effective input-specific gains across all points in the stimulus for one neuron, obtained by convolving the spectrogram of the DRC stimulus with the neuron’s CGF (see also Figure 3). Median input-specific gains tend to be substantially smaller than 1 and interquartile ranges are often large, indicating that effects of local acoustic context are predominantly suppressive but can vary substantially across spectrotemporal points within the DRC stimulus.

First, we compared the generalization performance of the CGF and STRF models in individual neurons, cross-validating by repeatedly fitting each model to one section of response (“training data”) and evaluating performance on another (“test data”). The added CGF parameters always enable an apparently better fit to the training data. However, if local context were unimportant, then improvement would come only from overfitting to random fluctuations, and would not extend to the unrelated fluctuations of the test data. Indeed, the overfit model parameters would generate perturbed predictions, lowering cross-validation accuracy below that of the STRF. In fact, we found the opposite: the CGF model outperformed the linear STRF model in cross-validation for almost every neuron (Figure 2A), suggesting that local contextual modulation of input-specific gain does indeed reliably shape responses to complex sounds.

Second, we followed Sahani and Linden (2003) to obtain population-level predictive performance estimates for both models. When expressed as a proportion of the estimated stimulus-dependent signal power (see Supplemental Experimental Procedures 3), performance on both training data and test data—assessed by cross-validation—depended systematically on the amount of variability or “noise” in the recording (Figures 2B and 2C). Each of these relationships could be extrapolated to yield “zero-noise” predictive power limits, effectively averaging across the population while discounting the variable impact of noise on each unit. On the training data, the extrapolated value eliminates contributions from overfitting to random fluctuations but may still reflect overfitting to the details of the particular stimulus segment used for training. The equivalent value on test data also minimizes the impact of random fluctuations on model fits but retains any generalization penalty resulting from estimation of the model parameters from finite data. Thus, the two extrapolated limits bracket the true average predictive power of the model class.

Both training and test extrapolated values were consistently higher for the CGF model than for a linear STRF model ([test, training] values were as follows: cortex CGF = [0.37, 0.79], STRF = [0.31, 0.51]; thalamus CGF = [0.52, 0.83], STRF = [0.48, 0.68]; see Figures 2B and 2C). Taking the midpoints of these ranges, we find that modeling the variation in contextual input-specific gain provides a 41% boost in predictive power over the linear STRF relationship in cortex, and a 16% boost in thalamus.

### CGF Model Outperforms Related Second-Order Models

In designing the CGF model to capture the phenomenon of contextual input-specific gain modulation as simply and tractably as possible, we made three key simplifying assumptions: first that energy in the local context is integrated linearly within the CGF, second that the output of this CGF-weighted sum linearly affects the input gain, and third that the CGF weights are the same at each point in the PRF. The result is the multiplicative model of Equation 1, in which the dependence of the firing rate on the sound energy is quadratic. This model thus represents a constrained second-order Volterra expansion, in which the linear kernel is the PRF, and the quadratic kernel is formed from suitably selected products of weights in the PRF and CGF. Using the same CGF weights at each PRF input reduces the total number of parameters that must be fit (1,046) far below that needed to descibe an unconstrained second-order Volterra expansion using the same window size as the PRF (just under 52,000). Prohibitive volumes of physiological data would have been required to fit the unconstrained model.

The single-CGF assumption was supported by the observation that in a dual-CGF version of the model, with potentially different CGFs fit to two pre-selected portions of the PRF—for example, to the excitatory and inhibitory regions—the two learnt CGFs were consistently similar (Figure S2). The dual-CGF model is also a second-order Volterra model but enforces slightly less severe constraints on the quadratic kernel than the single-CGF model. Despite the added degrees of freedom, the dual model added no further generalization ability.

The CGF formulation was also supported by comparison to a “low-dimensional” quadratic model, similar to that described by Park et al. (2013), in which the second-order kernel matrix is approximated by a sum of vector outer products. The dimensionality is given by the number of products in this sum. A one- or two-dimensional quadratic model has comparable degrees of freedom to the CGF model, but quite different constraints; indeed, it can be viewed as a low-dimensional LN model (Figure 1B) with a second-order polynomial nonlinearity (Supplemental Experimental Procedures 4). Neither one-dimensional nor two-dimensional quadratic models generalized as well as the CGF model, as measured by cross-validation (Figure S3). Indeed, the one-dimensional model also fit the *training data* less well, despite having more than twice the degrees of freedom (720 versus 324). Thus the LN structure of the outer-product quadratic form is not as well suited to capture the stimulus-evoked response even in training data. To outperform the CGF model on the training data it was necessary to include at least two outer products in the quadratic kernel, adding more than four times as many parameters as in the CGF model—and this two-dimensional quadratic model did not generalize as well as the CGF model, even after regularization. Noise-discounted extrapolated population values of predictive power for the one-dimensional and two-dimensional quadratic models were ([test, training] values) as follows: cortex 1D quadratic = [0.34, 0.64]; thalamus 1D quadratic = [0.49, 0.75]; cortex 2D quadratic = [0.33, 0.98]; thalamus 2D quadratic = [0.51, 0.99].

Thus, we conclude that the CGF parameterization of the second-order Volterra kernel provides a particularly biologically relevant—and analytically tractable—description of nonlinear constraints on auditory cortical processing.

### Input-Specific Gain Modulation Is Substantial and Predominantly Suppressive

**Figure 3:**
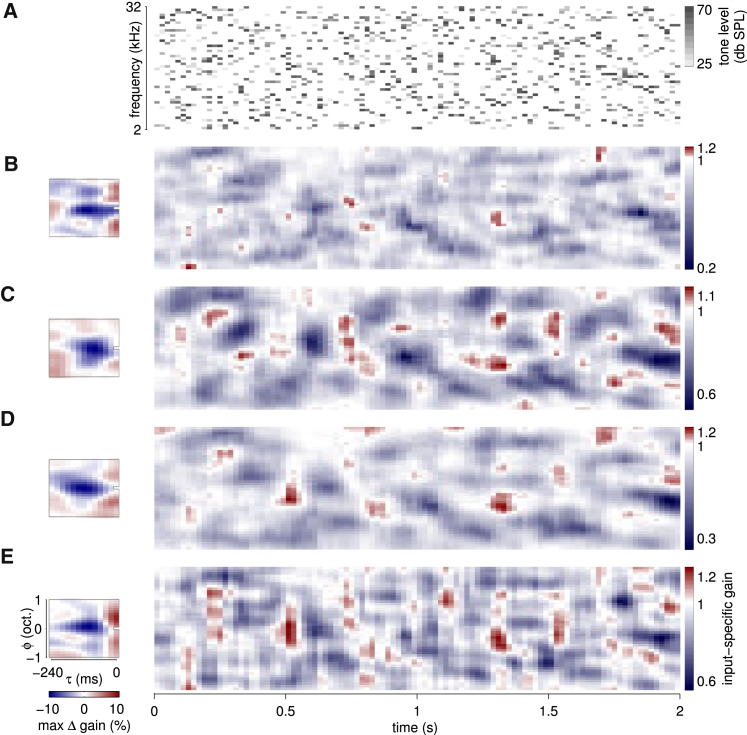
Variation in Contextual Input-Specific Gain across Spectrotemporal Points within a Complex Stimulus (A) Two-second-long segment of the DRC stimulus. (B–E) CGFs (left) for four example cortical neurons are convolved with the spectrogram of the DRC stimulus to reveal effective input-specific gains (right) that vary substantially from cell to cell, frequency to frequency and moment to moment within the stimulus.

The substantial impact of immediate acoustic context on cortical and thalamic responses was also evident in the moment-by-moment variation of input-specific gains inferred by the CGF model (Figures 2D and 3). We convolved the spectrogram of the DRC with each neuron’s CGF, obtaining an estimate of the “effective input-specific gain” set by the local acoustic context at each point in the stimulus. A constant gain of 1 would imply a linear response; effective gains greater than 1 occur at points in the spectrogram where the neuron’s sensitivity is boosted by local acoustic context; and values below 1 occur where sensitivity is locally suppressed. The effective input-specific gain for each neuron varied substantially from moment to moment and frequency to frequency (for examples, see Figure 3), typically ranging between slightly facilitatory and substantially suppressive. Furthermore, differences in CGFs meant that the detailed pattern of gains differed from cell to cell.

To quantify the overall impact and variability of input-specific gain modulation, we computed the quartile points of the distribution of effective gains across the DRC stimulus, both for each neuron individually and for pooled neuronal populations. Gains varied substantially, with large interquartile ranges for most neurons (Figure 2D) and both cortical and thalamic populations (0.37 and 0.39, respectively). Many of these interquartile gain ranges did not include 1 (for 50/64 cortical neurons and 46/101 thalamic neurons), indicating a pervasive and systematic impact of immediate context. In the pooled distributions, the median input-specific gain was significantly smaller than 1 (0.73 in cortex and 0.86 in thalamus; p< machine precision), indicating a predominantly suppressive effect. Furthermore, the predictive advantage of the CGF model over the STRF model increased both as the inter-quartile range of effective input-specific gains increased (i.e., as gain varied more widely over the course of the stimulus; Spearman’s $\rho_{\left(N=165\right)}=0.31$, $p=7.2\times 10^{-5}$), and as the median input-specific gain decreased (i.e., as contextual suppression increased; $\rho_{\left(N=165\right)}=-0.62$, $p=1.6\times 10^{-14}$).

### Two Key Features of Input-Specific Gain Modulation in Cortex and Thalamus

The structures of the CGF and PRF fit to a neuron’s response reveal how input-specific gain depends on local context, and how it interacts with spectrotemporal integration in the neuron. PRFs differed from STRFs in a predictable manner (Figure S4; Supplemental Experimental Procedures 5), and comparisons of either PRF or STRF structure between cortex and thalamus yielded results similar to previous reports for STRFs (e.g., longer receptive field durations in cortex than thalamus; Miller et al., 2002). Our focus here is on novel findings revealed by the CGFs.

**Figure 4:**
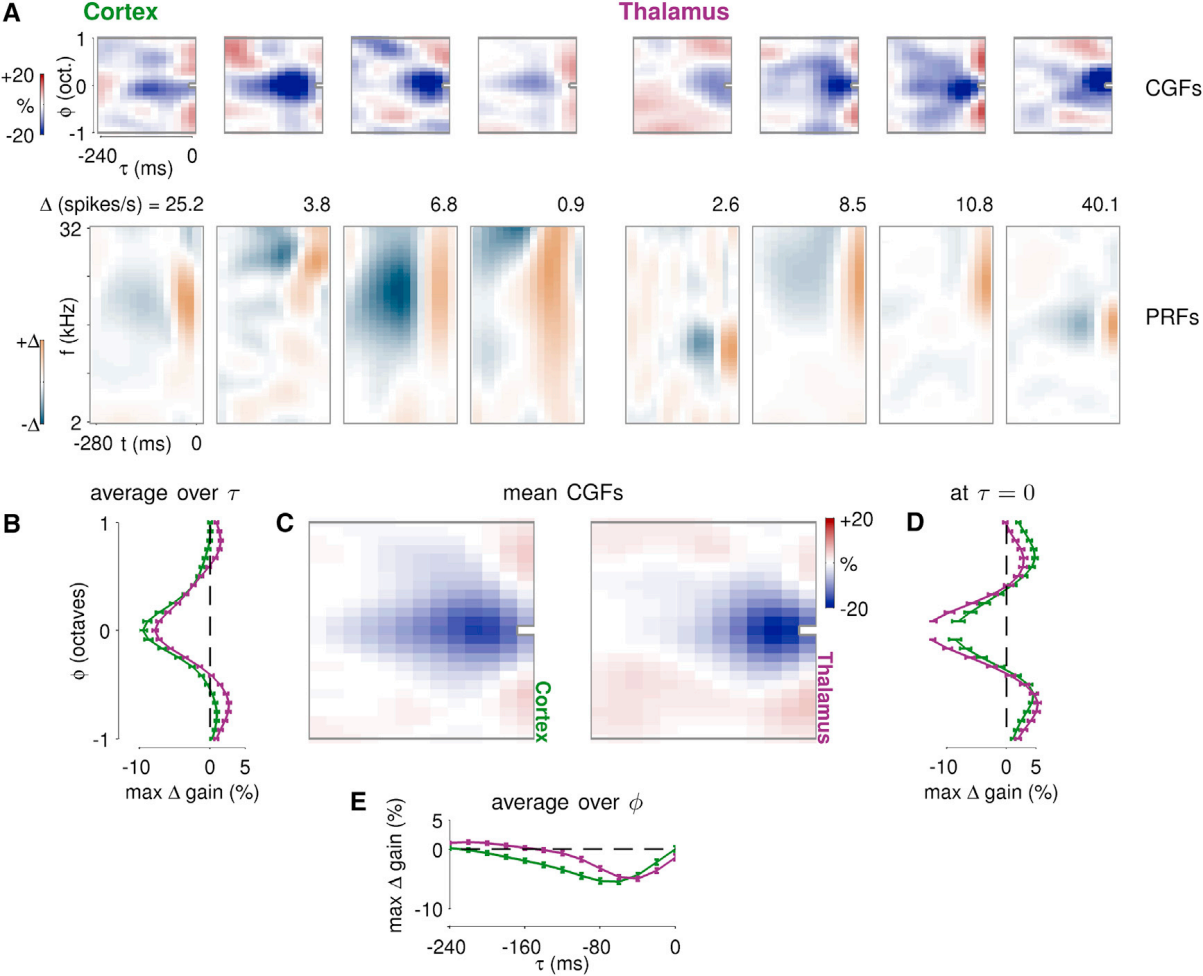
Structure of Input-Specific Gain Modulation in the Cortex and Thalamus (A) Example CGF and PRF pairs for four neural recordings in cortex (left) and four recordings in thalamus (right). CGFs (top) range over relative time τ and relative frequency ϕ. Weights represent the change in gain induced if one of the loudest tones of the DRC stimulus were to fall at the corresponding location, and are shown on common scale (left). PRFs (bottom) range over time t prior to the modeled response and acoustic frequency f (log-spaced). Stimulus modulation of firing differs substantially across neurons, so PRFs are separately (and symmetrically) scaled to the maximum change in firing rate shown above each one. (B–E) Mean CGFs and average profiles in cortex (green) and thalamus (magenta). The central panel (C) shows the spectrotemporal pattern of the mean CGF weights in both structures. The average spectral profiles (B), spectral profiles at 0 delay (D) and average temporal profiles (E) of both means are shown superimposed, with error bars indicating the SE of the estimated population means.

CGFs in both cortex and thalamus displayed consistent features, albeit with different timescales at the two stages of auditory processing. Most cells (examples in Figure 4A) exhibited a suppressive region of negative CGF weights centered at a zero frequency offset, and extending over much of the CGF time window. Thus, preceding sound energy at a similar frequency tended to dampen the impact of a component sound, reducing excitation or inhibition as the component fell within the positive or negative part of the PRF, respectively. This suppressive effect lasted longer in cortex than in thalamus. Also visible in the example CGFs is a halo of gain-enhancing regions around the suppressive center. At long time delays, the structure of these gain-enhancing regions varied considerably from neuron to neuron. However, there appeared to be a consistent enhancement associated with simultaneous or near-simultaneous sound energy at both positive and negative frequency offsets.

Since the CGF ranges over *relative* time and frequency offset (τ and ϕ) it is possible to examine the common stucture of contextual effects by averaging the CGFs of a population of neurons. The two phenomena visible in the examples—delayed suppression and near-simultaneous enhancement—are also evident in the mean CGFs for both cortex and thalamus (Figure 4C), and in the one-dimensional profiles averaged over all τ (Figure 4B), restricted to τ=0 (Figure 4D), and averaged over all ϕ (Figure 4E). Similar results were obtained when the averages were restricted to subsets of neurons grouped by best frequency as estimated from the PRF or STRF (data not shown), demonstrating that the mean CGFs are representative of neurons tuned to all points of the frequency spectrum.

Delayed input-specific gain suppression was centered around a frequency offset of zero in both areas (Figure 4B), but peaked at a greater delay (60–80 ms compared to 40 ms) and extended to longer temporal offsets (160 ms compared to 100 ms) in cortex than in thalamus (Figure 4E). While reminiscent of forward suppression of responses to repeated tones, the modulatory rather than inhibitory effect of the CGF also implies suppression of inhibitory gains, which mght sometimes lead to enhanced responses in complex stimuli. Indeed, the dual-CGF model (Figure S2) confirmed that the contextual influence on inhibitory and excitatory PRF inputs showed similar suppression.

Gain enhancement resulted from sound energy that fell at short time offsets within the CGF window and outside the central suppressive region (Figure 4D). In the cortex this facilitation was clearly strongest and most consistent at time offsets <40 ms, and peaked at frequency offsets of about a half-octave in either direction (Figure 4D). The same short-time effect was also evident in thalamus; indeed, the mean CGF profile at zero time-offset was remarkably similar to that observed in cortex (Figure 4D), with off-frequency peaks approximately a half-octave away from the center. It is difficult to tell from the mean CGFs alone whether these off-frequency peaks reflect a mechanism of gain facilitation specific to half-octave frequency intervals, or whether they emerge from the interplay of two seperate mechanisms: broadband near-simultaneous enhancement centered at zero frequency offset, and narrowband delayed suppression that cancels the enhancement at small frequency and time offsets. However, the observation that similar side-peaks appeared in individual CGFs (Figure 4A) implies that the structure observed in the means does not arise from broad facilitation and narrow suppression contributed by different neurons.

Individual CGFs often showed both narrowband delayed suppression and broadband near-simultaneous enhancement (Figure 4A), suggesting that CGFs are not time-frequency separable. Indeed, predictive power was almost always higher for inseparable-CGF models than for separable-CGF models (Figure S1), despite the expectation that the many more parameters of the inseparable-CGF model would increase susceptibility to overfitting and thereby undermine generalization performance.

### Two Key CGF Features Each Have Significant Impact on Neural Responses

We wondered whether the key features that had appeared reliably in the mean CGFs were each essential for shaping the neural responses, or whether their effects might be substituted by parameters elsewhere in the CGF or PRF. To find out, we refit “elided” versions of the model, where the range of weights corresponding to one of the features was set to zero and the remaining parameters refit to test whether they could compensate for the elision. Generalization performance was compared to that of the full CGF by cross-validation. If the feature removed is not essential, then the elided model should achieve the same generalization performance as the full model despite the feature’s absence. Indeed, with fewer parameters and therefore less risk of overfitting it might generalize *more* accurately than the full CGF. Conversely, if the generalization accuracy after elision is systematically lower, then the impact of the removed CGF feature could not be matched by modifying weights in the rest of the CGF or PRF, and so the feature itself must be essential.

**Figure 5:**
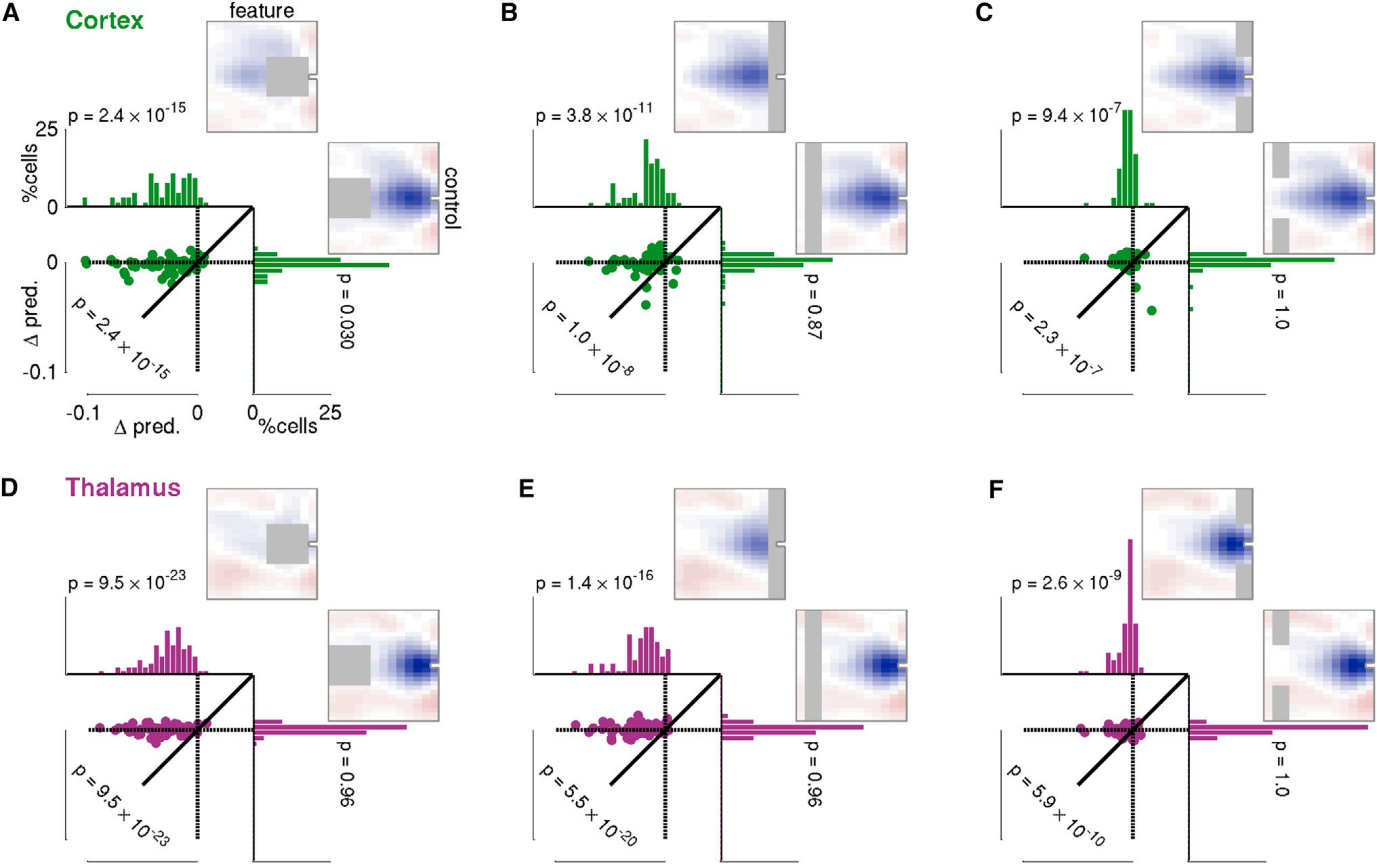
Generalization Disadvantage for Models with Key Features of CGF Elided (A–C) Cortex; (D–F) thalamus. Each panel contrasts the effects of eliding parameters in two identically sized sections of the CGF (gray rectangles): one corresponding to a CGF feature that appeared to consistently shape input-specific gain, the other a control section where CGF weights were inconsistent or small. Weights in the elided regions were fixed at zero, and the model was re-fit to optimize the remaining model parameters. Histograms show distribution across neurons of differences in cross-validation predictive performance (generalization accuracy) relative to the unelided CGF model; p value indicates significance threshold at which the hypothesis that median change in performance equals or exceeds zero can be rejected (one-tailed sign test, uncorrected; *N* = 64 in cortex and 101 in thalamus). Scatter plots compare generalization accuracy of the two elided models neuron-by-neuron; p value indicates threshold for rejection of the hypothesis that median difference for feature elision minus control elision equals or exceeds zero (one-tailed sign test, uncorrected). Across the neural population, elision of key CGF features always resulted in poorer generalization accuracy than that achieved by the full (unelided) model. By contrast, control elisions had significantly less impact; the hypothesis that control elisions produced no reduction in predictive performance could not be rejected in any case after correction for multiple comparisons.

We found that models in which either of the two key CGF features were elided did indeed provide a poorer fit to the data than the unconstrained model (Figure 5). In particular, excluding CGF weights with frequency offsets within 1/3 octave and time offsets between 20 and 120 ms, where delayed suppression is most evident, reduced model predictive power significantly (average difference in cross-validated predictive performance between elided and full CGF models −0.030±0.003 in cortex and −0.027±0.002 in thalamus; Figures 5A and 5D). By contrast, elision of a similarly sized CGF region at much longer delays had no discernable effect at the population level. Eliding CGF weights at all frequency offsets and delays <40 ms, where enhancement is evident, also impaired model fits systematically (change in average cross-validated predictive power −0.017±0.002 in cortex and −0.022±0.002 in thalamus; Figures 5B and 5E). When the elided near-simultaneous region was restricted to short-delay CGF weights with frequency offsets greater than one-third octave, the impact on model predictions was lessened, but remained significant (Figures 5C and 5F). Again, in both cases, elision of a congruent section at much longer delays induced no discernable change in model performance. Thus, both broadband near-simultaneous facilitation and narrowband delayed suppression play significant and independent roles in shaping input-specific gain.

### Detailed CGF Structure Differs between Individual Neurons

Despite their consistent features (Figure 4), the CGFs estimated for individual recordings were not identical. Moreover, at least in the cortex, this neuron-to-neuron variation appeared to contribute to the improved predictive power of the CGF model. Models with neuron-specific CGFs performed at least as well in cross-validation as models with a fixed CGF (Figure S5)—especially for recordings with the lowest normalized noise power. Had neuron-to-neuron variation in the estimated CGF structure arisen solely through noise, the individual CGFs would have overfit and the models performed more poorly.

**Figure 6:**
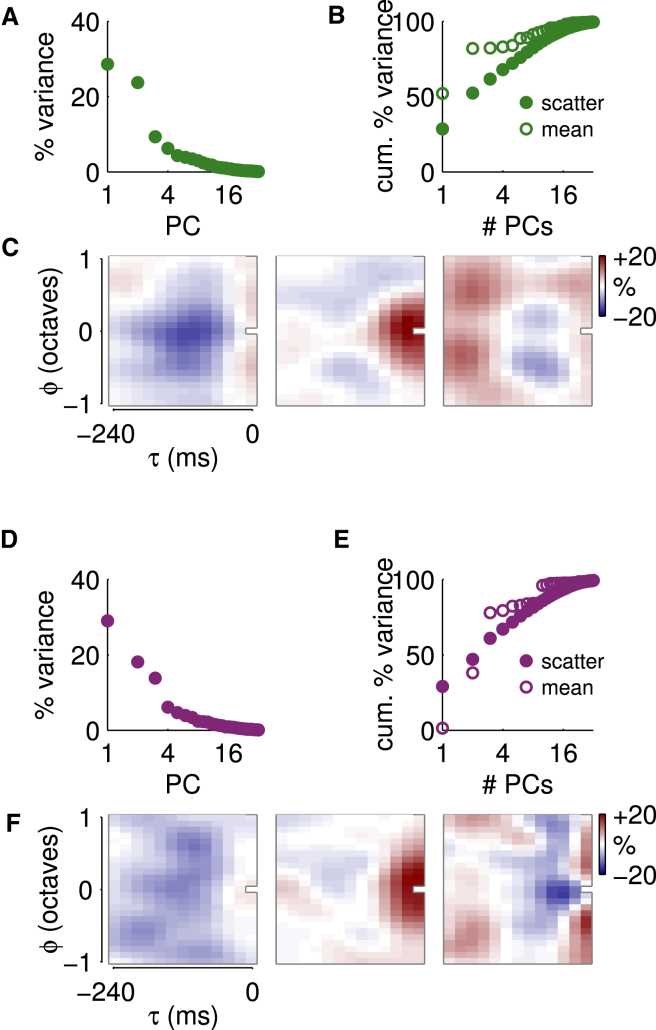
Variability in CGF Structure across Neurons (A–C) PCA of CGFs in the cortex. (A) The absolute variance (i.e., average squared Δ gain) captured by each of the first 32 PCs. (PC numbers are plotted logarithmically.) (B) Filled symbols: fractional variance in the CGFs captured by the leading PCs, as a function of number of PCs considered. Open symbols: fractional sum of squares of the *mean* cortical CGF that projects into the space spanned by the leading PCs, demonstrating how well the variance is aligned with the mean. (C) The three leading PCs in order from left to right. (D–F) PCA of CGFs in the thalamus. Subpanels correspond to (A)–(C).

We investigated the primary modes of variability around the mean by applying principal components analysis (PCA) to the CGFs within the cortical and thalamic populations. PCA decomposes the scatter of a multidimensional dataset into components along which the variability is uncorrelated, and which can then be ranked by the amount of variance that each contributes. In both cortex and thalamus, the scatter around the mean CGF was concentrated in a relatively small number of principal components. In particular, the first two or three principal components (PCs) stood out from the remaining modes (Figures 6A and 6D). Together, the first three PCs described 62% and 61% of the variance in the cortical and thalamic CGFs, respectively (Figures 6B and 6E).

The structure of these first modes of variability for the cortical and thalamic populations is shown in Figures 6C and 6F. In both cases, the dominant effect observed in the first PC was to modulate the overall depth of delayed suppression, either increasing or reducing it as the loading on the PC varied from positive to negative across cells. In the cortex, there was also some suggestion that the strength of this suppression was anti-correlated with broadband simultaneous facilitation, while in the thalamus the two effects were uncorrelated. The second principal mode of scatter in both structures, which carried at least half as much variance as the first in both cases, appeared to modulate the effect on contextual gain of tones at short delays and nearby frequencies. The third mode of scatter was of greater significance in the thalamus (compare Figures 6A and 6D) and reflected variability in the broadband near-simultaneous modulation of input-specific gain.

The subspace defined by the two (in cortex) or three (in thalamus) leading modes of scatter around the population mean CGFs also captured around 80% of the sum of squared weights in the means themselves (Figures 6B and 6E, open circles). This observation (and, indeed, the examples of Figure 4A) suggests that contextual gain modulation involves the interplay of two (or perhaps three) different functional mechanisms contributing in varying degrees to each neuron’s individual contextual gain field, and therefore that the mean CGFs of Figure 4C reflect the average impact of each of these mechanisms across the population.

### Detailed CGF Structure Differs between A1 and AAF, but Not between vMGB and mMGB

**Figure 7:**
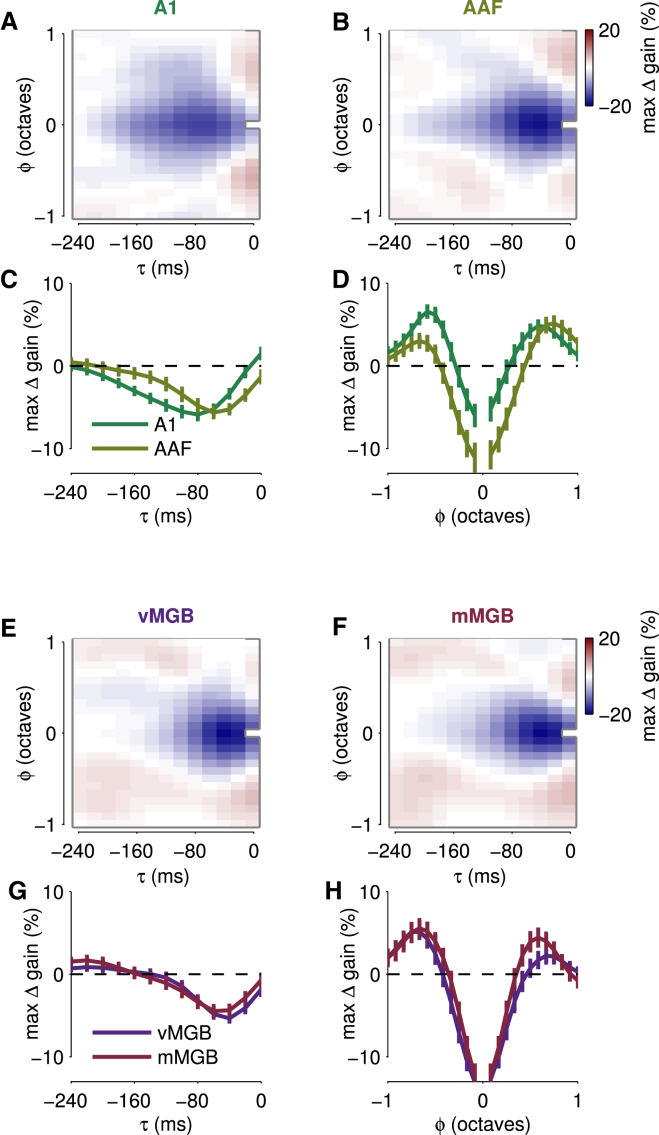
Contextual Input-Specific Gain Compared between Two Cortical Fields and Two Thalamic Subdivisions (A and B) Mean CGFs of neurons in cortical areas A1 and AAF. Overall structure is similar in both areas, but the delayed suppression region is shifted toward shorter delays in AAF. (C) Mean temporal CGF profiles averaged over frequency offset for both areas (error bars show standard errors in the mean). The shorter delay and shorter duration of the suppressive contextual gain effect within AAF is clearly evident. (D) Mean spectral CGF profiles at zero time lag (error bars show standard errors in the mean). The general shape of the spectral interaction is similar in the two cortical areas, although side peaks in AAF fall at slightly larger frequency offsets, perhaps as a result of the stronger short-delay suppression in AAF. (E–H) Similar figures show contextual gain effects in the ventral and medial subdivisions of MGB. No substantial differences are observed between these two thalamic subdivisions.

Our cortical dataset comprised 31 recordings from primary auditory cortex (A1) and 33 from anterior auditory field (AAF), localized physiologically by a reversal of tonotopy (Stiebler et al., 1997, Linden et al., 2003, Guo et al., 2012, Issa et al., 2014). Both A1 and AAF are “core” auditory fields that receive strong thalamic input (Lee et al., 2004, Hackett et al., 2011). Previous studies have revealed differences between A1 and AAF in the temporal extent of STRFs (Linden et al., 2003); these results were confirmed here for PRFs (data not shown). We found that nonlinear context effects also differed between the two cortical areas. Mean CGFs for both A1 and AAF (Figures 7A–7D) exhibited the two key features seen in the overall mean. However, the delayed suppression region of the CGF peaked at smaller delays (60 ms versus 80–100 ms) and was shorter overall (140 ms versus 160–180 ms) in AAF than in A1 (Figure 7C). Spectral profiles for near-simultaneous gain enhancement were similar in shape in A1 and AAF, although the magnitude of the effect appeared stronger in A1, and facilitatory side peaks fell at slightly larger frequency offsets in AAF (Figure 7D).

In contrast, we found no significant differences in detailed CGF structure between two thalamic subdivisions. Our set of 101 thalamic recordings included 51 from the ventral subdivision of the medial geniculate body (vMGB) and 34 from the medial subdivision (mMGB); subdivision assignments were determined histologically through reconstruction of recording sites in sections stained for cytochrome oxidase (Anderson and Linden 2011). (We did not obtain enough recordings from the third major subdivision, the dorsal MGB, to justify including those recordings in the analysis.) The average CGFs in vMGB and mMGB were very similar (Figures 7E and 7F), with overlapping temporal (Figure 7G) and spectral (Figure 7H) profiles. The absence of clear differences in contextual input-specific gain modulation between these two thalamic subdivisions suggests that differences seen in the cortical field averages might arise intracortically.

## Discussion

### Contextual Modulation of Sensory Coding

The neural representation of sensory information is modified by context in many ways. Previous reports have focused on how sensory representations depend on long-term or global stimulus properties or statistics (e.g., Heeger, 1992, Carandini et al., 1997, Schwartz and Simoncelli, 2001, Blake and Merzenich, 2002, Valentine and Eggermont, 2004, Gill et al., 2006, Bar-Yosef and Nelken, 2007, Rabinowitz et al., 2011, Rabinowitz et al., 2012, Mesgarani et al., 2014), or on behavioral or attentional context (e.g., Fritz et al., 2003, Fritz et al., 2007, Atiani et al., 2009, David et al., 2012). By contrast, the current results highlight the dependence of spectrotemporal input-specific gain on fluctuations in immediate local sensory context, even within a statistically stationary stimulus and in an anaesthetised animal.

Changes in global stimulus statistics are necessarily associated with changes in the statistics of local context, and so local modulation may contribute to apparently global effects. For example, the apparent adaptation of STRFs to spectrotemporal density or modulation (Blake and Merzenich, 2002, Valentine and Eggermont, 2004, Gill et al., 2006) may arise in part because denser stimuli drive greater local suppression of input-specific gain across the receptive field (see also Ahrens et al., 2008a; Supplemental Experimental Procedures 5). Similarly, the apparent boost of STRF weights near the spectral edges of a band-limited DRC stimulus, as seen in cat auditory cortex (Gourévitch et al., 2009), may reflect the absence of suppressive drive coming from the part of the CGF around those inputs that falls outside the pass-band of the stimulus. Interactions between contextual input-specific gain modulation and linear STRF estimates may be larger and more idiosyncratic with more structured stimuli, including natural sounds—or even artificial stimuli with nonindependent energy distributions such as spectrotemporal ripples—as nonlinear spectrotemporal contextual effects are then less likely to average away (even if the stimulus set is uncorrelated overall; Christianson et al., 2008).

The local context dependence of input-specific gain may also contribute to some forms of stimulus-specific adaptation (SSA) to tones (Ulanovsky et al., 2003, Ulanovsky et al., 2004, Anderson et al., 2009, Antunes et al., 2010), a phenomenon usually interpreted as arising from long-term stimulus predictability. Although SSA in the cortex and thalamus may persist for a second or more, SSA to tones is strongest at shorter time intervals and develops after only one or two tone repetitions (Ulanovsky et al., 2004, Antunes et al., 2010, Bäuerle et al., 2011). Such rapid stimulus-specific suppression in tone sequences is consistent with the input-specific narrowband delayed suppression observed here with complex stimuli (see also Ulanovsky et al., 2004, Mill et al., 2011, Nelken, 2014).

### Response Nonlinearities

As in many studies of the visual (reviewed by Schwartz et al., 2006, Sharpee, 2013) and somatosensory (e.g., Maravall et al., 2013) systems, responses to complex auditory stimuli have widely been modeled as nonlinear functions of the *output* of one or a few linear STRF-like filter(s) (Figures 1A and 1B). This approach encompasses models of contrast gain control (Rabinowitz et al., 2011, Rabinowitz et al., 2012); LN models derived by spike-triggered covariance (STC), Maximally Informative Dimensions (MID), and similar methods (Atencio et al., 2008, Atencio et al., 2009, Atencio et al., 2012, Sharpee, 2013); LNLN cascade models of excitatory and inhibitory interaction (Schinkel-Bielefeld et al., 2012); and models of noise-invariant cortical responses combining activity-dependent subtractive depression and multiplicative gain control (Mesgarani et al., 2014). These models all start with fixed input fields, with non-linearities acting only after integration. Similarly, studies of adaptive coding (Brenner et al., 2000, Fairhall et al., 2001, Maravall et al., 2007, Mease et al., 2013) have considered context-dependent changes in a single, global, input gain factor (often determined by long-term temporal contrast) that applies after integration but before further nonlinear transformations (Figure 1C). Neither approach captures the input-specific gain modulation described here, in which different context-sensitive input gains act at different points in spectrotemporal space *before integration*. It is likely that both input and output nonlinearities, as well as spike-dependent temporal interactions (Chornoboy et al., 1988, Ahrens et al., 2008b), combine to shape responses in auditory and in other sensory systems.

The closest analogs to the present results use second-order Volterra or similar models to characterize either spectral or temporal nonlinearities (in cortex: Pienkowski and Eggermont, 2010, Pienkowski et al., 2009, David and Shamma, 2013; see also Yu and Young, 2000, in cochlear nucleus). Indeed, the CGF model is a second-order Volterra expansion of *spectrotemporal* nonlinearites, with a constrained quadratic interaction that both provides a ready interpretation of the nonlinearity in terms of modulatory local context effects and keeps the number of parameters within a range that is feasible to fit with limited experimental data. The present study also builds upon previous work by the some of the current authors (Ahrens et al., 2008a), which introduced the multilinear estimation framework and demonstrated the importance of modeling input nonlinearities for accurate prediction of cortical responses to complex sounds. However, these previous studies did not investigate the impact of the full, inseparable, spectrotemporal context—nor compare contextual influence across brain areas. The inseparable spectrotemporal structure of CGFs revealed by the extended model developed here has important implications for understanding auditory processing of complex sounds.

### Implications for Auditory Perception and Neural Processing

#### Narrowband Delayed Suppression

Narrowband delayed suppression in cortical and thalamic CGFs is likely to relate to both the psychophysical phenomenon of forward masking and the physiological phenomenon of forward suppression. In both humans and mice, psychophysical sensitivity to the second of two sounds with similar frequencies is reduced for more than 100 ms after the offset of the first sound (Jesteadt et al., 1982, Walton et al., 1995), consistent with the duration of narrowband suppression seen here in mouse cortex and thalamus. A similar (and putatively related) forward suppression is often observed in central auditory responses to tone pairs (Calford and Semple, 1995, Brosch and Schreiner, 1997, Wehr and Zador, 2005); and although tones played against a *silent* background may also sometimes facilitate later responses (Brosch et al., 1999, Brosch and Schreiner, 2000, Wehr and Zador, 2005), forward suppression appears to dominate in complex *continuous* sounds (e.g., in awake ferrets; David and Shamma, 2013), consistent with our CGF observations.

Forward suppression could arise from direct linear (subtractive) inhibition, output gain (divisive) inhibition, or modulation of input gain. Although measures of suppression in previous studies might include contributions from all three mechanisms, the duration of suppressive effects in awake and anaesthetised animals of many species is similar to that of the contextual influences seen here (Creutzfeldt et al., 1980, Scholl et al., 2010, David and Shamma, 2013). Furthermore, forward suppression in tone pairs appears to outlast inhibitory currents in the target cell (Wehr and Zador 2005) and is specific to particular input synapses (Scholl et al., 2010). Thus, narrowband delayed suppression in the CGF could be an analog, in neural responses to complex sounds, of this input-specific nonlinear component of forward suppression in responses to tones. Such suppression could arise from either synaptic depression or spike-rate adaptation along the auditory pathway. These adaptive mechanisms acting at subthalamic stages of auditory processing (including in the inferior colliculus) might drive narrowband suppression at the shortest timescales in both cortex and thalamus, while differences between cortex and thalamus (and between A1 and AAF) in suppression at longer timescales might reflect the cascaded contributions of thalamocortical and intracortical adaptation. Similar adaptive cascades have been hypothesized to underlie the hierarchical emergence of deviance sensitivity in the central auditory system at much longer timescales (Mill et al., 2011).

#### Broadband Near-Simultaneous Enhancement

The nonlinear augmentation of both excitatory and inhibitory input gain by broadband near-simultaneous sound energy may be a neural correlate of perceptual sensitivity to common onsets. Simultaneous onsets at different frequencies are salient even in a complex sound environment, and guide both auditory stream segregation and object identification (Bregman, 1994, Shamma et al., 2011). While this perceptual phenomenon has long been recognized (Bregman, 1994), a systematic neural correlate has been elusive. Responses to simultaneous tone pairs or complexes display a variety of cell-specific facilitatory and suppressive effects in the auditory cortex (Shamma et al., 1993, Calford and Semple, 1995, Sutter et al., 1999, Kadia and Wang, 2003, Sadagopan and Wang, 2009), arising largely from two-tone (second-order) interactions (Nelken et al., 1994a, Nelken et al., 1994b). Neurons in the auditory cortex and thalamus of the bat show augmented responses to particular combinations of sound frequency corresponding to the harmonics of sonar calls and their echoes (e.g., Suga et al., 1979, Suga et al., 1983, Olsen and Suga, 1991, Wenstrup, 1999). However, no systematic motif of gain enhancement by near-simultaneous pairings has emerged, and it has remained unclear how sound combinations interact with integration within the receptive field. It may be that near-simultaneous gain facilitation becomes significant only during auditory processing of complex broadband sounds (cf. Nelken et al., 1997, Rotman et al., 2001), when the preferential processing of simultaneous onsets may be most functionally important.

It is unclear whether the off-frequency peaks we observed reflect a special sensitivity to half-octave separations or an interaction between a broad central peak of simultaneous enhancement and the earliest component of narrowband delayed suppression. The latter scenario might arise from integration of broadly tuned subthreshold inputs with strong short-term synaptic depression, consistent with reports that short-term spectral integration in the auditory cortex extends over one to two octaves (Kaur et al., 2004, Metherate et al., 2005). On the other hand, neural mechanisms associated with psychoacoustic “critical bands,” which are approximately one-third-octave wide in the mouse (Egorova et al., 2006, Egorova and Ehret, 2008), might conceivably favor nonlinear interactions between sounds one critical band apart. For example, off-frequency CGF peaks might arise from coherent modulations in adjacent frequency laminae of the inferior colliculus, which discretize the midbrain tonotopic gradient into approximately critical-band intervals (Schreiner and Langner, 1997, Malmierca et al., 2008).

#### Variability across Cells and Brain Areas

The shapes of CGFs are notable for their variability as well as for their consistency. Analysis of principal components of the CGFs revealed cell-to-cell variability in the overall depth of delayed suppression and the strength of off-frequency near-simultaneous enhancement. Thus, the consistent features of the mean CGFs are not uniformly inherited from peripheral nonlinearities but arise through the combination of different gain-modulation profiles in individual cortical or thalamic neurons.

The systematic temporal variations in CGFs between thalamus and cortex, and A1 and AAF, are consistent with temporal properties observed in STRFs (Miller et al., 2002, Linden et al., 2003), suggesting that both linear and nonlinear components of forward suppression operate on faster timescales in thalamus than cortex, and in AAF than A1. In contrast, spectral profiles for near-simultaneous gain enhancement were very similar in cortex and thalamus, and also in A1 and AAF, although the magnitude of the effect appeared stronger in A1, and facilitatory side peaks fell at slightly larger frequency offsets in AAF. However, if the apparent peaks arise through a combination of broad facilitatory bumps and the effects of narrowband suppression, then these small differences may simply result from the deeper short-term suppression in AAF.

The consistency of CGFs in ventral and medial MGB accords with the general similarity of many response properties in mouse vMGB and mMGB (Anderson and Linden 2011) but seems surprising given known differences between the subdivisions in sensitivity to stimulus context over much longer timescales (Anderson et al., 2009, Antunes et al., 2010, Antunes and Malmierca, 2011). Thus, the relatively fast context effects studied here may differ from mechanisms of long-term SSA, even if they underlie the short-term component, as we suggest above. Furthermore, consistent contextual gain modulation in auditory thalamic subdivisions also suggests that the CGF differences between cortical fields A1 and AAF (especially in the temporal profiles for delayed suppression) may arise at the thalamocortical synapse or intracortically, rather than from differing patterns of thalamic input.

### Conclusion

The neuronal representation of sound is transformed by nonlinear mechanisms as it ascends the auditory pathway. These mechanisms manifest as the nonlinear interactions within the RFs of neurons in thalamus and cortex captured by the CGF. Similar effects may shape sensory coding in other brain areas and sensory modalities, and adapt across different behavioral contexts.

## Experimental Procedures

Detailed descriptions appear in Supplemental Experimental Procedures 1.

### Surgical Procedures

Subjects were adult male mice of the CBA/Ca inbred strain. Mice were maintained at a surgical plane of anesthesia with ketamine and medetomodine. Cortical surgical procedures were as described by Linden et al. (2003) and conformed to protocols approved by the Committee on Animal Research at the University of California, San Francisco, which were in accordance with federal guidelines for care and use of animals in research in the United States. Thalamic surgical procedures were similar and were performed under a license approved by the UK Home Office in accordance with the United Kingdom Animal (Scientific Procedures) Act of 1986.

### Recording Procedures

Extracellular recordings were obtained from the auditory cortex and thalamus using single or multiple tungsten electrodes, and spike-sorted off-line to extract responses from either small clusters of neurons or well-isolated single units. Cortical areas A1 and AAF were identified physiologically, and thalamic subdivisions vMGB and mMGB were identified histologically.

### Stimuli

All experiments were conducted in a sound-shielded anechoic chamber (Industrial Acoustics). Auditory stimuli were directed toward the ear contralateral to the recording site via a free-field speaker, and a sound-attenuating plug was placed in the ipsilateral ear. Prior to the start of each experiment, acoustic stimuli were calibrated with a Brüel and Kjær 1/4” microphone positioned near the opening of the animal’s auditory canal. Typically, the calibration ensured that the frequency response of the sound system was flat to within ±2 dB over 2–90 kHz.

A 2–32 kHz dynamic random chord (DRC) stimulus described previously by Linden et al. (2003) was used for both cortical and thalamic experiments. While much simpler in structure than many natural sounds, the DRC stimulus can be considered a complex stimulus in that it contains a huge variety of spectrotemporal conjunctions of tonal elements, which provide a substrate for combination-sensitive nonlinearities (such as those captured by the CGF) to act.

### Data Analysis and Modeling

From larger databases of cortical and thalamic recordings collected during presentations of the DRC stimulus, we selected for analysis here those recordings with significantly nonzero stimulus-dependent signal power (see Supplemental Experimental Procedures 3). In order to enable comparisons between brain regions, we further restricted our attention to those recordings that had been reliably localized to A1 or AAF within the auditory cortex, or to vMGB or mMGB within the auditory thalamus. We then fit both linear STRF models and multilinear CGF models to the DRC-evoked neural responses.

## Author Contributions

J.F.L. collected all cortical data, and R.S.W. collected all thalamic data following similar procedures. M.B.A. and M.S. developed the CGF model, and R.S.W. and M.B.A. implemented the parameter identification algorithm used here. All authors designed the analyses, which R.S.W., M.B.A., and M.S. implemented. J.F.L. supervised experimental work, M.S. supervised the model development and implementation, and both oversaw the analysis of the results. M.S., J.F.L., and R.S.W. cowrote the manuscript, with input from M.B.A.

## Supplemental Information

Document S1. Figures S1–S5 and Supplemental Experimental Procedures

Document S2. Article plus Supplemental Information
