# Supplementary material for: Input-Specific Gain Modulation by Local Sensory Context Shapes Cortical and Thalamic Responses to Complex Sounds
Source: Neuron. 2016 Jul 20;91(2):467–81. doi: 10.1016/j.neuron.2016.05.041 (PMC4961224; doi:10.1016/j.neuron.2016.05.041)
Supplement: Document S1. Figures S1–S5 and Supplemental Experimental Procedures [file mmc1.pdf]

Neuron, Volume *91*

Supplemental Information

Input-Specific Gain Modulation by Local Sensory Context Shapes Cortical and Thalamic Responses to Complex Sounds

Ross S. Williamson, Misha B. Ahrens, Jennifer F. Linden, and Maneesh Sahani

Input-specific Gain Modulation by Local Sensory Context Shapes Cortical and Thalamic Responses to Complex Sounds

Supplementary Information

Ross S. Williamson, Misha B. Ahrens, Jennifer F. Linden, and Maneesh Sahani

Supplementary Figures

S1 Predictive performance of separable models (related to Fig. 2a of the main text) 2
S2 The dual-CGF model (related to Fig. 2 of the main text) 3
S3 CGF model performance compared to performance of quadratic models (related to Fig. 2 of the main text) 5
S4 Comparing PRFs and STRFs (related to Fig. 4a of the main text) 6
S5 Contribution of cell-specific CGFs to predictions (related to Fig. 6 of the main text) 7

Supplementary Experimental Procedures

1 Details of experimental procedures (related to Experimental Procedures) 8
1.1 Animals 8
1.2 Surgical procedures 8
1.3 Recording procedures 8
1.4 Stimuli 9
1.5 Data analysis and modelling 9
2 Analysis of input gain specificity (related to Fig. 1 of the main text) 10
3 Evaluating predictive power of STRF and CGF models (related to Fig. 2 of the main text) . . . 10
4 Fitting 1-D and 2-D quadratic models (related to Supplementary Fig. S3) 13
5 Predicting the STRF for a DRC stimulus from the PRF and CGF (related to Supplementary Fig. S4) 14

Supplementary Figures

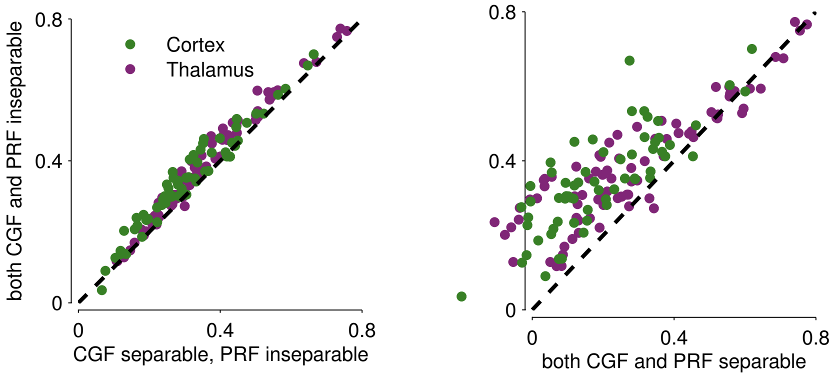

Supplementary Figure S1: Predictive performance of separable models (related to Fig. 2a of the main text)

The generalisation performance (measured as a fraction of predictable response variance accurately predicted during cross-validation) of a model with both CGF and PRF inseparable (ordinate) as in the main text, compared to models where either the CGF alone (left abscissa) or both CGF and PRF (right abscissa) are constrained to be separable. The inseparable model provides a better fit to the contextual input-specific gain modulation than either alternative. In particular, it generalises more accurately despite the many more degrees of freedom that are contained within the two inseparable weight matrices than in their separable equivalents. These added degrees of freedom should allow for greater overfitting in the inseparable model, and thus the size of the true generalisation advantage may be underestimated here.

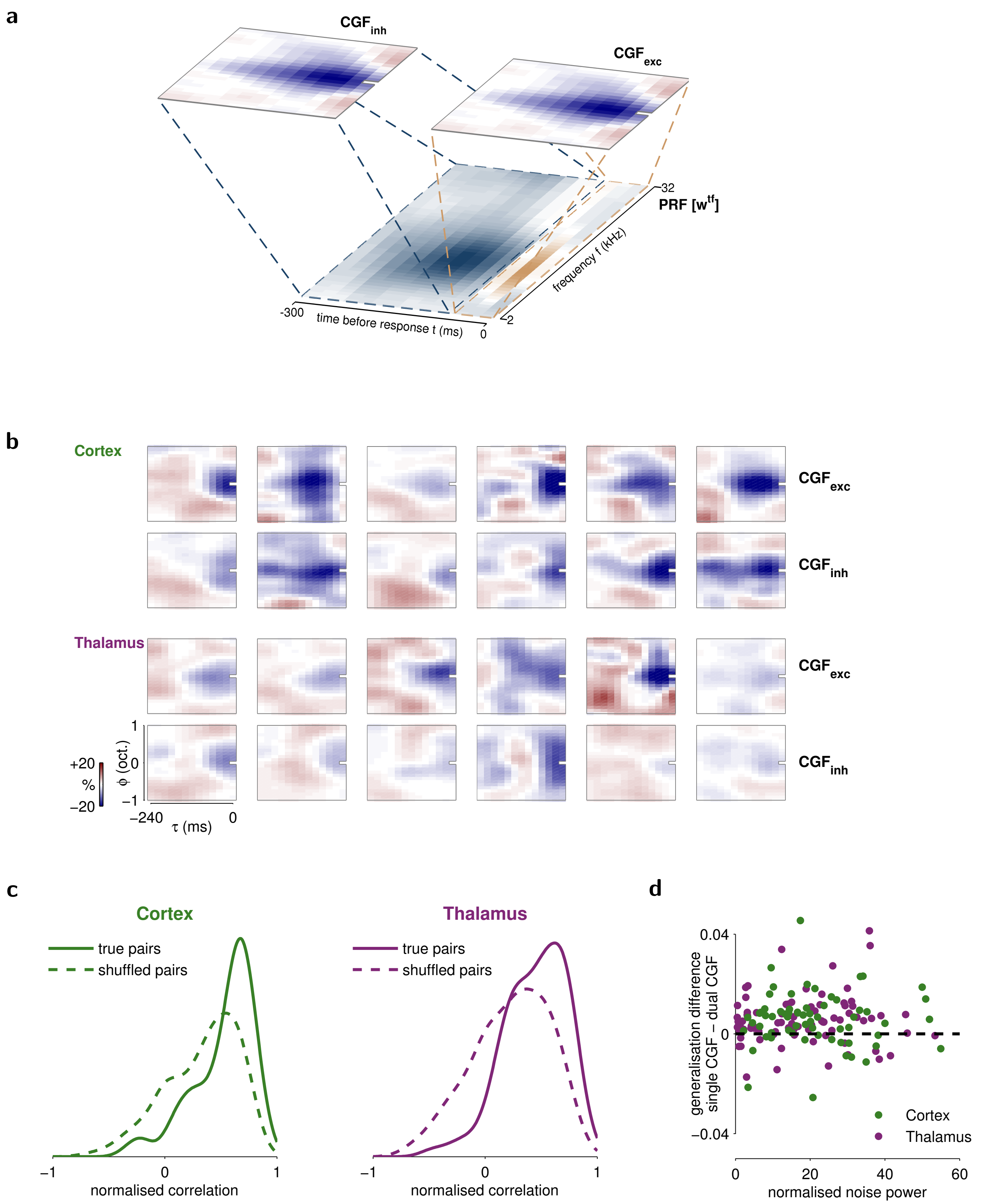

Supplementary Figure S2: Dual-CGF model (related to Fig. 2 of the main text)

(Description on following page)

The model employed in the main text assumed that the form of contextual input-specific gain dependence, embodied in the CGF, was the same at each point within the PRF. We tested this assumption by studying "dual-CGF" models in which the CGF was allowed to differ between two different regions of the PRF. Results are shown here for a model in which the two regions were defined by the timing of PRF excitation and inhibition. (**a**) Illustration of the model. Each PRF (identified by using the standard "single-CGF" model) was divided into two regions along its temporal axis by identifying the point of transition from net excitation to net inhibition. A model was then re-fit with two different CGFs: $\mathsf{CGF}_{\mathsf{exc}}$ applied to the short-latency weights (with net excitation) and $\mathsf{CGF}_{\mathsf{inh}}$ applied to longer-latency weights (with dominant inhibition). (**b**) Example dual CGFs fit to six recordings each in cortex (upper panels) and thalamus (lower panels). Pairs of CGFs look broadly similar, supporting the hypothesis that the form of contextual gain dependence does not differ substantially between the two PRF regions. (**c**) Distribution of correlation coefficients between CGF weights for $(\mathsf{CGF}_{\mathsf{ecx}}, \mathsf{CGF}_{\mathsf{inh}})$ pairs fit to the same recordings (solid lines) compared to the distribution obtained for pairs fit to different recordings (dashed lines). The true pairs are more similar than shuffled ones. (**d**) Difference in generalisation performance (measured as a fraction of predictable response variance accurately predicted during cross-validation) of the single- and dual-CGF models, plotted as a function of recording variability (normalised noise power). The single-CGF model generalises more accurately overall, and even for recordings with low variability, suggesting that the added degrees of freedom in the dual-CGF models lead to overfitting and do not help model the contextual input-specific gain effect more closely. Similar results were obtained when the two CGFs applied to the low-frequency half and high-frequency half of the PRF (not shown). Taken together, these results support the interpretation that a similar pattern of input-specific gain modulation acts upon different regions of the receptive field.

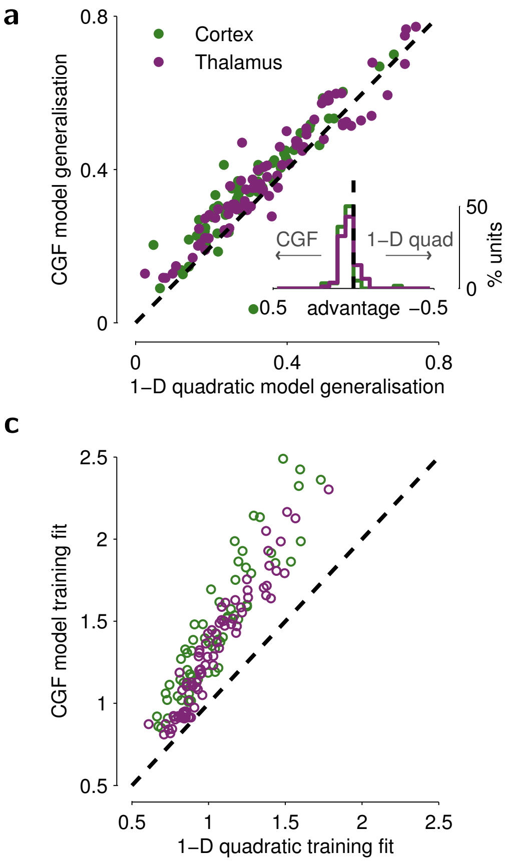

Supplementary Figure S3: CGF model performance compared to performance of quadratic models (related to Fig. 2 of the main text)

(**a**,**b**) Neither a one-dimensional nor a two-dimensional quadratic model generalises as well as the CGF-based context model in cross-validation. Conventions as in Fig. 2a of the main text. Both generalisation performance and training fit (c,d) are normalised by the estimated signal power of the recording. (**c**) The one-dimensional quadratic model accounted for a smaller part of the *training data* signal power than did the CGF model, indicating that the degrees of freedom available within the outer-product Volterra form, while more than twice as numerous than the degrees of freedom of the CGF model (720 versus 324), are nevertheless not as suitably directed to capture the stimulus-dependent neural response even in training data. (**d**) The two-dimensional model, with more than four times as many parameters as the CGF model, did achieve a better fit to the training data — but still generalised more poorly than the CGF model even after regularisation (b), suggesting that the improvement resulted from overfitting.

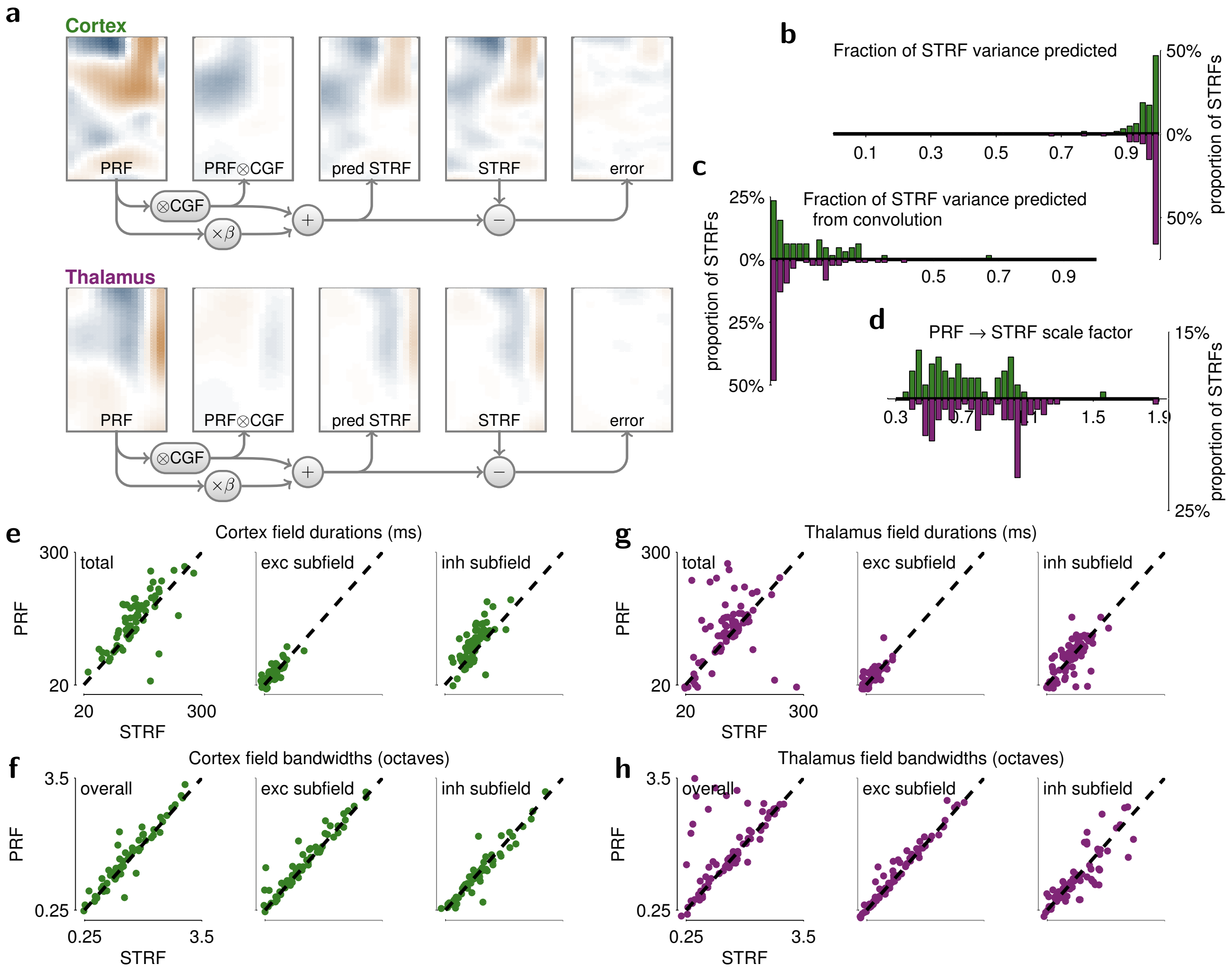

Supplementary Figure S4: Comparing PRFs and STRFs (related to Fig. 4a of the main text)

(**a**–**d**) STRFs estimated using the independent DRC stimulus agree with predictions derived from the estimated CGF model parameters. As derived in Supplementary Experimental Procedures 5, the predicted STRF is formed by combining a copy of the PRF scaled by a factor β with the result of convolving the PRF by the CGF, scaled by the average stimulus strength $\bar{s}$ (a). All panels for each of the two example recordings are shown at the same color scale. The error between the predictions and the measured STRFs is small. Across the populations, the majority of variance in the measured STRFs can be accurately predicted from the CGF model (b). The convolution term generally contributes less than a third of the predicted variance (c), suggesting that changes in STRF shape arising through nonlinear contextual interactions are significant, but contained when using an independent DRC stimulus. More structured or natural stimuli are likely to produce a larger shape difference (Christianson et al., 2008). The scale factors applied to the PRF to form the prediction are generally less than 1 (d), indicating that on average the PRF weights are stronger than the STRF weights. This observation is consistent with the finding that CGF weights are predominantly suppressive.

(**e**–**h**) Comparison of receptive-field extents estimated by the PRF and STRF. Field durations and bandwidths were estimated for the excitatory and inhibitory subfields and for the overall linearly-weighted receptive field using the definitions employed by Linden et al. (2003). Estimated extents were quantised by the stimulus spectrotemporal resolution at 20ms in time and 1/12 octave in frequency, however values were jittered within this quantisation window to aide visualisation of the density of data points. PRF and STRF structure appears broadly similar, although inhibitory subfields tend to be of longer duration in the PRF.

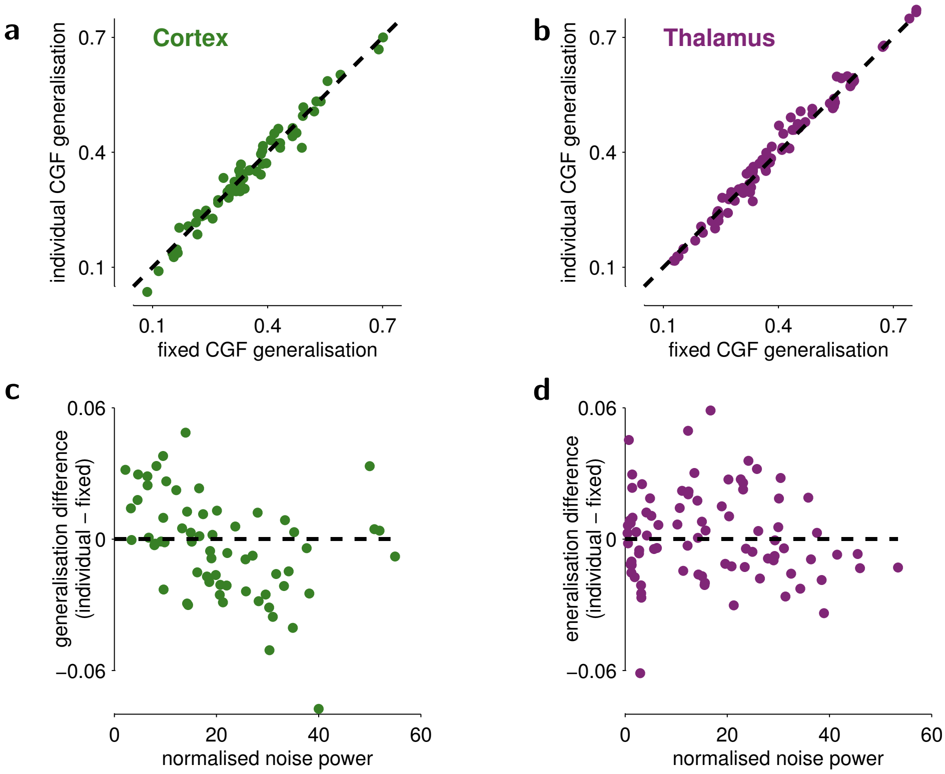

Supplementary Figure S5: Contribution of cell-specific CGFs to predictions (related to Fig. 6 of the main text)

The generalisation performance (measured as the fraction of predictable response variance accurately predicted during cross-validation) of models with an individual CGF fit to each recording as described in the main text, compared to a model in which the CGFs for all recordings were fixed either (**a**,**c**) to the mean CGF for the corresponding cortical field (A1 or AAF) or (**b**,**d**) to the mean CGF in the thalamus (where the subregion means were indistinguishable). Performance of both models was broadly similar across each population, suggesting that the common field-specific contextual gain effects identified in Fig. 7 of the main text play a major role in shaping all gain-sensitive responses. However, the effective number of degrees of freedom available to the population of models with individual CGFs fit to each recording is many times greater than the number of degrees of freedom for models with a fixed CGF for all recordings. The additional degrees of freedom in the individual CGFs will allow for overfitting, and should thus tend to reduce generalisation performance unless these degrees of freedom are also important to modelling the true response. This effect is reflected in a trend (c,d) for individual-CGF models to generalise more poorly than those with a fixed CGF when recordings are more variable (higher normalised noise power), as these are the cases where overfitting is likely to play a more significant role (see also Fig. 2 of the main text). Notably, however, for the recordings with the most reliable stimulus-dependent signal (lowest normalised noise power), generalisation performance was usually better for models with cell-specific CGFs than for models with a fixed CGF, at least in the auditory cortex (c). Hence, we conclude that the individual variation in CGFs within a field does also contribute to shaping sensory responses, but that we did not have the statistical power in the present study to quantify the precise extent of this contribution.

Supplementary Experimental Procedures

1 Details of experimental procedures (related to Experimental Procedures)

1.1 Animals

Twelve adult male CBA/CaJ mice (6–15 weeks old) were used for cortical experiments, and six adult male CBA/Ca mice (6–8 weeks old) for thalamic experiments. These mice are the same CBA/Ca inbred strain, obtained from different vendors; Jackson Labs versus Harlan or Charles River UK. Mice were maintained in standard cages and under standard mouse housing conditions.

1.2 Surgical procedures

Surgical procedures for cortical and thalamic recording experiments were similar to those described previously in Linden et al. (2003). Mice were anaesthetised with ketamine and medetomidine. After an initial intraperitoneal bolus injection of anaesthetic, a cannula was placed into the animal's peritoneal cavity so that maintenance boluses or continuous infusion of anaesthetic could be provided. Dexamethasone was administered to control brain oedema, atropine to minimise bronchial secretions, and Ringer's solution to ensure adequate hydration. The animal was kept on a homeothermic blanket (Harvard Instruments) to ensure that the body temperature was maintained at approximately $37.5 \pm 0.5°C$ (monitored via a rectal probe). Once fully anaesthetised and prepared for surgery, the animal was placed onto a bite bar in order to immobilise its head, after which the skin was transected along the midline to expose the skull.

For cortical experiments, a small craniotomy was performed on the left-hand side of the skull, to expose a region bordered rostrally by the lambdoid suture, caudally and ventro-laterally by the squamosal suture, and dorso-medially by the temporal ridge. Cortical areas A1 and AAF were identified physiologically by reversal of the tonotopic gradient as described previously in Linden et al. (2003).

For thalamic experiments, a craniotomy approximately 2.5 mm in diameter, centred 2.75 mm lateral to midline and 2.75 mm caudal to bregma, was performed on the right-hand side of the skull, enabling vertical access to the thalamus. Thalamic recording sites were localised to vMGB or mMGB histologically, using procedures similar to those described by Anderson et al. (2009) and Anderson and Linden (2011). Electrolytic lesions were created by passing current through the desired electrode on the array (5μA for 7 secs). Such lesions were typically created at the most medial and lateral electrodes on the array that yielded auditory activity. Lesions were replicated at both the top and bottom of the electrode track. Ideally, this procedure yielded four lesions (two at the top of the track, and two at the bottom), bracketing the area over which auditory activity had been recorded. This placement of lesions allowed for estimation of shrinkage and histological reconstruction of most recording sites.

Once lesioning was complete, animals were euthanised with sodium pentobarbital and perfused transcardially with 4% paraformaldehyde in 0.1 M phosphate buffer. Following perfusion, the brain was removed and placed in the paraformaldehyde solution for 1–2 days. Blocks containing the full auditory thalamus were then cut into $50\,\mu$m slices using a vibratome. The sections were then stained for the metabolic marker cytochrome oxidase (CYO), which delineates auditory thalamic subdivisions. Slides were incubated for 3–7 hours at 37°C in a solution containing 20 mg of diaminobenzidine hydrochloride in 10 ml of distilled water and 30 mg of cytochrome c with 3 g of sucrose in 30 ml of 0.1 M phosphate buffer.

Electrolytic lesions were visualised in the stained brain sections using a Zeiss AxioPlan 2 Imaging microscope (magnification x25–x200). The position of each neuron was assigned to the appropriate subdivision as defined by the CYO distribution. Recording sites for which localisation was ambiguous were not included in the subdivided datasets.

1.3 Recording procedures

For cortical experiments, extracellular recordings were made using epoxylite-coated tungsten electrodes (FHC Inc.; 1–4 MΩ impedance). These were introduced into the left auditory cortex in penetrations orthogonal to the cortical surface. Recordings targeted the thalamorecipient layers III/IV (Smith and Populin, 2001) by cortical depth (350–600 μm below the dural surface), and were obtained using clicks and frequency sweeps as search

stimuli. Cortical areas A1 and AAF were identified physiologically by reversal of the tonotopic gradient as described in Linden et al. (2003).

For thalamic experiments, extracellular recordings were made across all thalamic subdivisions using custom-made linear arrays consisting of eight tungsten electrodes (World Precision Instruments; impedance typically 1-2 MΩ). The array was placed perpendicular to the midline with the first penetration targeting a position approximately 2 mm from midline, 3 mm from bregma, and 2200 μm below the cortical surface, as this position was deemed most likely to yield responses from all three major thalamic subdivisions (Anderson and Linden, 2011). Neurons responsive to auditory stimuli were located by their responses to clicks. Once an auditory response had been established (typically at depths of about 2900 μm), further sites were located by progressing the electrode 100 μm at a time, until auditory activity was lost. Histological delineation was carried out as described above to identify subdivision locations for all thalamic recordings, and recording sites for which localisation was ambiguous were not included in the subdivided datasets.

Cortical and thalamic recordings were analysed off-line using Bayesian spike-sorting techniques (Sahani, 1999; Lewicki, 1998) to extract responses from either small clusters of neurons or single units. We used automated clustering criteria to quantify single-unit isolation. Using Bayesian criteria requiring >95% probability of single-unit isolation, only a minority of the recordings in cortex and thalamus were judged to be definitively single units; therefore we conservatively assume here that many recordings were from local multiunits.

1.4 Stimuli

To obtain an initial characterisation of the frequency-intensity response area for each recording site, we used simple tonal stimuli consisting of 50 or 100 ms tone pulses, ramped up and down with 5 ms cosine gates. The frequency and intensity of each tone were varied pseudorandomly over a 2–32 kHz range of frequencies (in 1/12-octave steps) and a 0–70 dB SPL range of in intensities (in 5 dB increments). Other simple stimuli such as clicks, broadband noise, and frequency-modulated sweeps were also used to identify recording sites where auditory activity was present.

We then presented the 2–32 kHz dynamic random chord (DRC) stimulus described previously by Linden et al. (2003). This spectrotemporally rich stimulus is clocked such that every 20 ms a combination of 20 ms cosine-gated tone pulses with randomly chosen frequencies and intensities is generated. Centre frequencies of the tone pulses were chosen from 48 different possibilities (2–32 kHz in 1/12 octave steps). The number of tones that composed a chord was random, with an average density of two tone pulses per octave. The peak level of each pulse was chosen randomly from 10 different intensity levels, 5 dB SPL apart in the range 25–70 dB SPL. A single trial of the DRC stimulus lasted 60 seconds. Full presentation of the stimulus lasted for 20 minutes, allowing for 20 continuous trials. Cortical experiments also involved presentations of a 25–100 kHz version of the DRC stimulus, which was not used in thalamic experiments; cortical recordings using this "high-frequency" DRC stimulus were therefore not included in the analysis here.

1.5 Data analysis and modelling

We fit both linear STRF models and multilinear contextual input-specific gain models to the DRC-evoked neural responses. Estimation of the STRFs was carried out using the automatic smoothness determination algorithm (ASD) algorithm (Sahani and Linden, 2003a). Conceptually, this approach uses regularised linear regression with a smoothness constraint which is optimised separately for each recording.

The contextual input-specific gain model of equation 1 of the main text is bilinear, and was fit using the alternating least squares (ALS) approach of Ahrens et al. (2008). Each least-squares step was regularised using either the ASD-derived optimal spectrotemporal smoothing (for the PRF) or a fixed smoothing bandwidth of 40 ms and 1/6 octave (for the CGF). The fixed CGF smoothing was necessary to facilitate straighforward averaging and comparison of CGF properties across recordings. All PRFs and CGFs shown in this study were regularised in this way. However, training data performance measures in Fig. 2b of the main text and Fig. 2c of the main text were derived from unregularised fits.

Generalisation performance was assessed using ten-fold cross-validation, reserving a randomly distributed disjoint subset of one-tenth of the bins as the validation set for each of the ten repetitions.

2 Analysis of input gain specificity (related to Fig. 1 of the main text)

To illustrate the locality of the input gain effects in an example unit, we chose two spectrotemporal positions over an octave apart within the responsive region of the unit's STRF (Fig. 1f of the main text): input 1 had a temporal offset of 20 ms and a centre frequency of 31.09 kHz ($j_1 = 2; k_1 = 48$) and input 2 had a temporal offset of 40 ms and centre frequency of 13.85 kHz ($j_2 = 3; k_2 = 34$). For each input, we first calculated the average number of spikes observed when the sound amplitude at that point in the STRF took on each of the 11 possible values (including 0) $s_0 \ldots s_{10}$. That is, for each input $p = 1, 2$ and level index $l = 0 \ldots 10$, we averaged the responses $r(t)$ at all times at which the DRC stimulus took on the level s_l at the pth input location:

$$\bar{r}_p(s_l) = \langle r(t) \rangle_{t\,:\,s(t-j_p,k_p)=s_l} \,.$$

The slope of the linear relationship between $\bar{r}_p(s_l)$ and s_l is essentially an unregularised estimate of the corresponding STRF weight (Fig. 1g–i of the main text, grey open circles and dashed lines), that is the gain with which the unit responds to this particular input.

We then asked how this response gain was affected by local and remote context. We fit the context model to this unit, and computed the predicted gain modulation $G(i, k)$ at each time i and frequency k in the stimulus. (Similar values are illustrated in Fig. 3 of the main text.) To avoid risk of overfitting, for this analysis we estimated the gain modulations using a cross-validation scheme: that is, the gain at time i was estimated using a CGF derived from a cross-validation fold in which the training data did not include time i. We divided the values $G(i, k)$ into three equal-sized quantile sets — $\mathcal{Q}_{\text{low}}$, $\mathcal{Q}_{\text{mid}}$ and $\mathcal{Q}_{\text{high}}$ — and repeated the averages, now selecting for times when either the local or the remote context fell in a specific quantile. That is, for input $p = 1, 2$ and context $q = 1, 2$ we found

$$\bar{r}_{p,q\ \text{low}}(s_l) = \langle r(t) \rangle_{t\,:\,s(t-j_p,k_p)=s_l \text{and} G(t-j_q,k_q)\in\mathcal{Q}_{\text{low}}} \,,$$

along with similar averages for $\mathcal{Q}_{\text{mid}}$ and $\mathcal{Q}_{\text{high}}$. These values, along with the corresponding linear relationships, are shown in Fig. 1g–j of the main text. As described in Results, we then tested hypotheses regarding changes in the slopes of these linear relationships. The significance of the changes in slope was assessed by comparing each observed difference in slopes to a simulated null distribution of differences constructed by permuting the time indices of the predicted gain values $G(i, k)$ and then repeating the analysis. The p-values quoted are the proportion of 1000 simulations on which a larger difference in slopes was observed after permutation.

Note that even if context had no effect on input gain, our analysis could generate a change in intercept in the $\bar{r}_{p,q}$ relationships. This is because the CGF applied around (say) location 1 is not orthogonal to the local part of the STRF, and so the local predicted gain will be correlated with the linear input integrated over that local region. Thus, restricting to times when $G(t - j_1, k_1) \in \mathcal{Q}_{\text{low}}$ implicitly selects times when the local linear input around location 1 is low. However, in a linear model this effect must be additive and independent of the level at input 1 (and of that at input 2, given that it is an octave away). Thus it would lead to a constant offset in the linear relationships, not to a change in slope.

3 Evaluating predictive power of STRF and CGF models (related to Fig. 2 of the main text)

This section of Supplementary Experimental Procedures provides a more detailed explanation of the methods used in Fig. 2 of the main text for evaluating predictive power of STRF and CGF models. This approach was originally introduced by Sahani and Linden (2003b).

Intuitions underlying the approach

The variability of neural responses to a repeated stimulus leads to two difficulties in evaluating the predictive performance of a stimulus-response function model such as an STRF model or CGF model. First, the variability obscures the desired target for the model output. Perfect prediction of a noisy response is impossible, even in principle; moreover, since the true underlying relationship between stimulus and neural response is unknown, it is unclear what degree of partial prediction could possibly be expected. Second, noise introduces error into the estimation of the model parameters themselves (e.g., the STRF weights, or in the CGF model, the PRF and

CGF weights). Consequently, the estimated model parameters will inevitably differ from the "ideal parameters" that would have been obtained in the absence of noise, and response predictions from the estimated model will therefore understate the predictive performance that those ideal parameters might have achieved.

These difficulties are both manifest in a classical statistical measure of goodness-of-fit: the coefficient of determination, or r^2 statistic. This is the ratio between the reduction in variance achieved by a regression model (the total variance of the measured outputs minus the variance of the residuals) and the total variance of the measured outputs. The total variance of the outputs appearing in the denominator includes a contribution from the noise, and so an r^2 of 1 is an unrealistic target and the actual maximum achievable value of r^2 is unclear. Moreover, the reduction of variance obtained using the same data as were employed to to fit the model parameters (the "training data"), which is the factor that appears in the numerator of r^2, includes some "explanation" of noise due to the phenomenon of overfitting, where a chance partial correlation between the model inputs and the noise allows the model to fit these elements of the variability.

Following Sahani and Linden (2003b), we take an alternative approach in this study, compensating for the disadvantages of r^2 in three key analytic steps that overcome the confounding effects of neural response variability on model evaluation and model estimation. First, we derive an unbiased estimate of the total *predictable* (stimulus-dependent) component of the variance in the neural response (see the section on maximum predictable power below). Second, we assess model predictions relative to this noise-independent standard, both on the training data used to estimate the model parameters (as for the r^2) and on test data not used to estimate the parameters, using the standard procedure of cross-validation, described below. For any single recording, the predictive performance of the estimated model on training data and on test data provide, respectively, over- and under-estimates of the predictive power of the version of the model with ideal parameters. When the trial-to-trial variability of the neural response is large, these estimates might bound the predictive power of the ideal version of the model extremely loosely. However, upper and lower estimates of predictive power for a population of similar neural recordings can be extrapolated with respect to the degree of variability in each recording, to obtain an estimate of the fraction of predictable power that would have been explainable given an idealised recording from the same population that exhibited no trial-to-trial variability. In the third and final stage of the analysis, we perform such an extrapolation, to quantify the extent to which either STRF or CGF models can account for auditory cortical and thalamic responses to dynamic random chord stimuli in the zero-noise limit.

Maximum predictable power ("signal power")

In our experiments, a DRC stimulus comprising T random chords was repeated N (= 20) times for each recording. The resulting spike-trains were binned (in 20 ms bins) to yield a set of N response vectors $\{\mathbf{r}^{(n)}\}_{n=1}^{N}$ for each unit, with each response vector formed of T spike counts $(r_1^{(n)}, r_2^{(n)}, \ldots, r_T^{(n)})$. Our objective is to measure the performance of a predictive model in terms of the fraction of *response power* that it successfully predicts, where "power" is used here in the sense of average squared deviation from the mean over time: $P(\mathbf{r}) = \langle (r_t - \langle r_t \rangle)^2 \rangle$, with $\langle \cdot \rangle$ used to denote averages over time. As argued above, only some part of this total response power is predictable, even in principle; fortunately, the magnitude of this *signal power* can be estimated for each neuron by analysing the repeated responses to the same stimulus sequence. We provide here an intuitive derivation for the relevant estimator; see also Sahani and Linden (2003b).

The impossibility of perfect prediction results from the variability in the responses $\mathbf{r}^{(n)}$. To characterise this variability, we divide each response into a reliable and a variable component: $\mathbf{r}^{(n)} = \boldsymbol{\mu} + \boldsymbol{\eta}^{(n)}$, with the variable component $\boldsymbol{\eta}^{(n)}$ defined to have an expected value of zero in every time bin. From the point of view of a predictive model, $\boldsymbol{\eta}^{(n)}$ is unpredictable noise, and indeed we refer to it in the following as "noise" even though it may in fact reflect biologically meaningful but non-stimulus-locked activity. If we could average together an infinite set of responses to the same stimulus, we would obtain the "signal" part $\boldsymbol{\mu}$. This reflects the stimulus-driven response of the neuron under consideration, and is thus the only component that is predictable by a model of the cell's stimulus-response function. However, the average of a finite number of trial responses collected within experimental constraints retains a contribution from the noise, and thus the true signal response $\boldsymbol{\mu}$ cannot be determined. Nevertheless, it is possible to form an unbiased estimator of the power in that response, as follows.

First, the simple property of additivity of variances implies that $P(\mathbf{r}^{(n)}) \stackrel{\mathcal{E}}{=} P(\boldsymbol{\mu}) + \left\langle (\eta_t^{(n)})^2 \right\rangle$ (where the symbol $\stackrel{\mathcal{E}}{=}$ is used to represent "equal in expectation"—i.e., the equality may not hold on any trial, but the expected

values of the left- and right-hand sides are equal). This relationship depends only on the noise component having been defined to have zero expectation, and holds even if the variance or other property of the noise depends on the signal strength as would be expected for a Poisson noise process. We now construct two trial-averaged quantities, similar to the sum-of-squares terms used in the analysis of variance (ANOVA): the power of the average response, and the average power per response. Using $\bar{\cdot}$ to indicate trial averages:

$$P(\overline{\mathbf{r}^{(n)}}) \overset{\mathcal{E}}{=} P(\boldsymbol{\mu}) + P(\overline{\boldsymbol{\eta}^{(n)}}) \qquad \text{and} \qquad \overline{P(\mathbf{r}^{(n)})} \overset{\mathcal{E}}{=} P(\boldsymbol{\mu}) + \overline{P(\boldsymbol{\eta}^{(n)})}.$$

Assuming the noise in each trial is independent, although the noise in different time bins within a trial need not be, we have: $P(\overline{\boldsymbol{\eta}^{(n)}}) \overset{\mathcal{E}}{=} \overline{P(\boldsymbol{\eta}^{(n)})}/N$. Then solving these equations for $P(\boldsymbol{\mu})$ suggests the following estimator for the signal power:

$$\hat{P}(\boldsymbol{\mu}) = \frac{1}{N-1}\left(NP(\overline{\mathbf{r}^{(n)}}) - \overline{P(\mathbf{r}^{(n)})}\right). \tag{S1}$$

A similar estimator for the *noise power* is obtained by subtracting this expression from $\overline{P(\mathbf{r}^{(n)})}$. Both estimators are unbiased, provided only that the noise distribution has defined first and second moments and is independent between trials. Unlike the sum-of-squares terms encountered in an ANOVA, the signal power estimate is not a χ^2 variate even when the noise is normally distributed (indeed, it is not necessarily positive). However, since each of the power terms in Eq. S1 is the mean of at least T numbers, the central limit theorem suggests that $\hat{P}$ will be approximately normally distributed for recordings that are considerably longer than the time-scale of noise correlation (in the experiments considered here, $T = 3000$, equivalent to a duration of 60 s). Its variance is given by:

$$\mathcal{V}ar\left[\hat{P}\right] = \frac{4}{N}\left(\frac{1}{T^2}\boldsymbol{\mu}^{\mathsf{T}}\Sigma\boldsymbol{\mu} - \frac{2}{T}\mu\boldsymbol{\sigma}^{\mathsf{T}}\boldsymbol{\mu} + \mu\sigma\mu\right) + \frac{2}{N(N-1)}\left(\frac{1}{T^2}\mathsf{Tr}\left[\Sigma\Sigma\right] - \frac{2}{T}\boldsymbol{\sigma}^{\mathsf{T}}\boldsymbol{\sigma} + \sigma^2\right), \tag{S2}$$

where Σ is the $(T \times T)$ covariance matrix of the noise, $\boldsymbol{\sigma}$ is a vector formed by averaging each column of Σ, σ is the average of all the elements of Σ and μ is the time-average of the signal $\boldsymbol{\mu}$. Thus, $\mathcal{V}ar\left[\hat{P}\right]$ depends only on the first and second moments of the response distribution; substitution of data-derived estimates of these moments into Eq. S2 yields a standard error bar for the estimator.

In this way we have obtained an estimate $\hat{P}$ (along with corresponding uncertainty) of the maximum possible signal power that any model could accurately predict, having assumed neither a particular distribution nor short-time-scale independence in the noise. Essentially, this signal power is the *stimulus-dependent* power in the neural response, i.e., the part of the response that is, in principle, predictable from the stimulus alone. The signal power therefore provides an absolute yardstick against which the performance of any stimulus-response function model can be judged. If the model is correct, then it should predict all of the signal power in the neural responses for a given stimulus, regardless of the level of noise power.

Upper and lower estimates of model predictive power

The estimate of the signal power forms a reference against which to compare the magnitude of response power accurately predicted by a particular model. This model *predictive power* is not necessarily the power of the predicted response $\boldsymbol{\rho}$, since that prediction may be inaccurate. Instead, as in the numerator of the coefficient of determination, it is given by the difference between the power in the observed response $P(\mathbf{r})$ and the *error power* or power in the residuals $P(\mathbf{r} - \boldsymbol{\rho})$.

The magnitude of this predictive power will depend both on the parameters used for the model prediction, and on the stimulus used to compare prediction to measurement. We define the *true predictive power* of a particular class of model (such as the STRF model) to be the predictive power that would be achieved by the version of the model with "ideal" parameters (e.g., ideal STRF weights or coefficients), which maximise predictive power across all stimulus-response combinations of the type under study (e.g., responses to all possible random chord stimuli). This true predictive power cannot be determined from realistic volumes of experimental data; however, it is possible to obtain a pair of predictive power estimates that are likely to bracket its value, as explained below.

Model parameters (such as the weights or coefficients of the STRF) are commonly estimated by minimising

the mean squared error of the model prediction on the training data. By definition, these least-mean-squares (LMS) parameters produce model predictions for the training data that have minimum possible error, and therefore maximal predictive power. Of course, the resulting maximal value, the *training predictive power*, will inevitably include an element of overfitting to the training data, and so will overestimate the true predictive power of the model with ideal parameters (which would perform best on average for all possible stimulus-response combinations, not just the training data). More precisely, the expected value of the training predictive power of the LMS parameters is an upper bound on the predictive power of the model with ideal parameters. Thus, the measured training predictive power can be considered an *upper estimate* of the true predictive power of the model class.

We can also obtain a *lower estimate*, defined similarly, by empirically measuring the generalization performance of the model by cross-validation. Cross-validation is a standard statistical procedure (Duda and Hart, 1973), in which each data set is repeatedly divided into a "training" segment and a "test" segment (in this study, 9/10 and 1/10 of the full stimulus length, respectively). Model parameters are estimated using responses to the training segment alone; a test prediction is obtained by applying the model to the test segment; and the mean squared difference between this prediction and the observed response to the test segment is calculated. This procedure is repeated multiple times (here, 10 times), on each occasion using a different division of the data into training and test segments. The average of the multiple mean-squared-error figures obtained in this way is the *cross-validation error power*. The difference between this error power and the total response power in the recording is the *cross-validation predictive power*. Cross-validation provides an unbiased estimate of the average generalization performance of the fitted models (as obtained from the training fraction of the available data). Since these models are inevitably overfit to their training data, not the test data, the expected value of this cross-validation predictive power bounds the predictive power of the model with ideal parameters from below, and thereby provides the desired lower estimate of the true predictive power of the model class. These lower estimates may be tightened somewhat by optimising model parameters to improve generalisation performance, for example using the Bayesian smoothing and de-noising techniques applied here (Sahani and Linden, 2003a).

Population extrapolation to zero-noise limit

For any one recording of finite length, the true predictive power of the model class (i.e., the predictive power of the version of the model with ideal parameters) can only be bracketed between the upper and lower estimates defined above. The looseness of these estimates will depend on the variability or noise in the recording. For a recording with high trial-to-trial variability, the model parameters will be more strongly overfit to the noise in the training data. Thus we expect the training predictive power on such a recording to appear high relative to the signal power, and the cross-validation predictive power to appear low. Indeed, in very high-noise conditions, the model may primarily describe the stimulus-independent noisy part of the training data, and so the training predictive power might exceed the estimated signal power ($\hat{P}(\boldsymbol{\mu})$), while the cross-validation predictive power may fall below zero (that is, the predictions made by the model may be worse than a simple unchanging mean rate prediction). Thus, the estimates may not usefully constrain the predictive power measure for a particular recording.

However, for a population of recorded neurons that are relatively homogeneous, it is possible to tighten the estimates of model predictive power *for the population as a whole*, by normalising the upper and lower estimates of model predictive power by the signal power for each recording, plotting these normalised estimates as a function of noise power—also normalised by signal power—for each recording, and then extrapolating across the population to the theoretical zero noise level. *The upper and lower estimates of model predictive power in this zero-noise limit provide the desired noise-independent measure of model predictive performance.* This extrapolation is shown in Fig. 2 of the main text for both STRF and CGF models, and for the populations of auditory cortical and thalamic recordings.

4 Fitting 1-D and 2-D quadratic models (related to Supplementary Fig. S3)

We implemented one- and two-dimensional quadratic models to compare with the CGF model. Like the CGF model, these models are constrained parametrisations of the second-order spectrotemporal Volterra kernel; however, the low-dimensional constraint is not formulated in terms of input-specific contextual gain. Similar models were discussed by Park et al. (2013) in the context of an approximate fitting procedure. While the

estimation method used there was consistent for low-rank models (in the sense that if the data actually arose from a low-rank quadratic model, their approach would converge to the correct parameters as the number of available data grew), the leading eigenvectors of the full second-order term do not estimate the optimal low-rank model when the data arise from a different process. Thus, to provide the fairest comparison to our CGF model fits, we explicitly sought low-rank quadratic forms which were optimal in the sense of regularised least-squares.

A K-dimensional quadratic model takes the form

$$\hat{r}(i) = c + \mathbf{w}^{\mathbf{tf}} \cdot \mathbf{s}(i) + \sum_{k=1}^{K} \lambda_k \big(\mathbf{w}_k^{\mathbf{q}} \cdot \mathbf{s}(i)\big)^2$$

where the vectors $\mathbf{w}_k^{\mathbf{q}}$ ($k = 1 \dots K$) parametrise the second-order Volterra kernel matrix (V) using K outer products: $V = \sum_{k=1}^{K} \lambda_k \mathbf{w}_k^{\mathbf{q}} \mathbf{w}_k^{\mathbf{q}\mathsf{T}}$. Including the dimension corresponding to the linear term $\mathbf{w}^{\mathbf{tf}}$, this model may also be interpreted as a $(K+1)$-dimensional LN cascade with a second-order polynomial nonlinearity.

A 1-D quadratic model has a similar number of degrees of freedom to the context model (a single quadratic basis component $\mathbf{w}_1^{\mathbf{q}}$ with 720 degrees of freedom in place of the CGF with 324 degrees of freedom). However least-squares fitting of such a model is not straightforward. Thus we first fit a 2-D quadratic model of the form

$$\hat{r}(i) = c + \mathbf{w}^{\mathbf{tf}} \cdot \mathbf{s}(i) + \mathbf{s}(i)^{\mathsf{T}} \mathbf{u} \mathbf{v}^{\mathsf{T}} \mathbf{s}(i)$$

using the same alternating least-squares method as we used to fit the CGF: alternately obtaining least-squares values of $(c, \mathbf{w}^{\mathbf{tf}}, \mathbf{u})$ holding $\mathbf{v}$ fixed, and of $(c, \mathbf{w}^{\mathbf{tf}}, \mathbf{v})$ holding $\mathbf{u}$ fixed. This approach also allowed us to use a regularising prior to improve generalisation — when fitting models for cross-validation we set the prior on $\mathbf{w}^{\mathbf{tf}}$, $\mathbf{u}$ and $\mathbf{v}$ to be the optimal ASD smoothing prior obtained when fitting the STRF model. The least-squares models used to evaluate training fit were unregularised.

Although it exploits a rank 1 decomposition of the quadratic kernel matrix, as long as $\mathbf{u}$ and $\mathbf{v}$ are unconstrained, this model is equivalent to a *two*-dimensional model with λ_k and $\mathbf{w}_k^{\mathbf{q}}$ ($k = 1, 2$) given by the eigenvalues and eigenvectors of $\frac{1}{2}(\mathbf{u}\mathbf{v}^{\mathsf{T}} + \mathbf{v}\mathbf{u}^{\mathsf{T}})$. (The equivalence follows from the observation that $\mathbf{s}^{\mathsf{T}} A \mathbf{s} = 0$ for an antisymmetric matrix A and any vector $\mathbf{s}$, and so only the symmetric part of the product $\mathbf{u}\mathbf{v}^{\mathsf{T}}$ contributes to the model output.) We found the 1-D quadratic model by gradient descent in the mean squared error, constraining $\mathbf{w}_1^{\mathbf{q}}$ to lie within the two-dimensional subspace spanned by the eigenvectors derived from the optimal (regularised or not, as appropriate) 2-D model. Although when using a general structured or natural-sound stimulus, the optimal 1-D quadratic model may not lie within the subspace spanned by the optimal 2-D model, the expected difference vanishes for a independent random stimulus with Gaussian-distributed amplitudes. Numerical experiments suggested that any bias introduced by our two-step estimation process was also small for the independent DRC stimulus.

5 Predicting the STRF for a DRC stimulus from the PRF and CGF (related to Supplementary Fig. S4)

The details of an STRF fit to a nonlinear neural response will depend on details of the stimulus by which the response was evoked. Stimuli with non-trivial statistical structure — such as natural sounds and some artificial stimuli including spectrotemporal ripples — may engage specific nonlinear encoding mechanisms and lead to STRF estimates that substantially misrepresent the neuron's true response properties (Christianson et al., 2008). This general point will also apply to responses with nonlinear context-dependent input-specific gain modulation of the type revealed here, and so an STRF estimated by neglecting contextual effects may differ significantly from the corresponding PRF in ways that reflect the interaction between the context-dependence and the structured statistics of the sound.

However, the DRC stimulus, with its independent and identically distributed (iid) tone pulses, is designed to reduce the effects of such nonlinear distortion (Christianson et al., 2008). In particular, this property means that — provided the dominant combination-dependent nonlinearity in the response is indeed contextual input-specific gain modulation — it is possible to find a closed-form expression for the STRF weights that should be estimated from a DRC stimulus by using the estimated values of the PRF and CGF.

Consider a DRC stimulus with iid pulse energies $s(i, k)$ (where i indexes time and k pulse frequency) that evokes

a measured response $r(i)$ in a neuron whose mean firing rate is accurately described by the quadratic contextual input-specific gain model. Then,

$$r(i) = c + \sum_{j=0}^{J} \sum_{k=1}^{K} w^{\mathbf{tf}}_{j+1,k} s(i-j,k) \left(1 + \sum_{m=0}^{M} \sum_{n=-N}^{N} w^{\boldsymbol{\tau\phi}}_{m+1,n+N+1} s(i-j-m, k+n) \right) + \eta(i) \,, \tag{S3}$$

where c is a firing rate offset, $w^{\mathbf{tf}}_{\cdot,\cdot}$ are the PRF weights, $w^{\boldsymbol{\tau\phi}}_{\cdot,\cdot}$ the CGF weights, and $\eta(i)$ is a noise term with zero mean but otherwise unconstrained distribution. It will be useful to collect the PRF weights into a vector with $L \equiv (J+1)K$ elements, $\mathbf{w^{tf}}$. The subscript notation $\mathbf{w}^{\mathbf{tf}}_{(jk)}$ will then refer to the element of the PRF vector that corresponds to time offset j and frequency bin k in the PRF matrix: that is, $\mathbf{w}^{\mathbf{tf}}_{(jk)} = w^{\mathbf{tf}}_{j+1,k}$. Similarly, we define an L-element stimulus vector $\mathbf{s}(i)$ such that $\mathbf{s}_{(jk)}(i) = s(i-j,k)$; and also an $(L+1)$-element augmented stimulus vector $\tilde{\mathbf{s}}(i) = \begin{bmatrix} 1 \\ \mathbf{s}(i) \end{bmatrix}$

Now consider the estimate of an STRF defined over the same $(J+1) \times K$ region of the stimulus as spanned by the PRF. The STRF model is

$$\hat{r}(i) = c^{\mathrm{STRF}} + \sum_{j=0}^{J} \sum_{k=1}^{K} w^{\mathrm{STRF}}_{j+1,k} s(i-j,k) \,, \tag{S4}$$

where c^{STRF} is model firing rate offset (which might differ from c) and $w^{\mathrm{STRF}}_{\cdot,\cdot}$ are the STRF weights. Again, we define an L-element vector $\mathbf{w^{STRF}}$ with $\mathbf{w}^{\mathbf{STRF}}_{(jk)} = w^{\mathrm{STRF}}_{j+1,k}$, and the $(L+1)$-element vector $\widetilde{\mathbf{w}}^{\mathbf{STRF}} = \begin{bmatrix} c^{\mathrm{STRF}} \\ \mathbf{w^{STRF}} \end{bmatrix}$. Then the least-squares estimate of the STRF parameters is given by the familiar regression form:

$$\widetilde{\mathbf{w}}^{\mathbf{STRF}} = \left\langle \tilde{\mathbf{s}}\tilde{\mathbf{s}}^{\mathsf{T}} \right\rangle^{-1} \langle r \tilde{\mathbf{s}} \rangle \,. \tag{S5}$$

The angle brackets in equation S5 represent averages over time and we drop the explicit time index i from the averaged expressions. We assume that the stimulus used for estimation is long enough for these time-averages to converge to their corresponding expected values (an assumption that also justifies the use of the unregularised maximum-likelihood estimate). The expected value of the estimated STRF for a neuron with a mean firing rate described by the contextual input-specific gain model is then obtained by evaluating the expectations of equation S5 with r set to the value given by equation S3. We perform this evaluation one term at a time.

Consider first the stimulus autocorrelation term $\left\langle \tilde{\mathbf{s}}\tilde{\mathbf{s}}^{\mathsf{T}} \right\rangle^{-1}$. As the tone pulses within the DRC stimulus are iid, the expected value and variance of each stimulus element have constant values, which we write as $\bar{s}$ and σ_s^2 respectively. The expected second moment of each stimulus element is then $\left\langle s(i-j,k)^2 \right\rangle = \bar{s}^2 + \sigma_s^2$; but by independence the cross-moments are just $\langle s(i-j,k) s(i-j',k') \rangle = \bar{s}^2$ when $j \neq j'$ or $k \neq k'$. Assembling these values into matrix form (and writing $\mathbf{1}$ for a vector of L ones and I for the $L \times L$ identity matrix):

$$\left\langle \tilde{\mathbf{s}}\tilde{\mathbf{s}}^{\mathsf{T}} \right\rangle^{-1} = \left\langle \begin{bmatrix} 1 \\ \mathbf{s} \end{bmatrix} \begin{bmatrix} 1 & \mathbf{s}^{\mathsf{T}} \end{bmatrix} \right\rangle^{-1} = \begin{bmatrix} 1 & \langle \mathbf{s} \rangle^{\mathsf{T}} \\ \langle \mathbf{s} \rangle & \left\langle \mathbf{s}\mathbf{s}^{\mathsf{T}} \right\rangle \end{bmatrix}^{-1} = \begin{bmatrix} 1 & \bar{s}\mathbf{1}^{\mathsf{T}} \\ \bar{s}\mathbf{1} & \bar{s}^2 \mathbf{1}\mathbf{1}^{\mathsf{T}} + \sigma_s^2 I \end{bmatrix}^{-1} \tag{S6}$$

The inverse follows from the block-matrix identity:

$$\begin{bmatrix} A & B \\ C & D \end{bmatrix}^{-1} = \begin{bmatrix} A^{-1} + A^{-1} B S_{|A}^{-1} C A^{-1} & -A^{-1} B S_{|A}^{-1} \\ -S_{|A}^{-1} C A^{-1} & S_{|A}^{-1} \end{bmatrix} \tag{S7}$$

where $S_{|A} = D - CA^{-1}B$ is the Schur complement of the block A. For the current matrix

$$S_{|A} = \bar{s}^2 \mathbf{1}\mathbf{1}^{\mathsf{T}} + \sigma_s^2 I - \bar{s}\mathbf{1} \cdot 1 \cdot \mathbf{1}^{\mathsf{T}} \bar{s} = \sigma_s^2 I \tag{S8}$$

so

$$\left\langle \tilde{\mathbf{s}}\tilde{\mathbf{s}}^{\mathsf{T}} \right\rangle^{-1} = \begin{bmatrix} 1 + \sigma_s^{-2} \bar{s} \mathbf{1}^{\mathsf{T}} \mathbf{1} \bar{s} & -\sigma_s^{-2} \bar{s} \mathbf{1}^{\mathsf{T}} \\ -\sigma_s^{-2} \bar{s} \mathbf{1} & \sigma_s^{-2} I \end{bmatrix} = \sigma_s^{-2} \begin{bmatrix} \sigma_s^2 + L\bar{s}^2 & -\bar{s}\mathbf{1}^{\mathsf{T}} \\ -\bar{s}\mathbf{1} & I \end{bmatrix} . \tag{S9}$$

Turning now to the correlation term $\langle r\tilde{\mathbf{s}}\rangle$, we note that as the first element of $\tilde{\mathbf{s}}$ is always 1 we have

$$\langle r\tilde{\mathbf{s}}\rangle_1 = \langle r\rangle = c + \sum_{j=0}^{J}\sum_{k=1}^{K} w^{\mathbf{tf}}_{j+1,k}\bar{s}\left(1 + \sum_{m=0}^{M}\sum_{n=-N}^{N} w^{\boldsymbol{\tau\phi}}_{m+1,n+N+1}\bar{s}\right) + \langle\eta\rangle\,, \tag{S10}$$

where we have used the iid property of the stimulus and the fact that the input gain term does not depend on $s(i-j,k)$. We define $\mathcal{W}_{\text{PRF}} \equiv \sum_{j=0}^{J}\sum_{k=1}^{K} w^{\mathbf{tf}}_{j+1,k}$ and $\mathcal{W}_{\text{CGF}} \equiv \sum_{m=0}^{M}\sum_{n=-N}^{N} w^{\boldsymbol{\tau\phi}}_{m+1,n+N+1}$ and note that the noise has zero mean, giving

$$\langle r\tilde{s}_1\rangle = c + \mathcal{W}_{\text{PRF}}\bar{s} + \mathcal{W}_{\text{PRF}}\mathcal{W}_{\text{CGF}}\bar{s}^2 \equiv \alpha\,, \tag{S11}$$

where the definition of α will be valuable below.

The correlation with the (pq)th element of the stimulus vector is given by

$$\left\langle r\mathbf{s}_{(pq)}\right\rangle = \left\langle \mathbf{s}_{(pq)}\left[c + \sum_{j=0}^{J}\sum_{k=1}^{K} w^{\mathbf{tf}}_{j+1,k}\mathbf{s}_{(jk)}\left(1 + \sum_{m=0}^{M}\sum_{n=-N}^{N} w^{\boldsymbol{\tau\phi}}_{m+1,n+N+1}\mathbf{s}_{(j+m,k+n)}\right)\right]\right\rangle \tag{S12}$$

$$= c\bar{s} + \sum_{j=0}^{J}\sum_{k=1}^{K} w^{\mathbf{tf}}_{j+1,k}\left\langle\mathbf{s}_{(pq)}\mathbf{s}_{(jk)}\right\rangle + \sum_{j=0}^{J}\sum_{k=1}^{K}\sum_{m=0}^{M}\sum_{n=-N}^{N} w^{\mathbf{tf}}_{j+1,k}w^{\boldsymbol{\tau\phi}}_{m+1,n+N+1}\left\langle\mathbf{s}_{(pq)}\mathbf{s}_{(jk)}\mathbf{s}_{(j+m,k+n)}\right\rangle\,. \tag{S13}$$

Now,

$$\left\langle\mathbf{s}_{(pq)}\mathbf{s}_{(jk)}\right\rangle = \begin{cases}\bar{s}^2 + \sigma_s^2 & \text{if } (pq) = (jk)\\ \bar{s}^2 & \text{otherwise}\,,\end{cases} \tag{S14}$$

and

$$\left\langle\mathbf{s}_{(pq)}\mathbf{s}_{(jk)}\mathbf{s}_{(j+m,k+n)}\right\rangle = \begin{cases}\bar{s}^3 + \sigma_s^2\bar{s} & \text{if } (pq) = (jk) \text{ or } (pq) = (j+m,k+n)\\ \bar{s}^3 & \text{otherwise}\,,\end{cases} \tag{S15}$$

and the case $(jk) = (j+m,k+n)$ does not contribute as the corresponding CGF weight is set to 0. Thus,

$$\left\langle r\mathbf{s}_{(pq)}\right\rangle = c\bar{s} + \bar{s}^2\mathcal{W}_{\text{PRF}} + \sigma_s^2\mathbf{w}^{\mathbf{tf}}_{(pq)} + \bar{s}^3\mathcal{W}_{\text{PRF}}\mathcal{W}_{\text{CGF}} + \sigma_s^2\bar{s}\mathcal{W}_{\text{CGF}}\mathbf{w}^{\mathbf{tf}}_{(pq)} + \sigma_s^2\bar{s}\sum_{j=0}^{p-1}\sum_{k=\max(1,q-N)}^{\min(K,q+N)} w^{\mathbf{tf}}_{j+1,k}w^{\boldsymbol{\tau\phi}}_{p-j+1,q-k+N+1}\,. \tag{S16}$$

Now recall the definition of α from equation S11, and further define $\beta \equiv (1 + \bar{s}\mathcal{W}_{\text{CGF}})$ as well as $\mathbf{w}^{\mathbf{conv}}_{(pq)} \equiv \sum_{jk} w^{\mathbf{tf}}_{j+1,k}w^{\boldsymbol{\tau\phi}}_{p-j+1,q-k+N+1}$ with limits as in equation S16, so that $\mathbf{w}^{\mathbf{conv}}$ is the vector representing the $(J+1)\times K$ region of the 2D convolution between the CGF and PRF that is central in frequency and causal in time. We can then write:

$$\left\langle r\mathbf{s}_{(pq)}\right\rangle = \bar{s}\alpha + \sigma_s^2\beta\mathbf{w}^{\mathbf{tf}}_{(pq)} + \sigma_s^2\bar{s}\mathbf{w}^{\mathbf{conv}}_{(pq)}\,. \tag{S17}$$

Finally, combining equations S9, S11 and S17, we have

$$\widetilde{\mathbf{w}}^{\mathbf{STRF}} = \left\langle\tilde{\mathbf{s}}\tilde{\mathbf{s}}^{\mathsf{T}}\right\rangle^{-1}\langle r\tilde{\mathbf{s}}\rangle = \sigma_s^{-2}\begin{bmatrix}\sigma_s^2 + L\bar{s}^2 & -\bar{s}\mathbf{1}^{\mathsf{T}}\\ -\bar{s}\mathbf{1} & I\end{bmatrix}\begin{bmatrix}\alpha\\ \bar{s}\alpha\mathbf{1} + \sigma_s^2\beta\mathbf{w}^{\mathbf{tf}} + \sigma_s^2\bar{s}\mathbf{w}^{\mathbf{conv}}\end{bmatrix}\,, \tag{S18}$$

which, with some simplification and setting $\mathcal{W}_{\text{conv}} \equiv \sum_{(pq)}\mathbf{w}^{\mathbf{conv}}_{(pq)}$, yields:

$$\widetilde{\mathbf{w}}^{\mathbf{STRF}} = \begin{bmatrix}c - \bar{s}^2\mathcal{W}_{\text{conv}}\\ \beta\mathbf{w}^{\mathbf{tf}} + \bar{s}\mathbf{w}^{\mathbf{conv}}\end{bmatrix}\,. \tag{S19}$$

Thus, the expected weights of the STRF correspond to the weights of the PRF scaled by the factor β and modified by the convolutional factor $\bar{s}\mathbf{w}^{\mathbf{conv}}$.

The accuracy of this prediction is shown in Supplementary Fig. S4.

Supplementary References

Ahrens, M. B., Paninski, L., and Sahani, M. (2008). Inferring input nonlinearities in neural encoding models. *Network*, 19(1):35–67.

Anderson, L. A., Christianson, G. B., and Linden, J. F. (2009). Mouse auditory cortex differs from visual and somatosensory cortices in the laminar distribution of cytochrome oxidase and acetylcholinesterase. *Brain Res.*, 1252:130–142.

Anderson, L. A. and Linden, J. F. (2011). Physiological differences between histologically defined subdivisions in the mouse auditory thalamus. *Hear. Res.*, 274(1-2):48–60.

Christianson, G. B., Sahani, M., and Linden, J. F. (2008). The consequences of response nonlinearities for interpretation of spectrotemporal receptive fields. *J. Neurosci.*, 28(2):446–455.

Duda, R. O. and Hart, P. E. (1973). *Pattern Classification and Scene Analysis.* Oxford University Press.

Lewicki, M. S. (1998). A review of methods for spike sorting: the detection and classification of neural action potentials. *Network*, 9(4):53–78.

Linden, J. F., Liu, R. C., Sahani, M., Schreiner, C. E., and Merzenich, M. M. (2003). Spectrotemporal structure of receptive fields in areas AI and AAF of mouse auditory cortex. *J. Neurophysiol.*, 90(4):2660–2675.

Park, I., Archer, E., Priebe, N. J., and Pillow, J. W. (2013). Spectral methods for neural characterization using generalized quadratic models. *Advances in Neural Information Processing Systems*, 26:2454–2462.

Sahani, M. (1999). *Latent variable models for neural data analysis.* PhD thesis, California Institue of Technology.

Sahani, M. and Linden, J. F. (2003a). Evidence optimization techniques for estimating stimulus-response functions. In Becker, S., Thrun, S., and Obermayer, K., editors, *Advances in neural information processing systems*, pages 301–308. MIT Press, Cambridge, MA.

Sahani, M. and Linden, J. F. (2003b). How linear are auditory cortical responses? In Becker, S., Thrun, S., and Obermayer, K., editors, *Advances in neural information processing systems*, pages 109–116. MIT Press, Cambridge, MA.

Smith, P. H. and Populin, L. C. (2001). Fundamental differences between the thalamocortical recipient layers of the cat auditory and visual cortices. *J. Comp. Neurol.*, 436(4):508–519.
