# Supplementary material for: Input-Specific Gain Modulation by Local Sensory Context Shapes Cortical and Thalamic Responses to Complex Sounds
Source: Neuron. 2016 Jul 20;91(2):467–81. doi: 10.1016/j.neuron.2016.05.041 (PMC4961224; doi:10.1016/j.neuron.2016.05.041)
Supplement: Document S2. Article plus Supplemental Information [file mmc2.pdf]

Neuron

Article

Input-Specific Gain Modulation by Local Sensory Context Shapes Cortical and Thalamic Responses to Complex Sounds

Highlights

- Gain of neuronal responses to sound components varies with immediate acoustic context
- "Contextual gain fields" can be estimated from neuronal responses to complex sounds
- Coincident sound at different frequencies boosts gain in cortex and thalamus
- Preceding sound at similar frequency reduces gain for longer in cortex than thalamus

Authors

Ross S. Williamson, Misha B. Ahrens, Jennifer F. Linden, Maneesh Sahani

Correspondence

j.linden@ucl.ac.uk (J.F.L.),
maneesh@gatsby.ucl.ac.uk (M.S.)

In Brief

Williamson et al. (2016) show how encoding of individual components within complex sounds depends on the immediate acoustic neighborhood surrounding each component. These findings challenge the model that nonlinearity only follows integration, highlighting instead fine-grained nonlinear interactions within the receptive field.

CrossMark
Williamson et al., 2016, Neuron *91*, 467–481
July 20, 2016 © 2016 The Authors. Published by Elsevier Inc.
http://dx.doi.org/10.1016/j.neuron.2016.05.041

CellPress

CellPress

Input-Specific Gain Modulation by Local Sensory Context Shapes Cortical and Thalamic Responses to Complex Sounds

Ross S. Williamson,[1,2,7] Misha B. Ahrens,[3,4,8] Jennifer F. Linden,[5,6,9,*] and Maneesh Sahani[1,9,*]
[1]Gatsby Computational Neuroscience Unit, University College London, London W1T 4JG, UK
[2]Centre for Mathematics and Physics in the Life Sciences and Experimental Biology, University College London, London WC1E 6BT, UK
[3]Department of Molecular and Cellular Biology, Harvard University, Cambridge, MA 02138, USA
[4]Computational and Biological Learning Lab, Department of Engineering, University of Cambridge, Cambridge CB2 1PZ, UK
[5]Ear Institute, University College London, London WC1X 8EE, UK
[6]Department of Neuroscience, Physiology and Pharmacology, University College London, London WC1E 6BT, UK
[7]Present address: Eaton-Peabody Laboratories, Massachusetts Eye and Ear Infirmary, Boston, MA 02114, USA; Centre for Computational Neuroscience and Neural Technology, Boston University, Boston, MA 02115, USA
[8]Present address: Janelia Research Campus, Howard Hughes Medical Institute, Ashburn, VA 20147, USA
[9]Co-senior author
*Correspondence: j.linden@ucl.ac.uk (J.F.L.), maneesh@gatsby.ucl.ac.uk (M.S.)
http://dx.doi.org/10.1016/j.neuron.2016.05.041

SUMMARY

Sensory neurons are customarily characterized by one or more linearly weighted receptive fields describing sensitivity in sensory space and time. We show that in auditory cortical and thalamic neurons, the weight of each receptive field element depends on the pattern of sound falling within a local neighborhood surrounding it in time and frequency. Accounting for this change in effective receptive field with spectrotemporal context improves predictions of both cortical and thalamic responses to stationary complex sounds. Although context dependence varies among neurons and across brain areas, there are strong shared qualitative characteristics. In a spectrotemporally rich soundscape, sound elements modulate neuronal responsiveness more effectively when they coincide with sounds at other frequencies, and less effectively when they are preceded by sounds at similar frequencies. This local-context-driven lability in the representation of complex sounds—a modulation of "input-specific gain" rather than "output gain"—may be a widespread motif in sensory processing.

INTRODUCTION

For decades, the linearly weighted receptive field has been used to describe sensory neural responses to complex stimuli. Neurons in the central auditory system integrate sound over time and frequency, making the linear model of choice the spectrotemporal receptive field or STRF (e.g., Aertsen et al., 1980; Eggermont et al., 1983; Eggermont 1993; deCharms et al., 1998; Depireux et al., 2001). Features of the STRF have been used to investigate neural representations in different brain areas (Nelken et al., 1997; Miller et al., 2002; Escabi and Schreiner 2002; Linden et al., 2003; Woolley et al., 2005), and changes in the shape or overall gain of the STRF have been used to examine how auditory encoding varies with stimulus type (Gill et al., 2006), sound density (Blake and Merzenich 2002; Valentine and Eggermont 2004), spectrotemporal contrast (Rabinowitz et al., 2011; Rabinowitz et al., 2012), and behavioral task (Fritz et al., 2003, 2007; David et al., 2012). One or more STRF-like weighted fields also lie at the heart of linear-nonlinear (LN) cascades, including generalized linear point-process models (Chornoboy et al., 1988) and linear-nonlinear-Poisson models estimated by spike-triggered characterization, maximally informative dimensions and similar methods (Figures 1A–1C; for reviews, see Schwartz et al., 2006; Paninski et al., 2007; Sharpee, 2013).

Despite its wide use, the STRF is known to be an incomplete description of neural responses. Linear STRF predictions capture less than half of the reliable response variance to a complex stimulus in the primary auditory cortex, even without adaptive or task-dependent changes (Sahani and Linden, 2003; Machens et al., 2004). More fundamentally, the crucial assumption of linear weighting—that the sensitivity of the neuron to a local element of the stimulus is independent of the rest of the stimulus—is challenged by many reports of nonlinear combination sensitivity. Such nonlinearities include "forward suppression" of the response to the second tone in a pair (Brosch and Schreiner, 1997; Wehr and Zador, 2005), more complex combination effects for spectrally offset tone pairs (Kadia and Wang, 2003; Sadagopan and Wang, 2009), quadratic sensitivity to the distribution of spectral energy in random-spectrum noise (Yu and Young, 2000; Young and Calhoun, 2005), and nonlinear sensitivity to parts extracted from simple vocalizations (Bar-Yosef et al., 2002; Bar-Yosef and Nelken, 2007).

How do these, and perhaps other, nonlinearities combine over frequency and time to shape responses to complex sounds at different stages of auditory processing? Are time-frequency sensitivities modified substantially by these nonlinear local

This is an open access article under the CC BY license (http://creativecommons.org/licenses/by/4.0/).

CellPress

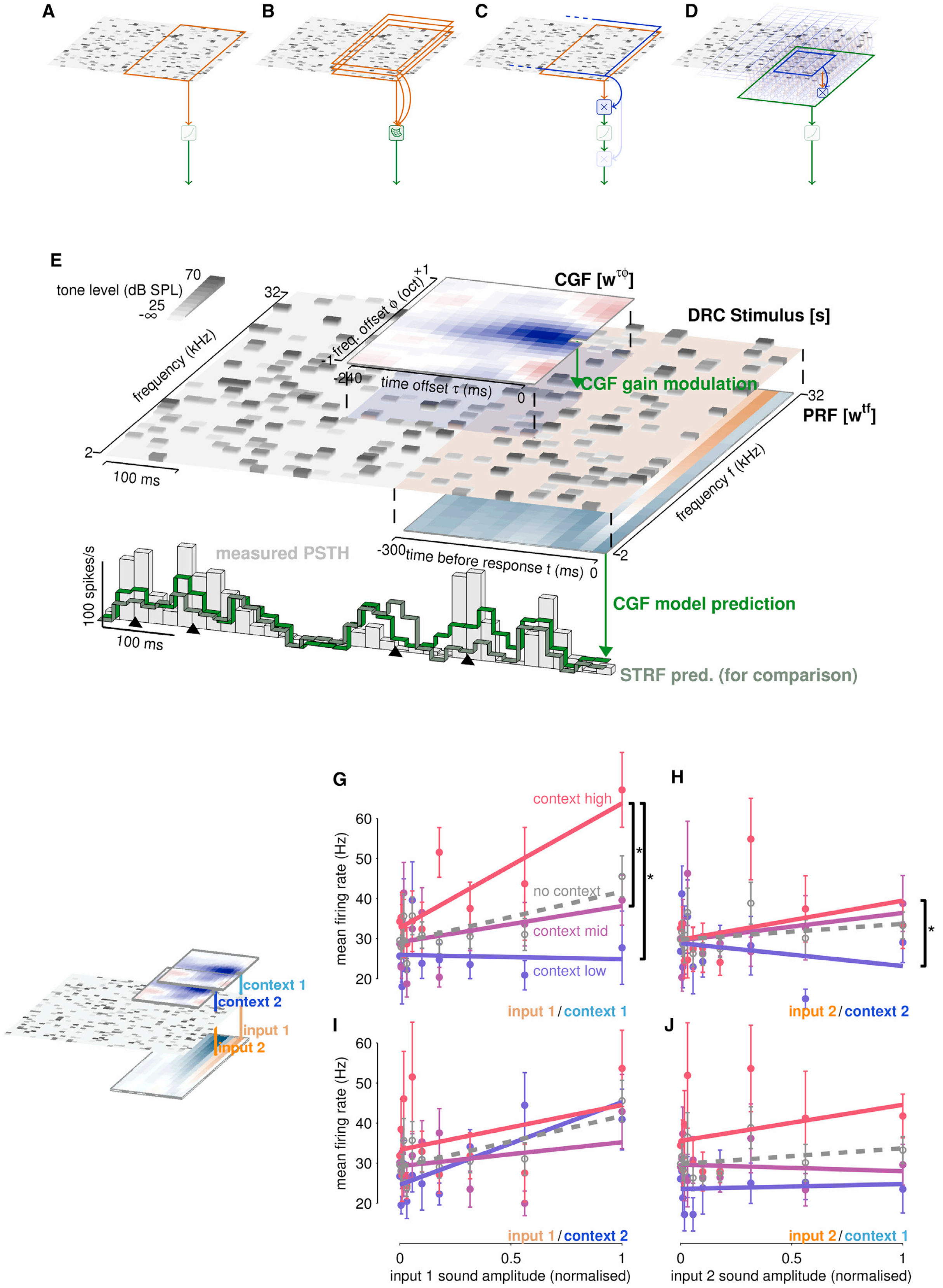

(legend on next page)

interactions? Or might the local contextual nonlinearities average away to leave a broadly linear response that is qualitatively, if not quantatively, well described by the STRF?

To address these questions, we extended the multilinear model of Ahrens et al. (2008a) to study the impact of local acoustic context on cortical and thalamic responses.

RESULTS

Modeling Local Contextual Input-Specific Gain

We modeled responses of neurons in the auditory cortex and thalamus to statistically stationary, spectrotemporally rich, dynamic random chord (DRC) stimuli using a multilinear approach in which local acoustic context could modify the sensitivity of the neuron to sound level (Figure 1D). The model, as we applied it, combines two matrices of weights. The first is an STRF-like principal receptive field (PRF; $\mathbf{w}^{\mathbf{tf}}$) with weights defined in absolute frequency and time-lag preceding the response. These weights represent the spectrotemporal sensitivities of the neuron in the absence of local contextual influences. In principle, they would correspond to responses evoked by brief isolated tones with no acoustic energy at nearby frequencies and times—although they were fit using responses to the rich DRC stimulus. These PRF sensitivities are multiplicatively modulated through the action of the second matrix, a contextual gain field (CGF; $\mathbf{w}^{\tau\phi}$) with weights defined in terms of *relative* offsets of time (τ) and frequency (ϕ). The CGF defines an acoustic neighborhood or local context around each time-frequency element or "tile" of the discretised stimulus spectrogram. The pattern of energy that falls within that neighborhood is weighted by the entries of the CGF and summed, and this term then multiplies the effect of the energy within the anchoring time-frequency tile on the neural response (Figure 1E), providing "input-specific gain."

Thus, for a sound with spectrotemporal energy at time t in frequency channel f given by $s(t,f)$, the modeled firing rate $\hat{r}$ at time i was expressed by the equation

$$\hat{r}(i) = c + \sum_{j=0}^{J} \sum_{k=1}^{K} w^{\mathbf{tf}}_{j+1,k} s(i-j,k) \times \left(1 + \sum_{m=0}^{M} \sum_{n=-N}^{N} w^{\tau\phi}_{m+1,n+N+1} s(i-j-m,k+n)\right), \quad (1)$$

where the constant c sets a baseline firing rate. The zero-offset CGF weight $w^{\tau\phi}_{1,N+1}$ (note the unconventional summation limits for the indices m and n) was fixed to 0 so that no time-frequency energy contributed to its own context, preserving a linear model response to isolated tones.

The CGF in this model sets a different context-dependent gain at each spectrotemporal tile of the stimulus. This input-specific gain enhances or suppresses the PRF-mediated effect of the stimulus but, provided that the term in parentheses in Equation 1 remains positive, maintains its sign. Thus, the sign of a CGF weight, unlike that of a PRF or STRF weight, does not directly indicate whether sound energy excites or inhibits the neuron. Instead, a positive CGF weight at a particular time-frequency offset indicates that if an input within the PRF were paired with energy only at this relative offset, then the gain with which the PRF-input influenced firing is boosted above 1; thus, for a positive PRF weight firing would be further enhanced, whereas for negative PRF weights, activity would be more suppressed. The obverse holds if the CGF weight is negative; gain is reduced and so the input within the PRF would drive less excitation if positive, and less inhibition if negative. In a complex stimulus,

Figure 1. Local Context Shapes Input-Specific Gain

(A–D) Cartoon illustrations of receptive field integration mechanisms.

(A) In the most basic scheme, input stimuli (gray-level spectrogram) are integrated by a single set of fixed weights (orange). Pointwise nonlinear transforms may apply to each specific input (not shown) or to the integrated weights (light green).

(B) Multidimensional LNP models include a small number of differently weighted overlapping integration fields, with outputs combined by a multi-imensional nonlinearity (green). Methods such as MID and STC are designed to characterize such models.

(C) Normalization, or other variable global gain, involves the output of one field (blue) modulating the gain of the integrated response to the other (orange). The normalization field may extend well beyond the integration field, so that the effective gain reflects global statistical properties of the stimulus. A further nonlinear transformation (light green) may act before or after gain modulation (light blue).

(D) In the phenomenon described here, local context (blue) around each input shapes the gain of response to that specific input. Each input experiences a different context and thus a potentially different gain. Gain-modulated inputs are integrated (green), with a possible further nonlinear transformation (light green).

(E) The CGF model. The contextual input-specific gain model incorporates two sets of time-freqency weights. The Principal Receptive Field (PRF; $\mathbf{w}^{\mathbf{tf}}$) describes the basic sensitivity of the neuron to spectrotemporal energy at all frequencies within a short time window, analogous to the STRF. The Contextual Gain Field (CGF; $\mathbf{w}^{\tau\phi}$) describes how each sensitivity is modified by its local acoustic context. The model can be viewed as acting in two stages. First, the stimulus spectrogram is convolved with the CGF in both time and frequency to estimate the local input-specific gain at each spectrotemporal point (upper green arrow). The local stimulus power is then scaled by the corresponding gain and these scaled values, weighted by the PRF, are summed to model the neural response (lower green arrow). The measured response (peri-stimulus-time histogram or PSTH) for one example cortical neuron is shown (gray bars) along with the rates predicted by the CGF model (bright green) and an unmodified STRF (dull gray-green). Differences in prediction (black *triangles*) show that local contextual gain effects both increase and decrease firing rates relative to the STRF model of static sensitivities.

(F–J) Local input-specificity of contextual gain effects. The relationships between the measured responses of one example unit and the sound level at two spectrotemporal locations within the unit's PRF far enough apart in time and frequency to be subject to different local sound contexts (F) are shown without reference to local context (gray open circles and dashed lines); sorted by whether the integrated contextual energy in a local window around that spectrotemporal location fell within a low, middle or high quantile (G and H, colored circles and lines); or, as a control, sorted according to "distant" contextual energy — i.e., integrated energy around the other of the two input locations (I and J, colored circles and lines). Error bars indicate standard error in the mean; lines are fit to the empirical data. The slopes of the input-response relationships differ when sorted by local spectrotemporal context (black bars with asterisks indicate significance), but not when sorted by contextual energy at the spectrotemporally distant location.

the influence of energy at all offsets around each input in the PRF is linearly combined through the CGF to yield a single gain for that specific input, and the gain-modulated inputs are linearly combined through the PRF to model the neuronal firing rate.

We fit the CGF model to DRC-evoked responses recorded extracellularly from neurons in the auditory cortex and thalamus of anaesthetised CBA/Ca mice. The final analysis database included 64 prolonged continuous recordings from auditory cortex and 101 from auditory thalamus. Cortical recordings corresponded to a subset of the DRC stimulus recordings previously used for STRF analysis by Linden et al. (2003); see Experimental Procedures for details.

Input Gain Is Specific to Local Context

We found that local context played a substantial role in shaping input-specific gain. To illustrate the effect, we chose two spectrotemporal positions within the responsive region of an example unit's STRF, separated by about an octave to minimize overlap in local context (Figure 1F). Plots of the average response as a function of the sound level at each of these two positions revealed roughly linear relationships, the (positive) slopes of which were essentially unregularised estimates of the corresponding (excitatory) STRF weights (Figure 1G–1J, "no context," gray). We then asked whether the slope of this relationship at each time and frequency could be modulated by acoustic context either immediately surrounding the specific chosen time-frequency input or distant from it. We calculated, moment by moment, the integrated sound energy within a local window surrounding each of the chosen time-frequency points, weighted using a CGF estimated by a cross-validation procedure (see Supplemental Experimental Procedures 2). When the integrated energy at position 1 was within the bottom third of its range ("context low," blue) the slope of the stimulus-response relationship fell to almost 0; if in the middle third ("context mid," magenta) the slope was roughly the same as when context was ignored; and if in the highest third ("context high," red) the gain was boosted substantially (Figure 1G) and significantly (permutation tests: low to mid, $p = 0.20$; mid to high, $p = 0.027$; low to high, $p = 0.0030$). The same trend was evident in the relationships between the response and the sound level at position 2, when grouped by the integrated contextual energy at position 2 (Figure 1H; low to mid, $p = 0.073$; mid to high, $p = 0.38$; low to high, $p = 0.041$). However, the slope of the response to sound level at input 1 did not vary with the context at position 2 (Figure 1I; low to mid, $p = 0.90$; mid to high, $p = 0.35$; low to high, $p = 0.81$), nor vice versa (Figure 1J; low to mid, $p = 0.63$; mid to high, $p = 0.12$; low to high, $p = 0.21$).

Thus, only local, not distant, acoustic context affected the gain with which a specific time-frequency input drove firing. This observation is inconsistent with a single STRF-like integration field followed by a static output nonlinearity (Figure 1A) or modulated by a single global gain factor (Figure 1C). It also argues against the sufficiency of a low-dimensional LN model (Figure 1B), as the input-specific context could only be captured by a separate linear filter around each input. However it does not necessarily require that each local context filter is a translated copy of the same CGF weights. This assumed structure (Equation 1) was tested by explicit comparison to alternative nonlinear models described later.

Contextual Input-Specific Gain Shapes Cortical and Thalamic Responses

Before evaluating the CGF model against nonlinear alternatives, we measured the contribution of contextual input-specific gain modulation to neuronal output by quantifying predictive accuracy relative to the linear STRF model. In doing so, it was necessary to rule out the possibility that any improved prediction came from "overfitting" of the additional parameters of the CGF. We used two approaches.

First, we compared the generalization performance of the CGF and STRF models in individual neurons, cross-validating by repeatedly fitting each model to one section of response ("training data") and evaluating performance on another ("test data"). The added CGF parameters always enable an apparently better fit to the training data. However, if local context were unimportant, then improvement would come only from overfitting to random fluctuations, and would not extend to the unrelated fluctuations of the test data. Indeed, the overfit model parameters would generate perturbed predictions, lowering cross-validation accuracy below that of the STRF. In fact, we found the opposite: the CGF model outperformed the linear STRF model in cross-validation for almost every neuron (Figure 2A), suggesting that local contextual modulation of input-specific gain does indeed reliably shape responses to complex sounds.

Second, we followed Sahani and Linden (2003) to obtain population-level predictive performance estimates for both models. When expressed as a proportion of the estimated stimulus-dependent signal power (see Supplemental Experimental Procedures 3), performance on both training data and test data—assessed by cross-validation—depended systematically on the amount of variability or "noise" in the recording (Figures 2B and 2C). Each of these relationships could be extrapolated to yield "zero-noise" predictive power limits, effectively averaging across the population while discounting the variable impact of noise on each unit. On the training data, the extrapolated value eliminates contributions from overfitting to random fluctuations but may still reflect overfitting to the details of the particular stimulus segment used for training. The equivalent value on test data also minimizes the impact of random fluctuations on model fits but retains any generalization penalty resulting from estimation of the model parameters from finite data. Thus, the two extrapolated limits bracket the true average predictive power of the model class.

Both training and test extrapolated values were consistently higher for the CGF model than for a linear STRF model ([test, training] values were as follows: cortex CGF = [0.37, 0.79], STRF = [0.31, 0.51]; thalamus CGF = [0.52, 0.83], STRF = [0.48, 0.68]; see Figures 2B and 2C). Taking the midpoints of these ranges, we find that modeling the variation in contextual input-specific gain provides a 41% boost in predictive power over the linear STRF relationship in cortex, and a 16% boost in thalamus.

CGF Model Outperforms Related Second-Order Models

In designing the CGF model to capture the phenomenon of contextual input-specific gain modulation as simply and

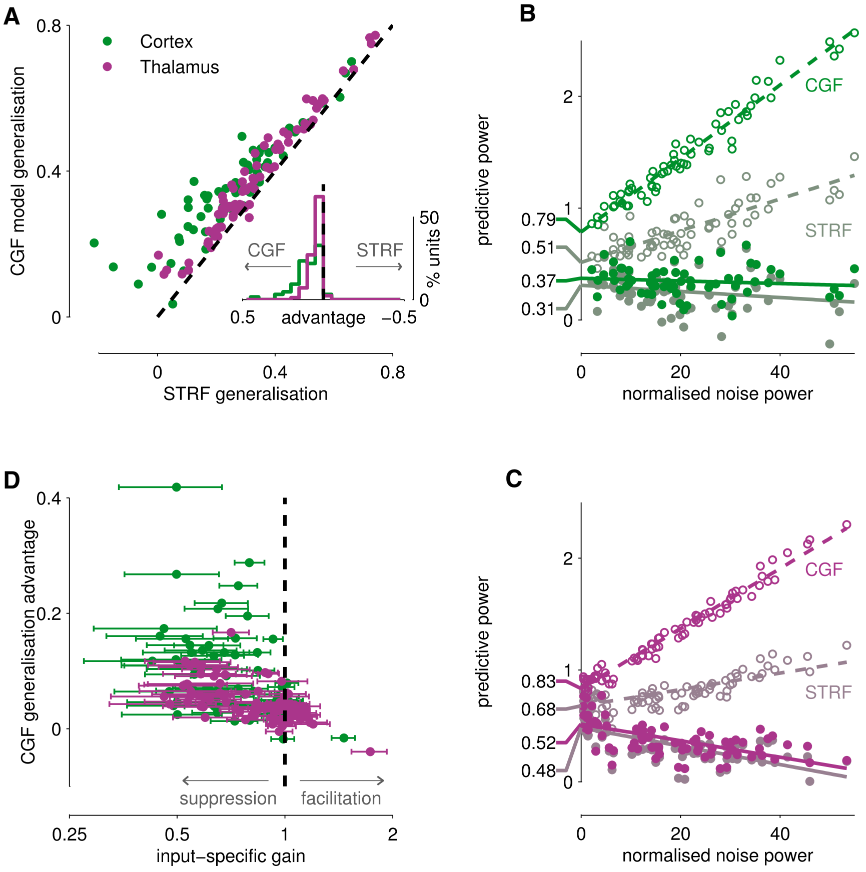

Figure 2. Contextual Input-Specific Gain Shapes Both Cortical and Thalamic Responses

(A) Scatterplot of generalization performance for the CGF and STRF models in cortex and thalamus measured by cross-validation; inset shows histogram of differences in favor of the CGF model (left) or STRF model (right). Black dashed lines indicate equal performance. The CGF model almost always generalizes more accurately than the STRF, showing that contextual input-specific gain plays a substantial role in shaping responses in both brain structures.

(B and C) Predictive power extrapolations for CGF model (bright colors) and STRF model (dull, greyed colors) in cortex (B) and thalamus (C). Filled circles and solid lines indicate generalization performance on test data, assessed by cross-validation; open circles and dashed lines show predictive performance on training data. In the zero-noise limit, extrapolated intercepts (indicated on the left) are all higher for the CGF model. See Supplemental Experimental Procedures for further explanation.

(D) Effective input-specific gains and predictive advantage. Each dot and horizontal bar indicates the median and interquartile range of the distribution of effective input-specific gains across all points in the stimulus for one neuron, obtained by convolving the spectrogram of the DRC stimulus with the neuron's CGF (see also Figure 3). Median input-specific gains tend to be substantially smaller than 1 and interquartile ranges are often large, indicating that effects of local acoustic context are predominantly suppressive but can vary substantially across spectrotemporal points within the DRC stimulus.

tractably as possible, we made three key simplifying assumptions: first that energy in the local context is integrated linearly within the CGF, second that the output of this CGF-weighted sum linearly affects the input gain, and third that the CGF weights are the same at each point in the PRF. The result is the multiplicative model of Equation 1, in which the dependence of the firing rate on the sound energy is quadratic. This model thus represents a constrained second-order Volterra expansion, in which the linear kernel is the PRF, and the quadratic kernel is formed from suitably selected products of weights in the PRF and CGF. Using the same CGF weights at each PRF input reduces the total number of parameters that must be fit (1,046) far below that needed to descibe an unconstrained second-order Volterra expansion using the same window size as the PRF (just under 52,000). Prohibitive volumes of physiological data would have been required to fit the unconstrained model.

The single-CGF assumption was supported by the observation that in a dual-CGF version of the model, with potentially different CGFs fit to two pre-selected portions of the PRF—for example, to the excitatory and inhibitory regions—the two learnt CGFs were consistently similar (Figure S2). The dual-CGF model is also a second-order Volterra model but enforces slightly less severe constraints on the quadratic kernel than the single-CGF model. Despite the added degrees of freedom, the dual model added no further generalization ability.

The CGF formulation was also supported by comparison to a "low-dimensional" quadratic model, similar to that described by Park et al. (2013), in which the second-order kernel matrix is approximated by a sum of vector outer products. The dimensionality is given by the number of products in this sum. A one- or two-dimensional quadratic model has comparable degrees of freedom to the CGF model, but quite different constraints; indeed, it can be viewed as a low-dimensional LN model (Figure 1B) with a second-order polynomial nonlinearity (Supplemental Experimental Procedures 4). Neither one-dimensional nor two-dimensional quadratic models generalized as well as the CGF model, as measured by cross-validation (Figure S3). Indeed, the one-dimensional model also fit the *training data* less well, despite having more than twice the degrees of freedom (720 versus 324). Thus the LN structure of the outer-product quadratic form is not as well suited to capture the stimulus-evoked response even in training data. To outperform the CGF model on the training data it was necessary to include at least two outer products in the quadratic kernel, adding more than four times as many parameters as in the CGF model—and this two-dimensional quadratic model did not generalize as well as the CGF model, even after regularization. Noise-discounted extrapolated population values of predictive power for the one-dimensional and two-dimensional quadratic models were ([test, training] values) as follows: cortex 1D quadratic = [0.34,

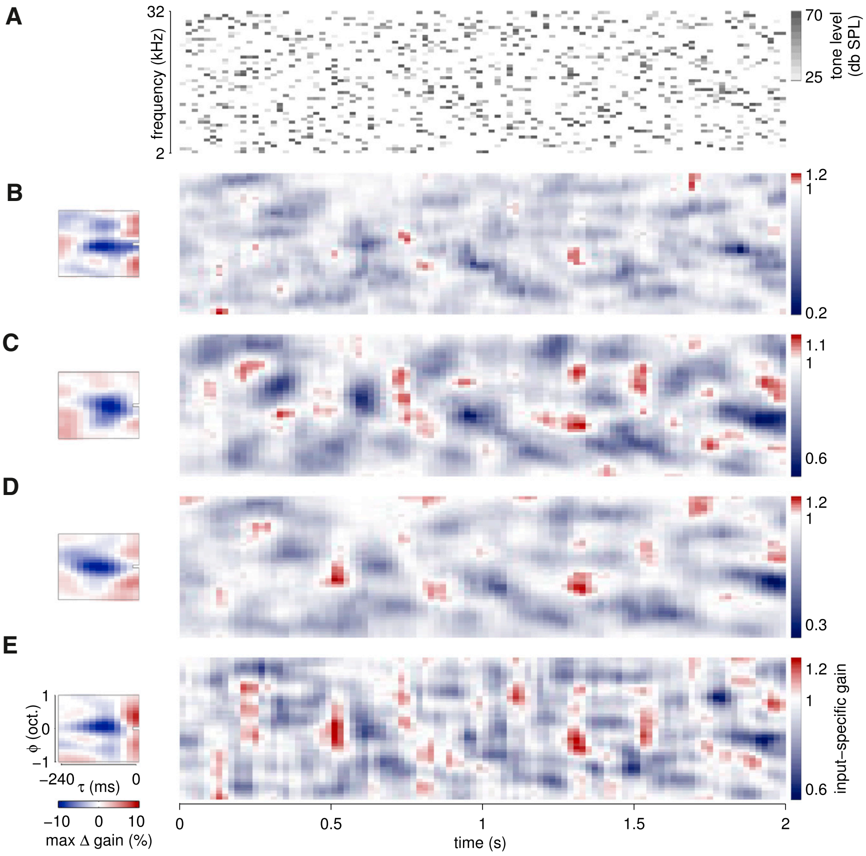

Figure 3. Variation in Contextual Input-Specific Gain across Spectrotemporal Points within a Complex Stimulus

(A) Two-second-long segment of the DRC stimulus.

(B–E) CGFs (left) for four example cortical neurons are convolved with the spectrogram of the DRC stimulus to reveal effective input-specific gains (right) that vary substantially from cell to cell, frequency to frequency and moment to moment within the stimulus.

0.64]; thalamus 1D quadratic = [0.49, 0.75]; cortex 2D quadratic = [0.33, 0.98]; thalamus 2D quadratic = [0.51, 0.99].

Thus, we conclude that the CGF parameterization of the second-order Volterra kernel provides a particularly biologically relevant—and analytically tractable—description of nonlinear constraints on auditory cortical processing.

Input-Specific Gain Modulation Is Substantial and Predominantly Suppressive

The substantial impact of immediate acoustic context on cortical and thalamic responses was also evident in the moment-by-moment variation of input-specific gains inferred by the CGF model (Figures 2D and 3). We convolved the spectrogram of the DRC with each neuron's CGF, obtaining an estimate of the "effective input-specific gain" set by the local acoustic context at each point in the stimulus. A constant gain of 1 would imply a linear response; effective gains greater than 1 occur at points in the spectrogram where the neuron's sensitivity is boosted by local acoustic context; and values below 1 occur where sensitivity is locally suppressed. The effective input-specific gain for each neuron varied substantially from moment to moment and frequency to frequency (for examples, see Figure 3), typically ranging between slightly facilitatory and substantially suppressive. Furthermore, differences in CGFs meant that the detailed pattern of gains differed from cell to cell.

To quantify the overall impact and variability of input-specific gain modulation, we computed the quartile points of the distribution of effective gains across the DRC stimulus, both for each neuron individually and for pooled neuronal populations. Gains varied substantially, with large interquartile ranges for most neurons (Figure 2D) and both cortical and thalamic populations (0.37 and 0.39, respectively). Many of these interquartile gain ranges did not include 1 (for 50/64 cortical neurons and 46/101 thalamic neurons), indicating a pervasive and systematic impact of immediate context. In the pooled distributions, the median input-specific gain was significantly smaller than 1 (0.73 in cortex and 0.86 in thalamus; $p <$ machine precision), indicating a predominantly suppressive effect. Furthermore, the predictive advantage of the CGF model over the STRF model increased both as the inter-quartile range of effective input-specific gains increased (i.e., as gain varied more widely over the course of the stimulus; Spearman's $\rho_{(N=165)} = 0.31$, $p = 7.2 \times 10^{-5}$), and as the median input-specific gain decreased (i.e., as contextual suppression increased; $\rho_{(N=165)} = -0.62$, $p = 1.6 \times 10^{-14}$).

Two Key Features of Input-Specific Gain Modulation in Cortex and Thalamus

The structures of the CGF and PRF fit to a neuron's response reveal how input-specific gain depends on local context, and how it interacts with spectrotemporal integration in the neuron. PRFs differed from STRFs in a predictable manner (Figure S4; Supplemental Experimental Procedures 5), and comparisons of either PRF or STRF structure between cortex and thalamus yielded results similar to previous reports for STRFs (e.g., longer receptive field durations in cortex than thalamus; Miller et al., 2002). Our focus here is on novel findings revealed by the CGFs.

CGFs in both cortex and thalamus displayed consistent features, albeit with different timescales at the two stages of auditory processing. Most cells (examples in Figure 4A) exhibited a suppressive region of negative CGF weights centered at a zero frequency offset, and extending over much of the CGF time window. Thus, preceding sound energy at a similar frequency tended to dampen the impact of a component sound, reducing excitation or inhibition as the component fell within the positive or negative part of the PRF, respectively. This

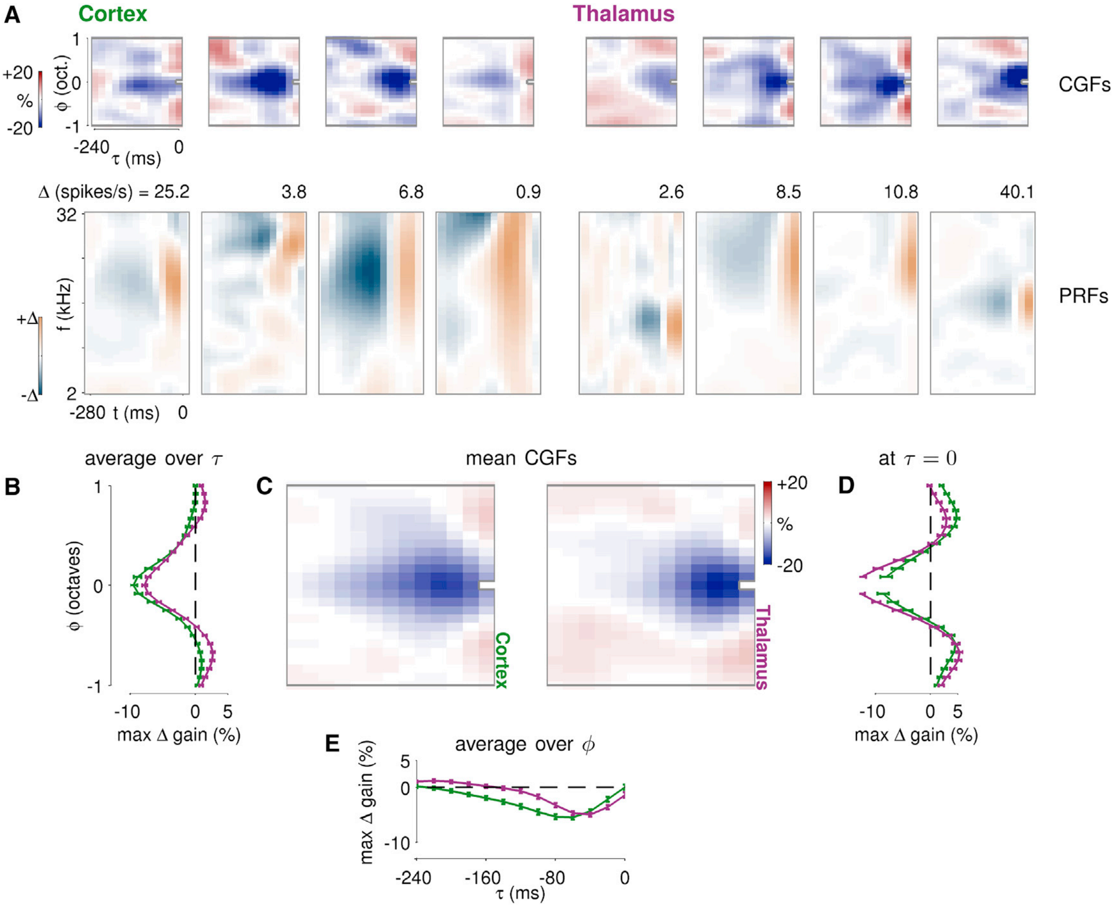

Figure 4. Structure of Input-Specific Gain Modulation in the Cortex and Thalamus

(A) Example CGF and PRF pairs for four neural recordings in cortex (left) and four recordings in thalamus (right). CGFs (top) range over relative time τ and relative frequency ϕ. Weights represent the change in gain induced if one of the loudest tones of the DRC stimulus were to fall at the corresponding location, and are shown on common scale (left). PRFs (bottom) range over time t prior to the modeled response and acoustic frequency f (log-spaced). Stimulus modulation of firing differs substantially across neurons, so PRFs are separately (and symmetrically) scaled to the maximum change in firing rate shown above each one.

(B–E) Mean CGFs and average profiles in cortex (green) and thalamus (magenta). The central panel (C) shows the spectrotemporal pattern of the mean CGF weights in both structures. The average spectral profiles (B), spectral profiles at 0 delay (D) and average temporal profiles (E) of both means are shown superimposed, with error bars indicating the SE of the estimated population means.

suppressive effect lasted longer in cortex than in thalamus. Also visible in the example CGFs is a halo of gain-enhancing regions around the suppressive center. At long time delays, the structure of these gain-enhancing regions varied considerably from neuron to neuron. However, there appeared to be a consistent enhancement associated with simultaneous or near-simultaneous sound energy at both positive and negative frequency offsets.

Since the CGF ranges over *relative* time and frequency offset (τ and ϕ) it is possible to examine the common stucture of contextual effects by averaging the CGFs of a population of neurons. The two phenomena visible in the examples—delayed suppression and near-simultaneous enhancement—are also evident in the mean CGFs for both cortex and thalamus (Figure 4C), and in the one-dimensional profiles averaged over all τ (Figure 4B), restricted to $\tau = 0$ (Figure 4D), and averaged over all ϕ (Figure 4E). Similar results were obtained when the averages were restricted to subsets of neurons grouped by best frequency as estimated from the PRF or STRF (data not shown), demonstrating that the mean CGFs are representative of neurons tuned to all points of the frequency spectrum.

Delayed input-specific gain suppression was centered around a frequency offset of zero in both areas (Figure 4B), but peaked at a greater delay (60–80 ms compared to 40 ms) and extended to longer temporal offsets (160 ms compared to 100 ms) in cortex than in thalamus (Figure 4E). While reminiscent of forward suppression of responses to repeated tones, the modulatory rather than inhibitory effect of the CGF also implies suppression of inhibitory gains, which mght sometimes lead to enhanced responses in complex stimuli. Indeed, the dual-CGF model (Figure S2) confirmed that the contextual influence on inhibitory and excitatory PRF inputs showed similar suppression.

Gain enhancement resulted from sound energy that fell at short time offsets within the CGF window and outside the central

suppressive region (Figure 4D). In the cortex this facilitation was clearly strongest and most consistent at time offsets <40 ms, and peaked at frequency offsets of about a half-octave in either direction (Figure 4D). The same short-time effect was also evident in thalamus; indeed, the mean CGF profile at zero time-offset was remarkably similar to that observed in cortex (Figure 4D), with off-frequency peaks approximately a half-octave away from the center. It is difficult to tell from the mean CGFs alone whether these off-frequency peaks reflect a mechanism of gain facilitation specific to half-octave frequency intervals, or whether they emerge from the interplay of two seperate mechanisms: broadband near-simultaneous enhancement centered at zero frequency offset, and narrowband delayed suppression that cancels the enhancement at small frequency and time offsets. However, the observation that similar side-peaks appeared in individual CGFs (Figure 4A) implies that the structure observed in the means does not arise from broad facilitation and narrow suppression contributed by different neurons.

Individual CGFs often showed both narrowband delayed suppression and broadband near-simultaneous enhancement (Figure 4A), suggesting that CGFs are not time-frequency separable. Indeed, predictive power was almost always higher for inseparable-CGF models than for separable-CGF models (Figure S1), despite the expectation that the many more parameters of the inseparable-CGF model would increase susceptibility to overfitting and thereby undermine generalization performance.

Two Key CGF Features Each Have Significant Impact on Neural Responses

We wondered whether the key features that had appeared reliably in the mean CGFs were each essential for shaping the neural responses, or whether their effects might be substituted by parameters elsewhere in the CGF or PRF. To find out, we refit "elided" versions of the model, where the range of weights corresponding to one of the features was set to zero and the remaining parameters refit to test whether they could compensate for the elision. Generalization performance was compared to that of the full CGF by cross-validation. If the feature removed is not essential, then the elided model should achieve the same generalization performance as the full model despite the feature's absence. Indeed, with fewer parameters and therefore less risk of overfitting it might generalize *more* accurately than the full CGF. Conversely, if the generalization accuracy after elision is systematically lower, then the impact of the removed CGF feature could not be matched by modifying weights in the rest of the CGF or PRF, and so the feature itself must be essential.

We found that models in which either of the two key CGF features were elided did indeed provide a poorer fit to the data than the unconstrained model (Figure 5). In particular, excluding CGF weights with frequency offsets within 1/3 octave and time offsets between 20 and 120 ms, where delayed suppression is most evident, reduced model predictive power significantly (average difference in cross-validated predictive performance between elided and full CGF models -0.030 ± 0.003 in cortex and -0.027 ± 0.002 in thalamus; Figures 5A and 5D). By contrast, elision of a similarly sized CGF region at much longer delays had no discernable effect at the population level. Eliding CGF weights at all frequency offsets and delays <40 ms, where enhancement is evident, also impaired model fits systematically (change in average cross-validated predictive power -0.017 ± 0.002 in cortex and -0.022 ± 0.002 in thalamus; Figures 5B and 5E). When the elided near-simultaneous region was restricted to short-delay CGF weights with frequency offsets greater than one-third octave, the impact on model predictions was lessened, but remained significant (Figures 5C and 5F). Again, in both cases, elision of a congruent section at much longer delays induced no discernable change in model performance. Thus, both broadband near-simultaneous facilitation and narrowband delayed suppression play significant and independent roles in shaping input-specific gain.

Detailed CGF Structure Differs between Individual Neurons

Despite their consistent features (Figure 4), the CGFs estimated for individual recordings were not identical. Moreover, at least in the cortex, this neuron-to-neuron variation appeared to contribute to the improved predictive power of the CGF model. Models with neuron-specific CGFs performed at least as well in cross-validation as models with a fixed CGF (Figure S5)—especially for recordings with the lowest normalized noise power. Had neuron-to-neuron variation in the estimated CGF structure arisen solely through noise, the individual CGFs would have overfit and the models performed more poorly.

We investigated the primary modes of variability around the mean by applying principal components analysis (PCA) to the CGFs within the cortical and thalamic populations. PCA decomposes the scatter of a multidimensional dataset into components along which the variability is uncorrelated, and which can then be ranked by the amount of variance that each contributes. In both cortex and thalamus, the scatter around the mean CGF was concentrated in a relatively small number of principal components. In particular, the first two or three principal components (PCs) stood out from the remaining modes (Figures 6A and 6D). Together, the first three PCs described 62% and 61% of the variance in the cortical and thalamic CGFs, respectively (Figures 6B and 6E).

The structure of these first modes of variability for the cortical and thalamic populations is shown in Figures 6C and 6F. In both cases, the dominant effect observed in the first PC was to modulate the overall depth of delayed suppression, either increasing or reducing it as the loading on the PC varied from positive to negative across cells. In the cortex, there was also some suggestion that the strength of this suppression was anti-correlated with broadband simultaneous facilitation, while in the thalamus the two effects were uncorrelated. The second principal mode of scatter in both structures, which carried at least half as much variance as the first in both cases, appeared to modulate the effect on contextual gain of tones at short delays and nearby frequencies. The third mode of scatter was of greater significance in the thalamus (compare Figures 6A and 6D) and reflected variability in the broadband near-simultaneous modulation of input-specific gain.

The subspace defined by the two (in cortex) or three (in thalamus) leading modes of scatter around the population mean CGFs also captured around 80% of the sum of squared weights in the means themselves (Figures 6B and 6E, open circles). This observation (and, indeed, the examples of Figure 4A) suggests

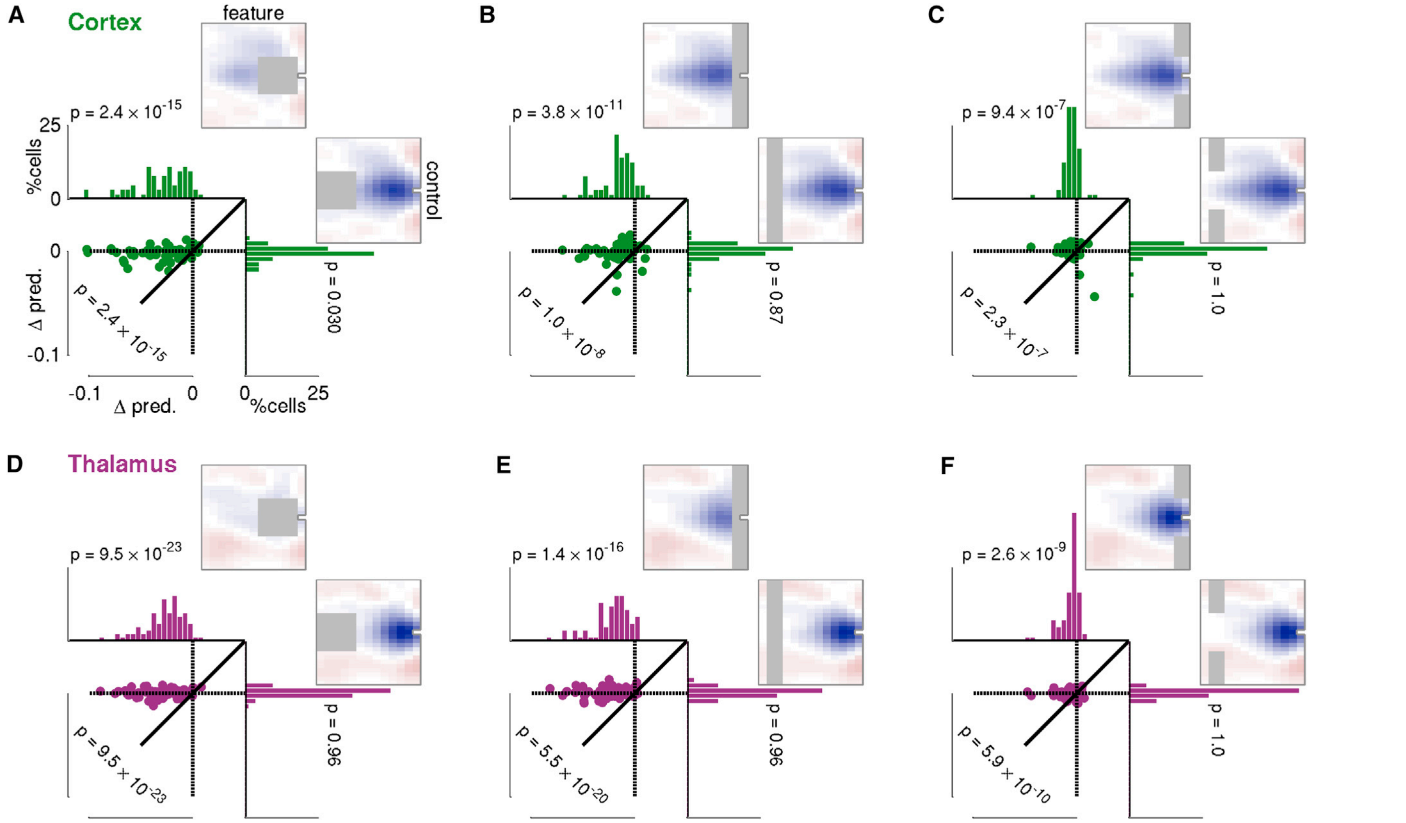

Figure 5. Generalization Disadvantage for Models with Key Features of CGF Elided

(A–C) Cortex; (D–F) thalamus. Each panel contrasts the effects of eliding parameters in two identically sized sections of the CGF (gray rectangles): one corresponding to a CGF feature that appeared to consistently shape input-specific gain, the other a control section where CGF weights were inconsistent or small. Weights in the elided regions were fixed at zero, and the model was re-fit to optimize the remaining model parameters. Histograms show distribution across neurons of differences in cross-validation predictive performance (generalization accuracy) relative to the unelided CGF model; p value indicates significance threshold at which the hypothesis that median change in performance equals or exceeds zero can be rejected (one-tailed sign test, uncorrected; *N* = 64 in cortex and 101 in thalamus). Scatter plots compare generalization accuracy of the two elided models neuron-by-neuron; p value indicates threshold for rejection of the hypothesis that median difference for feature elision minus control elision equals or exceeds zero (one-tailed sign test, uncorrected). Across the neural population, elision of key CGF features always resulted in poorer generalization accuracy than that achieved by the full (unelided) model. By contrast, control elisions had significantly less impact; the hypothesis that control elisions produced no reduction in predictive performance could not be rejected in any case after correction for multiple comparisons.

that contextual gain modulation involves the interplay of two (or perhaps three) different functional mechanisms contributing in varying degrees to each neuron's individual contextual gain field, and therefore that the mean CGFs of Figure 4C reflect the average impact of each of these mechanisms across the population.

Detailed CGF Structure Differs between A1 and AAF, but Not between vMGB and mMGB

Our cortical dataset comprised 31 recordings from primary auditory cortex (A1) and 33 from anterior auditory field (AAF), localized physiologically by a reversal of tonotopy (Stiebler et al., 1997; Linden et al., 2003; Guo et al., 2012; Issa et al., 2014). Both A1 and AAF are "core" auditory fields that receive strong thalamic input (Lee et al., 2004; Hackett et al., 2011). Previous studies have revealed differences between A1 and AAF in the temporal extent of STRFs (Linden et al., 2003); these results were confirmed here for PRFs (data not shown). We found that nonlinear context effects also differed between the two cortical areas. Mean CGFs for both A1 and AAF (Figures 7A–7D) exhibited the two key features seen in the overall mean. However, the delayed suppression region of the CGF peaked at smaller delays (60 ms versus 80–100 ms) and was shorter overall (140 ms versus 160–180 ms) in AAF than in A1 (Figure 7C). Spectral profiles for near-simultaneous gain enhancement were similar in shape in A1 and AAF, although the magnitude of the effect appeared stronger in A1, and facilitatory side peaks fell at slightly larger frequency offsets in AAF (Figure 7D).

In contrast, we found no significant differences in detailed CGF structure between two thalamic subdivisions. Our set of 101 thalamic recordings included 51 from the ventral subdivision of the medial geniculate body (vMGB) and 34 from the medial subdivision (mMGB); subdivision assignments were determined histologically through reconstruction of recording sites in sections stained for cytochrome oxidase (Anderson and Linden 2011). (We did not obtain enough recordings from the third major subdivision, the dorsal MGB, to justify including those recordings

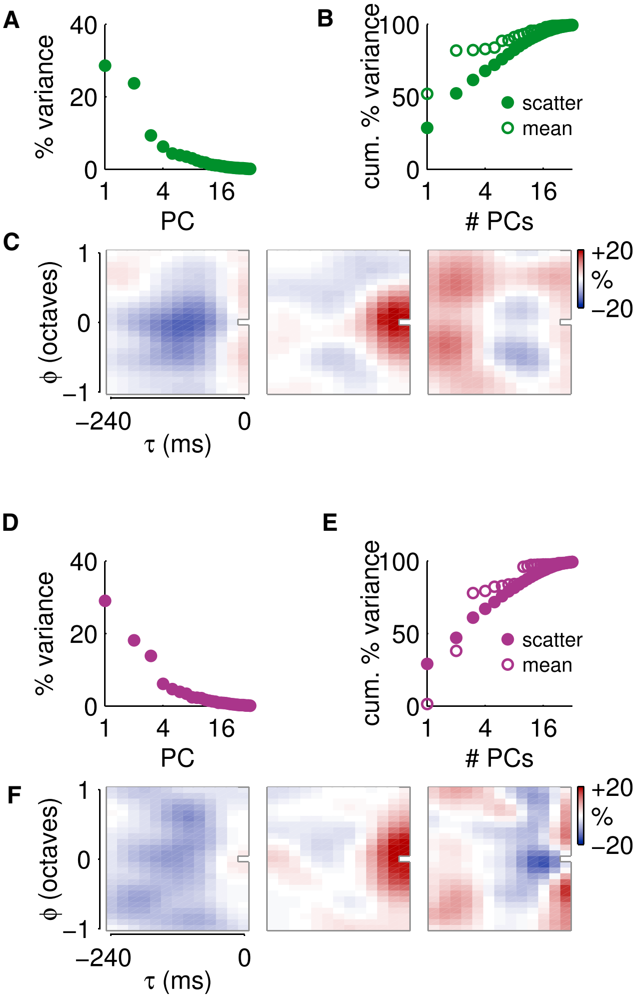

Figure 6. Variability in CGF Structure across Neurons

(A–C) PCA of CGFs in the cortex.

(A) The absolute variance (i.e., average squared Δ gain) captured by each of the first 32 PCs. (PC numbers are plotted logarithmically.)

(B) Filled symbols: fractional variance in the CGFs captured by the leading PCs, as a function of number of PCs considered. Open symbols: fractional sum of squares of the *mean* cortical CGF that projects into the space spanned by the leading PCs, demonstrating how well the variance is aligned with the mean.

(C) The three leading PCs in order from left to right.

(D–F) PCA of CGFs in the thalamus. Subpanels correspond to (A)–(C).

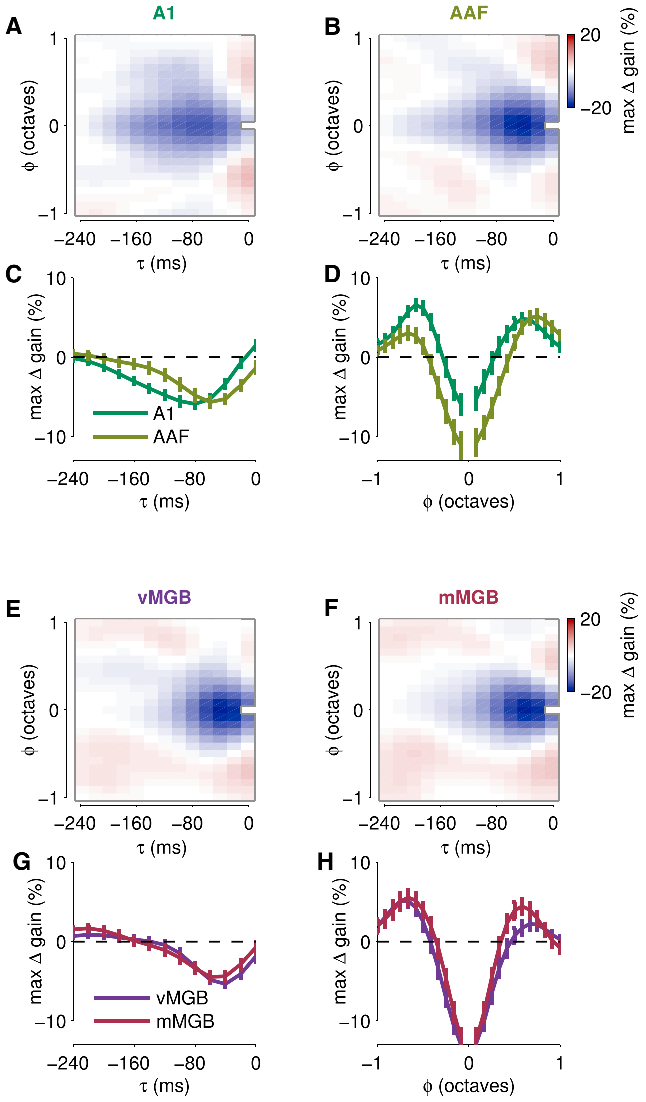

Figure 7. Contextual Input-Specific Gain Compared between Two Cortical Fields and Two Thalamic Subdivisions

(A and B) Mean CGFs of neurons in cortical areas A1 and AAF. Overall structure is similar in both areas, but the delayed suppression region is shifted toward shorter delays in AAF.

(C) Mean temporal CGF profiles averaged over frequency offset for both areas (error bars show standard errors in the mean). The shorter delay and shorter duration of the suppressive contextual gain effect within AAF is clearly evident.

(D) Mean spectral CGF profiles at zero time lag (error bars show standard errors in the mean). The general shape of the spectral interaction is similar in the two cortical areas, although side peaks in AAF fall at slightly larger frequency offsets, perhaps as a result of the stronger short-delay suppression in AAF.

(E–H) Similar figures show contextual gain effects in the ventral and medial subdivisions of MGB. No substantial differences are observed between these two thalamic subdivisions.

in the analysis.) The average CGFs in vMGB and mMGB were very similar (Figures 7E and 7F), with overlapping temporal (Figure 7G) and spectral (Figure 7H) profiles. The absence of clear differences in contextual input-specific gain modulation between these two thalamic subdivisions suggests that differences seen in the cortical field averages might arise intracortically.

DISCUSSION

Contextual Modulation of Sensory Coding

The neural representation of sensory information is modified by context in many ways. Previous reports have focused on how sensory representations depend on long-term or global stimulus properties or statistics (e.g., Heeger, 1992; Carandini et al., 1997; Schwartz and Simoncelli, 2001; Blake and Merzenich, 2002; Valentine and Eggermont, 2004; Gill et al., 2006; Bar-Yosef and

Nelken, 2007; Rabinowitz et al., 2011; Rabinowitz et al., 2012; Mesgarani et al., 2014), or on behavioral or attentional context (e.g., Fritz et al., 2003, 2007; Atiani et al., 2009; David et al., 2012). By contrast, the current results highlight the dependence of spectrotemporal input-specific gain on fluctuations in immediate local sensory context, even within a statistically stationary stimulus and in an anaesthetised animal.

Changes in global stimulus statistics are necessarily associated with changes in the statistics of local context, and so local modulation may contribute to apparently global effects. For example, the apparent adaptation of STRFs to spectrotemporal density or modulation (Blake and Merzenich, 2002; Valentine and Eggermont, 2004; Gill et al., 2006) may arise in part because denser stimuli drive greater local suppression of input-specific gain across the receptive field (see also Ahrens et al., 2008a; Supplemental Experimental Procedures 5). Similarly, the apparent boost of STRF weights near the spectral edges of a band-limited DRC stimulus, as seen in cat auditory cortex (Gourévitch et al., 2009), may reflect the absence of suppressive drive coming from the part of the CGF around those inputs that falls outside the pass-band of the stimulus. Interactions between contextual input-specific gain modulation and linear STRF estimates may be larger and more idiosyncratic with more structured stimuli, including natural sounds—or even artificial stimuli with nonindependent energy distributions such as spectrotemporal ripples—as nonlinear spectrotemporal contextual effects are then less likely to average away (even if the stimulus set is uncorrelated overall; Christianson et al., 2008).

The local context dependence of input-specific gain may also contribute to some forms of stimulus-specific adaptation (SSA) to tones (Ulanovsky et al., 2003; Ulanovsky et al., 2004; Anderson et al., 2009; Antunes et al., 2010), a phenomenon usually interpreted as arising from long-term stimulus predictability. Although SSA in the cortex and thalamus may persist for a second or more, SSA to tones is strongest at shorter time intervals and develops after only one or two tone repetitions (Ulanovsky et al., 2004; Antunes et al., 2010; Bäuerle et al., 2011). Such rapid stimulus-specific suppression in tone sequences is consistent with the input-specific narrowband delayed suppression observed here with complex stimuli (see also Ulanovsky et al., 2004; Mill et al., 2011; Nelken, 2014).

Response Nonlinearities

As in many studies of the visual (reviewed by Schwartz et al., 2006; Sharpee, 2013) and somatosensory (e.g., Maravall et al., 2013) systems, responses to complex auditory stimuli have widely been modeled as nonlinear functions of the *output* of one or a few linear STRF-like filter(s) (Figures 1A and 1B). This approach encompasses models of contrast gain control (Rabinowitz et al., 2011, 2012); LN models derived by spike-triggered covariance (STC), Maximally Informative Dimensions (MID), and similar methods (Atencio et al., 2008, 2009, 2012; Sharpee, 2013); LNLN cascade models of excitatory and inhibitory interaction (Schinkel-Bielefeld et al., 2012); and models of noise-invariant cortical responses combining activity-dependent subtractive depression and multiplicative gain control (Mesgarani et al., 2014). These models all start with fixed input fields, with non-linearities acting only after integration. Similarly, studies of adaptive coding (Brenner et al., 2000; Fairhall et al., 2001; Maravall et al., 2007; Mease et al., 2013) have considered context-dependent changes in a single, global, input gain factor (often determined by long-term temporal contrast) that applies after integration but before further nonlinear transformations (Figure 1C). Neither approach captures the input-specific gain modulation described here, in which different context-sensitive input gains act at different points in spectrotemporal space *before integration*. It is likely that both input and output nonlinearities, as well as spike-dependent temporal interactions (Chornoboy et al., 1988; Ahrens et al., 2008b), combine to shape responses in auditory and in other sensory systems.

The closest analogs to the present results use second-order Volterra or similar models to characterize either spectral or temporal nonlinearities (in cortex: Pienkowski and Eggermont, 2010; Pienkowski et al., 2009; David and Shamma, 2013; see also Yu and Young, 2000, in cochlear nucleus). Indeed, the CGF model is a second-order Volterra expansion of *spectrotemporal* nonlinearites, with a constrained quadratic interaction that both provides a ready interpretation of the nonlinearity in terms of modulatory local context effects and keeps the number of parameters within a range that is feasible to fit with limited experimental data. The present study also builds upon previous work by the some of the current authors (Ahrens et al., 2008a), which introduced the multilinear estimation framework and demonstrated the importance of modeling input nonlinearities for accurate prediction of cortical responses to complex sounds. However, these previous studies did not investigate the impact of the full, inseparable, spectrotemporal context—nor compare contextual influence across brain areas. The inseparable spectrotemporal structure of CGFs revealed by the extended model developed here has important implications for understanding auditory processing of complex sounds.

Implications for Auditory Perception and Neural Processing

Narrowband Delayed Suppression

Narrowband delayed suppression in cortical and thalamic CGFs is likely to relate to both the psychophysical phenomenon of forward masking and the physiological phenomenon of forward suppression. In both humans and mice, psychophysical sensitivity to the second of two sounds with similar frequencies is reduced for more than 100 ms after the offset of the first sound (Jesteadt et al., 1982; Walton et al., 1995), consistent with the duration of narrowband suppression seen here in mouse cortex and thalamus. A similar (and putatively related) forward suppression is often observed in central auditory responses to tone pairs (Calford and Semple, 1995; Brosch and Schreiner, 1997; Wehr and Zador, 2005); and although tones played against a *silent* background may also sometimes facilitate later responses (Brosch et al., 1999; Brosch and Schreiner, 2000; Wehr and Zador, 2005), forward suppression appears to dominate in complex *continuous* sounds (e.g., in awake ferrets; David and Shamma, 2013), consistent with our CGF observations.

Forward suppression could arise from direct linear (subtractive) inhibition, output gain (divisive) inhibition, or modulation of input gain. Although measures of suppression in previous studies might include contributions from all three mechanisms,

the duration of suppressive effects in awake and anaesthetised animals of many species is similar to that of the contextual influences seen here (Creutzfeldt et al., 1980; Scholl et al., 2010; David and Shamma, 2013). Furthermore, forward suppression in tone pairs appears to outlast inhibitory currents in the target cell (Wehr and Zador 2005) and is specific to particular input synapses (Scholl et al., 2010). Thus, narrowband delayed suppression in the CGF could be an analog, in neural responses to complex sounds, of this input-specific nonlinear component of forward suppression in responses to tones. Such suppression could arise from either synaptic depression or spike-rate adaptation along the auditory pathway. These adaptive mechanisms acting at subthalamic stages of auditory processing (including in the inferior colliculus) might drive narrowband suppression at the shortest timescales in both cortex and thalamus, while differences between cortex and thalamus (and between A1 and AAF) in suppression at longer timescales might reflect the cascaded contributions of thalamocortical and intracortical adaptation. Similar adaptive cascades have been hypothesized to underlie the hierarchical emergence of deviance sensitivity in the central auditory system at much longer timescales (Mill et al., 2011).

Broadband Near-Simultaneous Enhancement

The nonlinear augmentation of both excitatory and inhibitory input gain by broadband near-simultaneous sound energy may be a neural correlate of perceptual sensitivity to common onsets. Simultaneous onsets at different frequencies are salient even in a complex sound environment, and guide both auditory stream segregation and object identification (Bregman, 1994; Shamma et al., 2011). While this perceptual phenomenon has long been recognized (Bregman, 1994), a systematic neural correlate has been elusive. Responses to simultaneous tone pairs or complexes display a variety of cell-specific facilitatory and suppressive effects in the auditory cortex (Shamma et al., 1993; Calford and Semple, 1995; Sutter et al., 1999; Kadia and Wang, 2003; Sadagopan and Wang, 2009), arising largely from two-tone (second-order) interactions (Nelken et al., 1994a, 1994b). Neurons in the auditory cortex and thalamus of the bat show augmented responses to particular combinations of sound frequency corresponding to the harmonics of sonar calls and their echoes (e.g., Suga et al., 1979, 1983; Olsen and Suga, 1991; Wenstrup, 1999). However, no systematic motif of gain enhancement by near-simultaneous pairings has emerged, and it has remained unclear how sound combinations interact with integration within the receptive field. It may be that near-simultaneous gain facilitation becomes significant only during auditory processing of complex broadband sounds (cf. Nelken et al., 1997; Rotman et al., 2001), when the preferential processing of simultaneous onsets may be most functionally important.

It is unclear whether the off-frequency peaks we observed reflect a special sensitivity to half-octave separations or an interaction between a broad central peak of simultaneous enhancement and the earliest component of narrowband delayed suppression. The latter scenario might arise from integration of broadly tuned subthreshold inputs with strong short-term synaptic depression, consistent with reports that short-term spectral integration in the auditory cortex extends over one to two octaves (Kaur et al., 2004; Metherate et al., 2005). On the other hand, neural mechanisms associated with psychoacoustic "critical bands," which are approximately one-third-octave wide in the mouse (Egorova et al., 2006; Egorova and Ehret, 2008), might conceivably favor nonlinear interactions between sounds one critical band apart. For example, off-frequency CGF peaks might arise from coherent modulations in adjacent frequency laminae of the inferior colliculus, which discretize the midbrain tonotopic gradient into approximately critical-band intervals (Schreiner and Langner, 1997; Malmierca et al., 2008).

Variability across Cells and Brain Areas

The shapes of CGFs are notable for their variability as well as for their consistency. Analysis of principal components of the CGFs revealed cell-to-cell variability in the overall depth of delayed suppression and the strength of off-frequency near-simultaneous enhancement. Thus, the consistent features of the mean CGFs are not uniformly inherited from peripheral nonlinearities but arise through the combination of different gain-modulation profiles in individual cortical or thalamic neurons.

The systematic temporal variations in CGFs between thalamus and cortex, and A1 and AAF, are consistent with temporal properties observed in STRFs (Miller et al., 2002; Linden et al., 2003), suggesting that both linear and nonlinear components of forward suppression operate on faster timescales in thalamus than cortex, and in AAF than A1. In contrast, spectral profiles for near-simultaneous gain enhancement were very similar in cortex and thalamus, and also in A1 and AAF, although the magnitude of the effect appeared stronger in A1, and facilitatory side peaks fell at slightly larger frequency offsets in AAF. However, if the apparent peaks arise through a combination of broad facilitatory bumps and the effects of narrowband suppression, then these small differences may simply result from the deeper short-term suppression in AAF.

The consistency of CGFs in ventral and medial MGB accords with the general similarity of many response properties in mouse vMGB and mMGB (Anderson and Linden 2011) but seems surprising given known differences between the subdivisions in sensitivity to stimulus context over much longer timescales (Anderson et al., 2009; Antunes et al., 2010; Antunes and Malmierca, 2011). Thus, the relatively fast context effects studied here may differ from mechanisms of long-term SSA, even if they underlie the short-term component, as we suggest above. Furthermore, consistent contextual gain modulation in auditory thalamic subdivisions also suggests that the CGF differences between cortical fields A1 and AAF (especially in the temporal profiles for delayed suppression) may arise at the thalamocortical synapse or intracortically, rather than from differing patterns of thalamic input.

Conclusion

The neuronal representation of sound is transformed by nonlinear mechanisms as it ascends the auditory pathway. These mechanisms manifest as the nonlinear interactions within the RFs of neurons in thalamus and cortex captured by the CGF. Similar effects may shape sensory coding in other brain areas and sensory modalities, and adapt across different behavioral contexts.

EXPERIMENTAL PROCEDURES

Detailed descriptions appear in Supplemental Experimental Procedures 1.

Surgical Procedures

Subjects were adult male mice of the CBA/Ca inbred strain. Mice were maintained at a surgical plane of anesthesia with ketamine and medetomodine. Cortical surgical procedures were as described by Linden et al. (2003) and conformed to protocols approved by the Committee on Animal Research at the University of California, San Francisco, which were in accordance with federal guidelines for care and use of animals in research in the United States. Thalamic surgical procedures were similar and were performed under a license approved by the UK Home Office in accordance with the United Kingdom Animal (Scientific Procedures) Act of 1986.

Recording Procedures

Extracellular recordings were obtained from the auditory cortex and thalamus using single or multiple tungsten electrodes, and spike-sorted off-line to extract responses from either small clusters of neurons or well-isolated single units. Cortical areas A1 and AAF were identified physiologically, and thalamic subdivisions vMGB and mMGB were identified histologically.

Stimuli

All experiments were conducted in a sound-shielded anechoic chamber (Industrial Acoustics). Auditory stimuli were directed toward the ear contralateral to the recording site via a free-field speaker, and a sound-attenuating plug was placed in the ipsilateral ear. Prior to the start of each experiment, acoustic stimuli were calibrated with a Brüel and Kjær 1/4" microphone positioned near the opening of the animal's auditory canal. Typically, the calibration ensured that the frequency response of the sound system was flat to within ±2 dB over 2–90 kHz.

A 2–32 kHz dynamic random chord (DRC) stimulus described previously by Linden et al. (2003) was used for both cortical and thalamic experiments. While much simpler in structure than many natural sounds, the DRC stimulus can be considered a complex stimulus in that it contains a huge variety of spectrotemporal conjunctions of tonal elements, which provide a substrate for combination-sensitive nonlinearities (such as those captured by the CGF) to act.

Data Analysis and Modeling

From larger databases of cortical and thalamic recordings collected during presentations of the DRC stimulus, we selected for analysis here those recordings with significantly nonzero stimulus-dependent signal power (see Supplemental Experimental Procedures 3). In order to enable comparisons between brain regions, we further restricted our attention to those recordings that had been reliably localized to A1 or AAF within the auditory cortex, or to vMGB or mMGB within the auditory thalamus. We then fit both linear STRF models and multilinear CGF models to the DRC-evoked neural responses.

SUPPLEMENTAL INFORMATION

Supplemental Information includes five figures and Supplemental Experimental Procedures and can be found with this article at http://dx.doi.org/10.1016/j.neuron.2016.05.041.

AUTHOR CONTRIBUTIONS

J.F.L. collected all cortical data, and R.S.W. collected all thalamic data following similar procedures. M.B.A. and M.S. developed the CGF model, and R.S.W. and M.B.A. implemented the parameter identification algorithm used here. All authors designed the analyses, which R.S.W., M.B.A., and M.S. implemented. J.F.L. supervised experimental work, M.S. supervised the model development and implementation, and both oversaw the analysis of the results. M.S., J.F.L., and R.S.W. cowrote the manuscript, with input from M.B.A.

ACKNOWLEDGMENTS

We thank Lucy Anderson for expert guidance and assistance with histology and thalamic recording procedures, including design and fabrication of custom electrodes. This work was supported by the Centre for Mathematics and Physics in the Life Sciences and Experimental Biology at University College London (R.S.W.), the Wellcome Trust (M.B.A. and J.F.L.), the National Institutes of Health (J.F.L.), and the Gatsby Charitable Foundation (M.S. and J.F.L.).

Received: February 24, 2015
Revised: October 25, 2015
Accepted: May 12, 2016
Published: June 23, 2016

REFERENCES

Aertsen, A.M., Johannesma, P.I., and Hermes, D.J. (1980). Spectro-temporal receptive fields of auditory neurons in the grassfrog II. Analysis of the stimulus-event relation for tonal stimuli. Biol. Cybern. *38*, 235–248.

Ahrens, M.B., Linden, J.F., and Sahani, M. (2008a). Nonlinearities and contextual influences in auditory cortical responses modeled with multilinear spectrotemporal methods. J. Neurosci. *28*, 1929–1942.

Ahrens, M.B., Paninski, L., and Sahani, M. (2008b). Inferring input nonlinearities in neural encoding models. Network *19*, 35–67.

Anderson, L.A., and Linden, J.F. (2011). Physiological differences between histologically defined subdivisions in the mouse auditory thalamus. Hear. Res. *274*, 48–60.

Anderson, L.A., Christianson, G.B., and Linden, J.F. (2009). Stimulus-specific adaptation occurs in the auditory thalamus. J. Neurosci. *29*, 7359–7363.

Antunes, F.M., and Malmierca, M.S. (2011). Effect of auditory cortex deactivation on stimulus-specific adaptation in the medial geniculate body. J. Neurosci. *31*, 17306–17316.

Antunes, F.M., Nelken, I., Covey, E., and Malmierca, M.S. (2010). Stimulus-specific adaptation in the auditory thalamus of the anesthetized rat. PLoS ONE *5*, e14071.

Atencio, C.A., Sharpee, T.O., and Schreiner, C.E. (2008). Cooperative nonlinearities in auditory cortical neurons. Neuron *58*, 956–966.

Atencio, C.A., Sharpee, T.O., and Schreiner, C.E. (2009). Hierarchical computation in the canonical auditory cortical circuit. Proc. Natl. Acad. Sci. USA *106*, 21894–21899.

Atencio, C.A., Sharpee, T.O., and Schreiner, C.E. (2012). Receptive field dimensionality increases from the auditory midbrain to cortex. J. Neurophysiol. *107*, 2594–2603.

Atiani, S., Elhilali, M., David, S.V., Fritz, J.B., and Shamma, S.A. (2009). Task difficulty and performance induce diverse adaptive patterns in gain and shape of primary auditory cortical receptive fields. Neuron *61*, 467–480.

Bar-Yosef, O., and Nelken, I. (2007). The effects of background noise on the neural responses to natural sounds in cat primary auditory cortex. Front. Comput. Neurosci. *1*, 3.

Bar-Yosef, O., Rotman, Y., and Nelken, I. (2002). Responses of neurons in cat primary auditory cortex to bird chirps: effects of temporal and spectral context. J. Neurosci. *22*, 8619–8632.

Bäuerle, P., von der Behrens, W., Kössl, M., and Gaese, B.H. (2011). Stimulus-specific adaptation in the gerbil primary auditory thalamus is the result of a fast frequency-specific habituation and is regulated by the corticofugal system. J. Neurosci. *31*, 9708–9722.

Blake, D.T., and Merzenich, M.M. (2002). Changes of AI receptive fields with sound density. J. Neurophysiol. *88*, 3409–3420.

Bregman, A.S. (1994). Auditory Scene Analysis: The Perceptual Organization of Sound (The MIT Press).

Brenner, N., Bialek, W., and de Ruyter van Steveninck, R. (2000). Adaptive rescaling maximizes information transmission. Neuron *26*, 695–702.

Brosch, M., and Schreiner, C.E. (1997). Time course of forward masking tuning curves in cat primary auditory cortex. J. Neurophysiol. *77*, 923–943.

Brosch, M., and Schreiner, C.E. (2000). Sequence sensitivity of neurons in cat primary auditory cortex. Cereb. Cortex *10*, 1155–1167.

Brosch, M., Schulz, A., and Scheich, H. (1999). Processing of sound sequences in macaque auditory cortex: response enhancement. J. Neurophysiol. *82*, 1542–1559.

Calford, M.B., and Semple, M.N. (1995). Monaural inhibition in cat auditory cortex. J. Neurophysiol. *73*, 1876–1891.

Carandini, M., Heeger, D.J., and Movshon, J.A. (1997). Linearity and normalization in simple cells of the macaque primary visual cortex. J. Neurosci. *17*, 8621–8644.

Chornoboy, E.S., Schramm, L.P., and Karr, A.F. (1988). Maximum likelihood identification of neural point process systems. Biol. Cybern. *59*, 265–275.

Christianson, G.B., Sahani, M., and Linden, J.F. (2008). The consequences of response nonlinearities for interpretation of spectrotemporal receptive fields. J. Neurosci. *28*, 446–455.

Creutzfeldt, O., Hellweg, F.C., and Schreiner, C. (1980). Thalamocortical transformation of responses to complex auditory stimuli. Exp. Brain Res. *39*, 87–104.

David, S.V., and Shamma, S.A. (2013). Integration over multiple timescales in primary auditory cortex. J. Neurosci. *33*, 19154–19166.

David, S.V., Fritz, J.B., and Shamma, S.A. (2012). Task reward structure shapes rapid receptive field plasticity in auditory cortex. Proc. Natl. Acad. Sci. USA *109*, 2144–2149.

deCharms, R.C., Blake, D.T., and Merzenich, M.M. (1998). Optimizing sound features for cortical neurons. Science *280*, 1439–1443.

Depireux, D.A., Simon, J.Z., Klein, D.J., and Shamma, S.A. (2001). Spectro-temporal response field characterization with dynamic ripples in ferret primary auditory cortex. J. Neurophysiol. *85*, 1220–1234.

Eggermont, J.J. (1993). Wiener and Volterra analyses applied to the auditory system. Hear. Res. *66*, 177–201.

Eggermont, J.J., Johannesma, P.M., and Aertsen, A.M. (1983). Reverse-correlation methods in auditory research. Q. Rev. Biophys. *16*, 341–414.

Egorova, M., and Ehret, G. (2008). Tonotopy and inhibition in the midbrain inferior colliculus shape spectral resolution of sounds in neural critical bands. Eur. J. Neurosci. *28*, 675–692.

Egorova, M., Vartanyan, I., and Ehret, G. (2006). Frequency response areas of mouse inferior colliculus neurons: II. Critical bands. Neuroreport *17*, 1783–1786.

Escabi, M.A., and Schreiner, C.E. (2002). Nonlinear spectrotemporal sound analysis by neurons in the auditory midbrain. J. Neurosci. *22*, 4114–4131.

Fairhall, A.L., Lewen, G.D., Bialek, W., and de Ruyter Van Steveninck, R.R. (2001). Efficiency and ambiguity in an adaptive neural code. Nature *412*, 787–792.

Fritz, J., Shamma, S., Elhilali, M., and Klein, D. (2003). Rapid task-related plasticity of spectrotemporal receptive fields in primary auditory cortex. Nat. Neurosci. *6*, 1216–1223.

Fritz, J.B., Elhilali, M., and Shamma, S.A. (2007). Adaptive changes in cortical receptive fields induced by attention to complex sounds. J. Neurophysiol. *98*, 2337–2346.

Gill, P., Zhang, J., Woolley, S.M.N., Fremouw, T., and Theunissen, F.E. (2006). Sound representation methods for spectro-temporal receptive field estimation. J. Comput. Neurosci. *21*, 5–20.

Gourévitch, B., Noreña, A., Shaw, G., and Eggermont, J.J. (2009). Spectrotemporal receptive fields in anesthetized cat primary auditory cortex are context dependent. Cereb. Cortex *19*, 1448–1461.

Guo, W., Chambers, A.R., Darrow, K.N., Hancock, K.E., Shinn-Cunningham, B.G., and Polley, D.B. (2012). Robustness of cortical topography across fields, laminae, anesthetic states, and neurophysiological signal types. J. Neurosci. *32*, 9159–9172.

Hackett, T.A., Barkat, T.R., O'Brien, B.M.J., Hensch, T.K., and Polley, D.B. (2011). Linking topography to tonotopy in the mouse auditory thalamocortical circuit. J. Neurosci. *31*, 2983–2995.

Heeger, D.J. (1992). Normalization of cell responses in cat striate cortex. Vis. Neurosci. *9*, 181–197.

Issa, J.B., Haeffele, B.D., Agarwal, A., Bergles, D.E., Young, E.D., and Yue, D.T. (2014). Multiscale optical Ca2+ imaging of tonal organization in mouse auditory cortex. Neuron *83*, 944–959.

Jesteadt, W., Bacon, S.P., and Lehman, J.R. (1982). Forward masking as a function of frequency, masker level, and signal delay. J. Acoust. Soc. Am. *71*, 950–962.

Kadia, S.C., and Wang, X. (2003). Spectral integration in A1 of awake primates: neurons with single- and multipeaked tuning characteristics. J. Neurophysiol. *89*, 1603–1622.

Kaur, S., Lazar, R., and Metherate, R. (2004). Intracortical pathways determine breadth of subthreshold frequency receptive fields in primary auditory cortex. J. Neurophysiol. *91*, 2551–2567.

Lee, C.C., Imaizumi, K., Schreiner, C.E., and Winer, J.A. (2004). Concurrent tonotopic processing streams in auditory cortex. Cereb. Cortex *14*, 441–451.

Linden, J.F., Liu, R.C., Sahani, M., Schreiner, C.E., and Merzenich, M.M. (2003). Spectrotemporal structure of receptive fields in areas AI and AAF of mouse auditory cortex. J. Neurophysiol. *90*, 2660–2675.

Machens, C.K., Wehr, M.S., and Zador, A.M. (2004). Linearity of cortical receptive fields measured with natural sounds. J. Neurosci. *24*, 1089–1100.

Malmierca, M.S., Izquierdo, M.A., Cristaudo, S., Hernández, O., Pérez-González, D., Covey, E., and Oliver, D.L. (2008). A discontinuous tonotopic organization in the inferior colliculus of the rat. J. Neurosci. *28*, 4767–4776.

Maravall, M., Petersen, R.S., Fairhall, A.L., Arabzadeh, E., and Diamond, M.E. (2007). Shifts in coding properties and maintenance of information transmission during adaptation in barrel cortex. PLoS Biol. *5*, e19.

Maravall, M., Alenda, A., Bale, M.R., and Petersen, R.S. (2013). Transformation of adaptation and gain rescaling along the whisker sensory pathway. PLoS ONE *8*, e82418.

Mease, R.A., Famulare, M., Gjorgjieva, J., Moody, W.J., and Fairhall, A.L. (2013). Emergence of adaptive computation by single neurons in the developing cortex. J. Neurosci. *33*, 12154–12170.

Mesgarani, N., David, S.V., Fritz, J.B., and Shamma, S.A. (2014). Mechanisms of noise robust representation of speech in primary auditory cortex. Proc. Natl. Acad. Sci. USA *111*, 6792–6797.

Metherate, R., Kaur, S., Kawai, H., Lazar, R., Liang, K., and Rose, H.J. (2005). Spectral integration in auditory cortex: mechanisms and modulation. Hear. Res. *206*, 146–158.

Mill, R., Coath, M., Wennekers, T., and Denham, S.L. (2011). A neurocomputational model of stimulus-specific adaptation to oddball and Markov sequences. PLoS Comput. Biol. *7*, e1002117.

Miller, L.M., Escabí, M.A., Read, H.L., and Schreiner, C.E. (2002). Spectrotemporal receptive fields in the lemniscal auditory thalamus and cortex. J. Neurophysiol. *87*, 516–527.

Nelken, I. (2014). Stimulus-specific adaptation and deviance detection in the auditory system: experiments and models. Biol. Cybern. *108*, 655–663.

Nelken, I., Prut, Y., Vaadia, E., and Abeles, M. (1994a). Population responses to multifrequency sounds in the cat auditory cortex: one- and two-parameter families of sounds. Hear. Res. *72*, 206–222.

Nelken, I., Prut, Y., Vaddia, E., and Abeles, M. (1994b). Population responses to multifrequency sounds in the cat auditory cortex: four-tone complexes. Hear. Res. *72*, 223–236.

Nelken, I., Kim, P.J., and Young, E.D. (1997). Linear and nonlinear spectral integration in type IV neurons of the dorsal cochlear nucleus. II. Predicting responses with the use of nonlinear models. J. Neurophysiol. *78*, 800–811.

Olsen, J.F., and Suga, N. (1991). Combination-sensitive neurons in the medial geniculate body of the mustached bat: encoding of relative velocity information. J. Neurophysiol. *65*, 1254–1274.

Paninski, L., Pillow, J., and Lewi, J. (2007). Statistical models for neural encoding, decoding, and optimal stimulus design. Prog. Brain Res. *165*, 493–507.

Park, I., Archer, E., Priebe, N.J., and Pillow, J.W. (2013). Spectral methods for neural characterization using generalized quadratic models. Adv. Neural Inf. Process. Syst. *26*, 2454–2462.

Pienkowski, M., and Eggermont, J.J. (2010). Nonlinear cross-frequency interactions in primary auditory cortex spectrotemporal receptive fields: a Wiener-Volterra analysis. J. Comput. Neurosci. *28*, 285–303.

Pienkowski, M., Shaw, G., and Eggermont, J.J. (2009). Wiener-Volterra characterization of neurons in primary auditory cortex using poisson-distributed impulse train inputs. J. Neurophysiol. *101*, 3031–3041.

Rabinowitz, N.C., Willmore, B.D.B., Schnupp, J.W.H., and King, A.J. (2011). Contrast gain control in auditory cortex. Neuron *70*, 1178–1191.

Rabinowitz, N.C., Willmore, B.D.B., Schnupp, J.W.H., and King, A.J. (2012). Spectrotemporal contrast kernels for neurons in primary auditory cortex. J. Neurosci. *32*, 11271–11284.

Rotman, Y., Bar-Yosef, O., and Nelken, I. (2001). Relating cluster and population responses to natural sounds and tonal stimuli in cat primary auditory cortex. Hear. Res. *152*, 110–127.

Sadagopan, S., and Wang, X. (2009). Nonlinear spectrotemporal interactions underlying selectivity for complex sounds in auditory cortex. J. Neurosci. *29*, 11192–11202.

Sahani, M., and Linden, J.F. (2003). How linear are auditory cortical responses? In Advances in Neural Information Processing Systems, S. Becker, S. Thrun, and K. Obermayer, eds. (Cambridge, MA: MIT Press), pp. 109–116.

Schinkel-Bielefeld, N., David, S.V., Shamma, S.A., and Butts, D.A. (2012). Inferring the role of inhibition in auditory processing of complex natural stimuli. J. Neurophysiol. *107*, 3296–3307.

Scholl, B., Gao, X., and Wehr, M. (2010). Nonoverlapping sets of synapses drive on responses and off responses in auditory cortex. Neuron *65*, 412–421.

Schreiner, C.E., and Langner, G. (1997). Laminar fine structure of frequency organization in auditory midbrain. Nature *388*, 383–386.

Schwartz, O., and Simoncelli, E.P. (2001). Natural signal statistics and sensory gain control. Nat. Neurosci. *4*, 819–825.

Schwartz, O., Pillow, J.W., Rust, N.C., and Simoncelli, E.P. (2006). Spike-triggered neural characterization. J. Vis. *6*, 484–507.

Shamma, S.A., Fleshman, J.W., Wiser, P.R., and Versnel, H. (1993). Organization of response areas in ferret primary auditory cortex. J. Neurophysiol. *69*, 367–383.

Shamma, S.A., Elhilali, M., and Micheyl, C. (2011). Temporal coherence and attention in auditory scene analysis. Trends Neurosci. *34*, 114–123.

Sharpee, T.O. (2013). Computational identification of receptive fields. Annu. Rev. Neurosci. *36*, 103–120.

Stiebler, I., Neulist, R., Fichtel, I., and Ehret, G. (1997). The auditory cortex of the house mouse: left-right differences, tonotopic organization and quantitative analysis of frequency representation. J. Comp. Physiol. A Neuroethol. Sens. Neural Behav. Physiol. *181*, 559–571.

Suga, N., O'Neill, W.E., and Manabe, T. (1979). Harmonic-sensitive neurons in the auditory cortex of the mustache bat. Science *203*, 270–274.

Suga, N., O'Neill, W.E., Kujirai, K., and Manabe, T. (1983). Specificity of combination-sensitive neurons for processing of complex biosonar signals in auditory cortex of the mustached bat. J. Neurophysiol. *49*, 1573–1626.

Sutter, M.L., Schreiner, C.E., McLean, M., O'connor, K.N., and Loftus, W.C. (1999). Organization of inhibitory frequency receptive fields in cat primary auditory cortex. J. Neurophysiol. *82*, 2358–2371.

Ulanovsky, N., Las, L., and Nelken, I. (2003). Processing of low-probability sounds by cortical neurons. Nat. Neurosci. *6*, 391–398.

Ulanovsky, N., Las, L., Farkas, D., and Nelken, I. (2004). Multiple time scales of adaptation in auditory cortex neurons. J. Neurosci. *24*, 10440–10453.

Valentine, P.A., and Eggermont, J.J. (2004). Stimulus dependence of spectro-temporal receptive fields in cat primary auditory cortex. Hear. Res. *196*, 119–133.

Walton, J.P., Frisina, R.D., and Meierhans, L.R. (1995). Sensorineural hearing loss alters recovery from short-term adaptation in the C57BL/6 mouse. Hear. Res. *88*, 19–26.

Wehr, M., and Zador, A.M. (2005). Synaptic mechanisms of forward suppression in rat auditory cortex. Neuron *47*, 437–445.

Wenstrup, J.J. (1999). Frequency organization and responses to complex sounds in the medial geniculate body of the mustached bat. J. Neurophysiol. *82*, 2528–2544.

Woolley, S.M.N., Fremouw, T.E., Hsu, A., and Theunissen, F.E. (2005). Tuning for spectro-temporal modulations as a mechanism for auditory discrimination of natural sounds. Nat. Neurosci. *8*, 1371–1379.

Young, E.D., and Calhoun, B.M. (2005). Nonlinear modeling of auditory-nerve rate responses to wideband stimuli. J. Neurophysiol. *94*, 4441–4454.

Yu, J.J., and Young, E.D. (2000). Linear and nonlinear pathways of spectral information transmission in the cochlear nucleus. Proc. Natl. Acad. Sci. USA *97*, 11780–11786.

Neuron, Volume *91*

Supplemental Information

Input-Specific Gain Modulation by Local Sensory Context Shapes Cortical and Thalamic Responses to Complex Sounds

Ross S. Williamson, Misha B. Ahrens, Jennifer F. Linden, and Maneesh Sahani

Input-specific Gain Modulation by Local Sensory Context Shapes Cortical and Thalamic Responses to Complex Sounds

Supplementary Information

Ross S. Williamson, Misha B. Ahrens, Jennifer F. Linden, and Maneesh Sahani

Supplementary Figures

S1 Predictive performance of separable models (related to Fig. 2a of the main text) 2
S2 The dual-CGF model (related to Fig. 2 of the main text) 3
S3 CGF model performance compared to performance of quadratic models (related to Fig. 2 of the main text) 5
S4 Comparing PRFs and STRFs (related to Fig. 4a of the main text) 6
S5 Contribution of cell-specific CGFs to predictions (related to Fig. 6 of the main text) 7

Supplementary Experimental Procedures

1 Details of experimental procedures (related to Experimental Procedures) 8
1.1 Animals 8
1.2 Surgical procedures 8
1.3 Recording procedures 8
1.4 Stimuli 9
1.5 Data analysis and modelling 9
2 Analysis of input gain specificity (related to Fig. 1 of the main text) 10
3 Evaluating predictive power of STRF and CGF models (related to Fig. 2 of the main text) . . . 10
4 Fitting 1-D and 2-D quadratic models (related to Supplementary Fig. S3) 13
5 Predicting the STRF for a DRC stimulus from the PRF and CGF (related to Supplementary Fig. S4) 14

Supplementary Figures

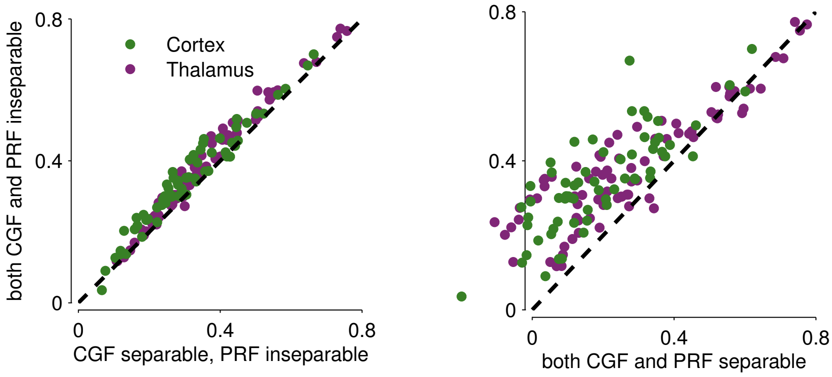

Supplementary Figure S1: Predictive performance of separable models (related to Fig. 2a of the main text)

The generalisation performance (measured as a fraction of predictable response variance accurately predicted during cross-validation) of a model with both CGF and PRF inseparable (ordinate) as in the main text, compared to models where either the CGF alone (left abscissa) or both CGF and PRF (right abscissa) are constrained to be separable. The inseparable model provides a better fit to the contextual input-specific gain modulation than either alternative. In particular, it generalises more accurately despite the many more degrees of freedom that are contained within the two inseparable weight matrices than in their separable equivalents. These added degrees of freedom should allow for greater overfitting in the inseparable model, and thus the size of the true generalisation advantage may be underestimated here.

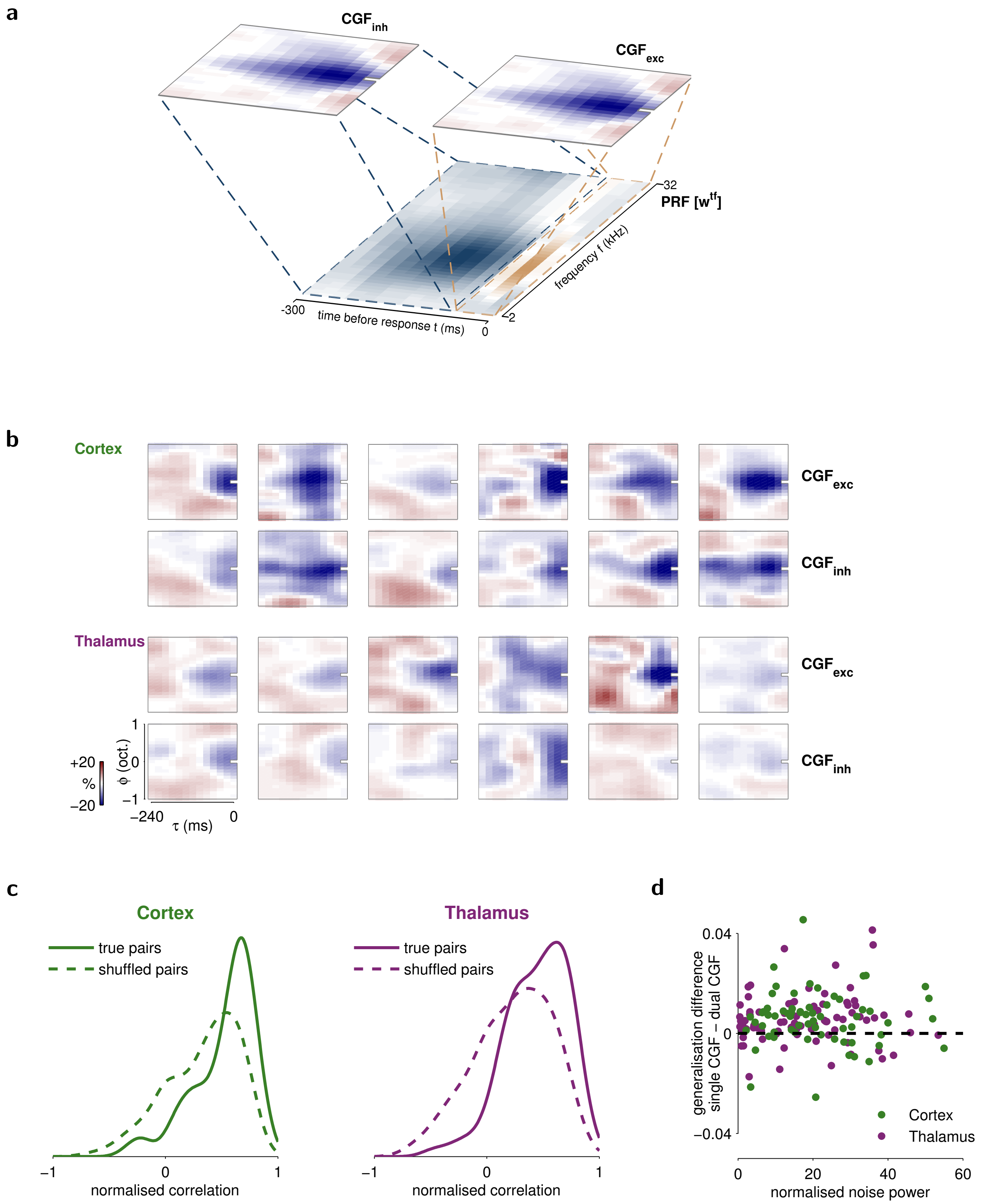

Supplementary Figure S2: Dual-CGF model (related to Fig. 2 of the main text)

(Description on following page)

The model employed in the main text assumed that the form of contextual input-specific gain dependence, embodied in the CGF, was the same at each point within the PRF. We tested this assumption by studying "dual-CGF" models in which the CGF was allowed to differ between two different regions of the PRF. Results are shown here for a model in which the two regions were defined by the timing of PRF excitation and inhibition. (**a**) Illustration of the model. Each PRF (identified by using the standard "single-CGF" model) was divided into two regions along its temporal axis by identifying the point of transition from net excitation to net inhibition. A model was then re-fit with two different CGFs: $\mathsf{CGF}_{\mathsf{exc}}$ applied to the short-latency weights (with net excitation) and $\mathsf{CGF}_{\mathsf{inh}}$ applied to longer-latency weights (with dominant inhibition). (**b**) Example dual CGFs fit to six recordings each in cortex (upper panels) and thalamus (lower panels). Pairs of CGFs look broadly similar, supporting the hypothesis that the form of contextual gain dependence does not differ substantially between the two PRF regions. (**c**) Distribution of correlation coefficients between CGF weights for $(\mathsf{CGF}_{\mathsf{ecx}}, \mathsf{CGF}_{\mathsf{inh}})$ pairs fit to the same recordings (solid lines) compared to the distribution obtained for pairs fit to different recordings (dashed lines). The true pairs are more similar than shuffled ones. (**d**) Difference in generalisation performance (measured as a fraction of predictable response variance accurately predicted during cross-validation) of the single- and dual-CGF models, plotted as a function of recording variability (normalised noise power). The single-CGF model generalises more accurately overall, and even for recordings with low variability, suggesting that the added degrees of freedom in the dual-CGF models lead to overfitting and do not help model the contextual input-specific gain effect more closely. Similar results were obtained when the two CGFs applied to the low-frequency half and high-frequency half of the PRF (not shown). Taken together, these results support the interpretation that a similar pattern of input-specific gain modulation acts upon different regions of the receptive field.

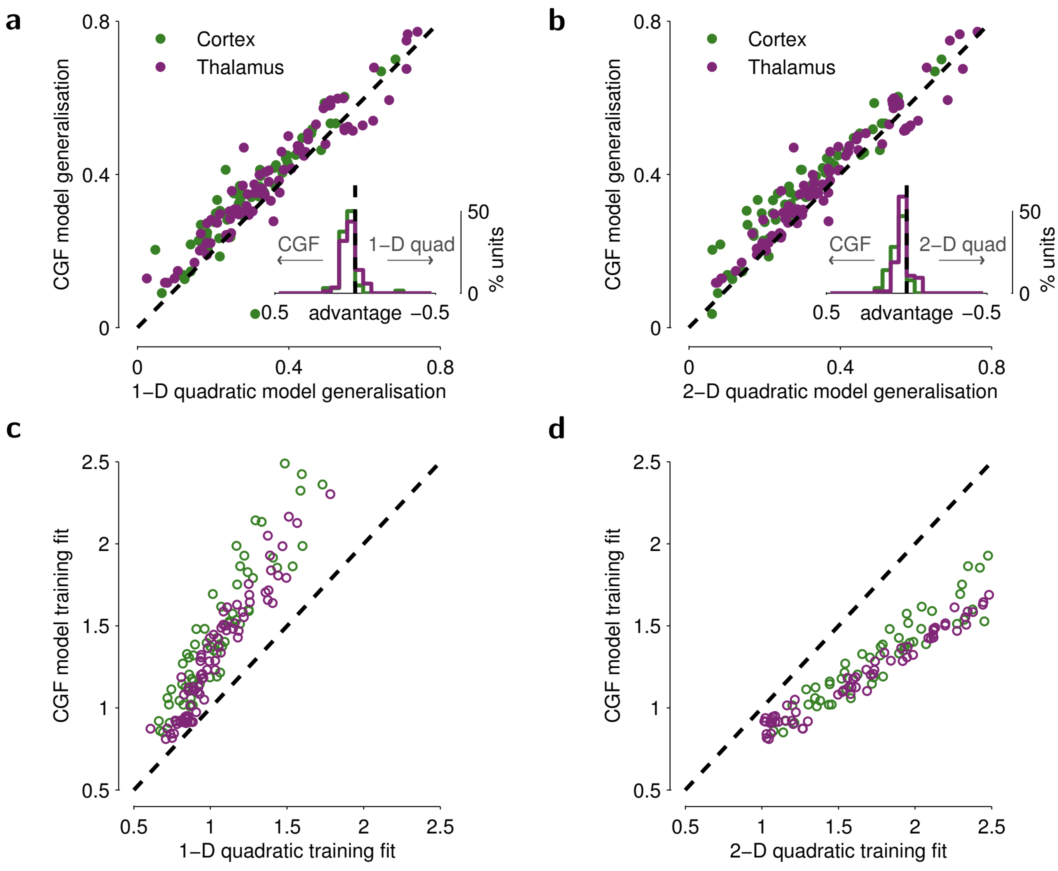

Supplementary Figure S3: CGF model performance compared to performance of quadratic models (related to Fig. 2 of the main text)

(**a**,**b**) Neither a one-dimensional nor a two-dimensional quadratic model generalises as well as the CGF-based context model in cross-validation. Conventions as in Fig. 2a of the main text. Both generalisation performance and training fit (c,d) are normalised by the estimated signal power of the recording. (**c**) The one-dimensional quadratic model accounted for a smaller part of the *training data* signal power than did the CGF model, indicating that the degrees of freedom available within the outer-product Volterra form, while more than twice as numerous than the degrees of freedom of the CGF model (720 versus 324), are nevertheless not as suitably directed to capture the stimulus-dependent neural response even in training data. (**d**) The two-dimensional model, with more than four times as many parameters as the CGF model, did achieve a better fit to the training data — but still generalised more poorly than the CGF model even after regularisation (b), suggesting that the improvement resulted from overfitting.

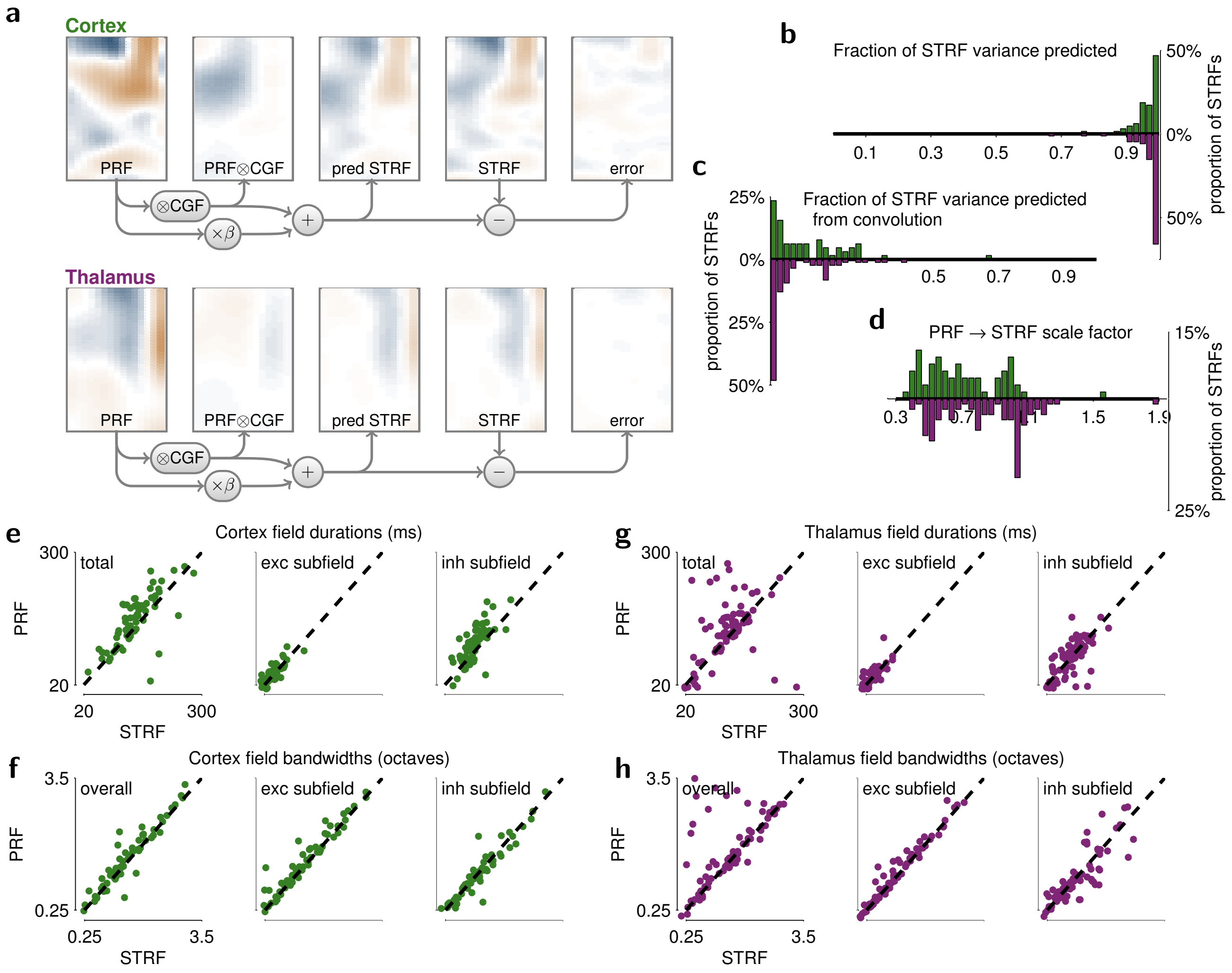

Supplementary Figure S4: Comparing PRFs and STRFs (related to Fig. 4a of the main text)

(**a**–**d**) STRFs estimated using the independent DRC stimulus agree with predictions derived from the estimated CGF model parameters. As derived in Supplementary Experimental Procedures 5, the predicted STRF is formed by combining a copy of the PRF scaled by a factor β with the result of convolving the PRF by the CGF, scaled by the average stimulus strength $\bar{s}$ (a). All panels for each of the two example recordings are shown at the same color scale. The error between the predictions and the measured STRFs is small. Across the populations, the majority of variance in the measured STRFs can be accurately predicted from the CGF model (b). The convolution term generally contributes less than a third of the predicted variance (c), suggesting that changes in STRF shape arising through nonlinear contextual interactions are significant, but contained when using an independent DRC stimulus. More structured or natural stimuli are likely to produce a larger shape difference (Christianson et al., 2008). The scale factors applied to the PRF to form the prediction are generally less than 1 (d), indicating that on average the PRF weights are stronger than the STRF weights. This observation is consistent with the finding that CGF weights are predominantly suppressive.

(**e**–**h**) Comparison of receptive-field extents estimated by the PRF and STRF. Field durations and bandwidths were estimated for the excitatory and inhibitory subfields and for the overall linearly-weighted receptive field using the definitions employed by Linden et al. (2003). Estimated extents were quantised by the stimulus spectrotemporal resolution at 20ms in time and 1/12 octave in frequency, however values were jittered within this quantisation window to aide visualisation of the density of data points. PRF and STRF structure appears broadly similar, although inhibitory subfields tend to be of longer duration in the PRF.

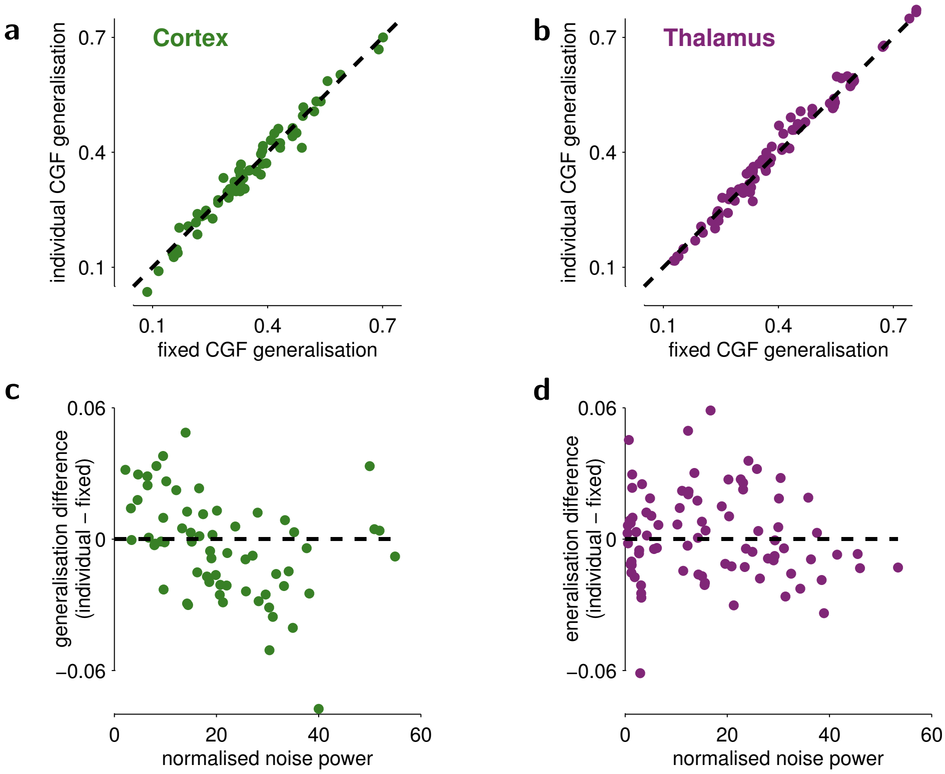

Supplementary Figure S5: Contribution of cell-specific CGFs to predictions (related to Fig. 6 of the main text)

The generalisation performance (measured as the fraction of predictable response variance accurately predicted during cross-validation) of models with an individual CGF fit to each recording as described in the main text, compared to a model in which the CGFs for all recordings were fixed either (**a**,**c**) to the mean CGF for the corresponding cortical field (A1 or AAF) or (**b**,**d**) to the mean CGF in the thalamus (where the subregion means were indistinguishable). Performance of both models was broadly similar across each population, suggesting that the common field-specific contextual gain effects identified in Fig. 7 of the main text play a major role in shaping all gain-sensitive responses. However, the effective number of degrees of freedom available to the population of models with individual CGFs fit to each recording is many times greater than the number of degrees of freedom for models with a fixed CGF for all recordings. The additional degrees of freedom in the individual CGFs will allow for overfitting, and should thus tend to reduce generalisation performance unless these degrees of freedom are also important to modelling the true response. This effect is reflected in a trend (c,d) for individual-CGF models to generalise more poorly than those with a fixed CGF when recordings are more variable (higher normalised noise power), as these are the cases where overfitting is likely to play a more significant role (see also Fig. 2 of the main text). Notably, however, for the recordings with the most reliable stimulus-dependent signal (lowest normalised noise power), generalisation performance was usually better for models with cell-specific CGFs than for models with a fixed CGF, at least in the auditory cortex (c). Hence, we conclude that the individual variation in CGFs within a field does also contribute to shaping sensory responses, but that we did not have the statistical power in the present study to quantify the precise extent of this contribution.

Supplementary Experimental Procedures

1 Details of experimental procedures (related to Experimental Procedures)

1.1 Animals

Twelve adult male CBA/CaJ mice (6–15 weeks old) were used for cortical experiments, and six adult male CBA/Ca mice (6–8 weeks old) for thalamic experiments. These mice are the same CBA/Ca inbred strain, obtained from different vendors; Jackson Labs versus Harlan or Charles River UK. Mice were maintained in standard cages and under standard mouse housing conditions.

1.2 Surgical procedures

Surgical procedures for cortical and thalamic recording experiments were similar to those described previously in Linden et al. (2003). Mice were anaesthetised with ketamine and medetomidine. After an initial intraperitoneal bolus injection of anaesthetic, a cannula was placed into the animal's peritoneal cavity so that maintenance boluses or continuous infusion of anaesthetic could be provided. Dexamethasone was administered to control brain oedema, atropine to minimise bronchial secretions, and Ringer's solution to ensure adequate hydration. The animal was kept on a homeothermic blanket (Harvard Instruments) to ensure that the body temperature was maintained at approximately $37.5 \pm 0.5°C$ (monitored via a rectal probe). Once fully anaesthetised and prepared for surgery, the animal was placed onto a bite bar in order to immobilise its head, after which the skin was transected along the midline to expose the skull.

For cortical experiments, a small craniotomy was performed on the left-hand side of the skull, to expose a region bordered rostrally by the lambdoid suture, caudally and ventro-laterally by the squamosal suture, and dorso-medially by the temporal ridge. Cortical areas A1 and AAF were identified physiologically by reversal of the tonotopic gradient as described previously in Linden et al. (2003).

For thalamic experiments, a craniotomy approximately 2.5 mm in diameter, centred 2.75 mm lateral to midline and 2.75 mm caudal to bregma, was performed on the right-hand side of the skull, enabling vertical access to the thalamus. Thalamic recording sites were localised to vMGB or mMGB histologically, using procedures similar to those described by Anderson et al. (2009) and Anderson and Linden (2011). Electrolytic lesions were created by passing current through the desired electrode on the array (5μA for 7 secs). Such lesions were typically created at the most medial and lateral electrodes on the array that yielded auditory activity. Lesions were replicated at both the top and bottom of the electrode track. Ideally, this procedure yielded four lesions (two at the top of the track, and two at the bottom), bracketing the area over which auditory activity had been recorded. This placement of lesions allowed for estimation of shrinkage and histological reconstruction of most recording sites.

Once lesioning was complete, animals were euthanised with sodium pentobarbital and perfused transcardially with 4% paraformaldehyde in 0.1 M phosphate buffer. Following perfusion, the brain was removed and placed in the paraformaldehyde solution for 1–2 days. Blocks containing the full auditory thalamus were then cut into $50\,\mu$m slices using a vibratome. The sections were then stained for the metabolic marker cytochrome oxidase (CYO), which delineates auditory thalamic subdivisions. Slides were incubated for 3–7 hours at 37°C in a solution containing 20 mg of diaminobenzidine hydrochloride in 10 ml of distilled water and 30 mg of cytochrome c with 3 g of sucrose in 30 ml of 0.1 M phosphate buffer.

Electrolytic lesions were visualised in the stained brain sections using a Zeiss AxioPlan 2 Imaging microscope (magnification x25–x200). The position of each neuron was assigned to the appropriate subdivision as defined by the CYO distribution. Recording sites for which localisation was ambiguous were not included in the subdivided datasets.

1.3 Recording procedures

For cortical experiments, extracellular recordings were made using epoxylite-coated tungsten electrodes (FHC Inc.; 1–4 MΩ impedance). These were introduced into the left auditory cortex in penetrations orthogonal to the cortical surface. Recordings targeted the thalamorecipient layers III/IV (Smith and Populin, 2001) by cortical depth (350–600 μm below the dural surface), and were obtained using clicks and frequency sweeps as search

stimuli. Cortical areas A1 and AAF were identified physiologically by reversal of the tonotopic gradient as described in Linden et al. (2003).

For thalamic experiments, extracellular recordings were made across all thalamic subdivisions using custom-made linear arrays consisting of eight tungsten electrodes (World Precision Instruments; impedance typically 1-2 MΩ). The array was placed perpendicular to the midline with the first penetration targeting a position approximately 2 mm from midline, 3 mm from bregma, and 2200 μm below the cortical surface, as this position was deemed most likely to yield responses from all three major thalamic subdivisions (Anderson and Linden, 2011). Neurons responsive to auditory stimuli were located by their responses to clicks. Once an auditory response had been established (typically at depths of about 2900 μm), further sites were located by progressing the electrode 100 μm at a time, until auditory activity was lost. Histological delineation was carried out as described above to identify subdivision locations for all thalamic recordings, and recording sites for which localisation was ambiguous were not included in the subdivided datasets.

Cortical and thalamic recordings were analysed off-line using Bayesian spike-sorting techniques (Sahani, 1999; Lewicki, 1998) to extract responses from either small clusters of neurons or single units. We used automated clustering criteria to quantify single-unit isolation. Using Bayesian criteria requiring >95% probability of single-unit isolation, only a minority of the recordings in cortex and thalamus were judged to be definitively single units; therefore we conservatively assume here that many recordings were from local multiunits.

1.4 Stimuli

To obtain an initial characterisation of the frequency-intensity response area for each recording site, we used simple tonal stimuli consisting of 50 or 100 ms tone pulses, ramped up and down with 5 ms cosine gates. The frequency and intensity of each tone were varied pseudorandomly over a 2–32 kHz range of frequencies (in 1/12-octave steps) and a 0–70 dB SPL range of in intensities (in 5 dB increments). Other simple stimuli such as clicks, broadband noise, and frequency-modulated sweeps were also used to identify recording sites where auditory activity was present.

We then presented the 2–32 kHz dynamic random chord (DRC) stimulus described previously by Linden et al. (2003). This spectrotemporally rich stimulus is clocked such that every 20 ms a combination of 20 ms cosine-gated tone pulses with randomly chosen frequencies and intensities is generated. Centre frequencies of the tone pulses were chosen from 48 different possibilities (2–32 kHz in 1/12 octave steps). The number of tones that composed a chord was random, with an average density of two tone pulses per octave. The peak level of each pulse was chosen randomly from 10 different intensity levels, 5 dB SPL apart in the range 25–70 dB SPL. A single trial of the DRC stimulus lasted 60 seconds. Full presentation of the stimulus lasted for 20 minutes, allowing for 20 continuous trials. Cortical experiments also involved presentations of a 25–100 kHz version of the DRC stimulus, which was not used in thalamic experiments; cortical recordings using this "high-frequency" DRC stimulus were therefore not included in the analysis here.

1.5 Data analysis and modelling

We fit both linear STRF models and multilinear contextual input-specific gain models to the DRC-evoked neural responses. Estimation of the STRFs was carried out using the automatic smoothness determination algorithm (ASD) algorithm (Sahani and Linden, 2003a). Conceptually, this approach uses regularised linear regression with a smoothness constraint which is optimised separately for each recording.

The contextual input-specific gain model of equation 1 of the main text is bilinear, and was fit using the alternating least squares (ALS) approach of Ahrens et al. (2008). Each least-squares step was regularised using either the ASD-derived optimal spectrotemporal smoothing (for the PRF) or a fixed smoothing bandwidth of 40 ms and 1/6 octave (for the CGF). The fixed CGF smoothing was necessary to facilitate straighforward averaging and comparison of CGF properties across recordings. All PRFs and CGFs shown in this study were regularised in this way. However, training data performance measures in Fig. 2b of the main text and Fig. 2c of the main text were derived from unregularised fits.

Generalisation performance was assessed using ten-fold cross-validation, reserving a randomly distributed disjoint subset of one-tenth of the bins as the validation set for each of the ten repetitions.

2 Analysis of input gain specificity (related to Fig. 1 of the main text)

To illustrate the locality of the input gain effects in an example unit, we chose two spectrotemporal positions over an octave apart within the responsive region of the unit's STRF (Fig. 1f of the main text): input 1 had a temporal offset of 20 ms and a centre frequency of 31.09 kHz ($j_1 = 2; k_1 = 48$) and input 2 had a temporal offset of 40 ms and centre frequency of 13.85 kHz ($j_2 = 3; k_2 = 34$). For each input, we first calculated the average number of spikes observed when the sound amplitude at that point in the STRF took on each of the 11 possible values (including 0) $s_0 \ldots s_{10}$. That is, for each input $p = 1, 2$ and level index $l = 0 \ldots 10$, we averaged the responses $r(t)$ at all times at which the DRC stimulus took on the level s_l at the pth input location:

$$\bar{r}_p(s_l) = \langle r(t) \rangle_{t\,:\,s(t-j_p,k_p)=s_l} \,.$$

The slope of the linear relationship between $\bar{r}_p(s_l)$ and s_l is essentially an unregularised estimate of the corresponding STRF weight (Fig. 1g–i of the main text, grey open circles and dashed lines), that is the gain with which the unit responds to this particular input.

We then asked how this response gain was affected by local and remote context. We fit the context model to this unit, and computed the predicted gain modulation $G(i, k)$ at each time i and frequency k in the stimulus. (Similar values are illustrated in Fig. 3 of the main text.) To avoid risk of overfitting, for this analysis we estimated the gain modulations using a cross-validation scheme: that is, the gain at time i was estimated using a CGF derived from a cross-validation fold in which the training data did not include time i. We divided the values $G(i, k)$ into three equal-sized quantile sets — $\mathcal{Q}_{\text{low}}$, $\mathcal{Q}_{\text{mid}}$ and $\mathcal{Q}_{\text{high}}$ — and repeated the averages, now selecting for times when either the local or the remote context fell in a specific quantile. That is, for input $p = 1, 2$ and context $q = 1, 2$ we found

$$\bar{r}_{p,q\ \text{low}}(s_l) = \langle r(t) \rangle_{t\,:\,s(t-j_p,k_p)=s_l \text{and} G(t-j_q,k_q)\in\mathcal{Q}_{\text{low}}} \,,$$

along with similar averages for $\mathcal{Q}_{\text{mid}}$ and $\mathcal{Q}_{\text{high}}$. These values, along with the corresponding linear relationships, are shown in Fig. 1g–j of the main text. As described in Results, we then tested hypotheses regarding changes in the slopes of these linear relationships. The significance of the changes in slope was assessed by comparing each observed difference in slopes to a simulated null distribution of differences constructed by permuting the time indices of the predicted gain values $G(i, k)$ and then repeating the analysis. The p-values quoted are the proportion of 1000 simulations on which a larger difference in slopes was observed after permutation.

Note that even if context had no effect on input gain, our analysis could generate a change in intercept in the $\bar{r}_{p,q}$ relationships. This is because the CGF applied around (say) location 1 is not orthogonal to the local part of the STRF, and so the local predicted gain will be correlated with the linear input integrated over that local region. Thus, restricting to times when $G(t - j_1, k_1) \in \mathcal{Q}_{\text{low}}$ implicitly selects times when the local linear input around location 1 is low. However, in a linear model this effect must be additive and independent of the level at input 1 (and of that at input 2, given that it is an octave away). Thus it would lead to a constant offset in the linear relationships, not to a change in slope.

3 Evaluating predictive power of STRF and CGF models (related to Fig. 2 of the main text)

This section of Supplementary Experimental Procedures provides a more detailed explanation of the methods used in Fig. 2 of the main text for evaluating predictive power of STRF and CGF models. This approach was originally introduced by Sahani and Linden (2003b).

Intuitions underlying the approach

The variability of neural responses to a repeated stimulus leads to two difficulties in evaluating the predictive performance of a stimulus-response function model such as an STRF model or CGF model. First, the variability obscures the desired target for the model output. Perfect prediction of a noisy response is impossible, even in principle; moreover, since the true underlying relationship between stimulus and neural response is unknown, it is unclear what degree of partial prediction could possibly be expected. Second, noise introduces error into the estimation of the model parameters themselves (e.g., the STRF weights, or in the CGF model, the PRF and

CGF weights). Consequently, the estimated model parameters will inevitably differ from the "ideal parameters" that would have been obtained in the absence of noise, and response predictions from the estimated model will therefore understate the predictive performance that those ideal parameters might have achieved.

These difficulties are both manifest in a classical statistical measure of goodness-of-fit: the coefficient of determination, or r^2 statistic. This is the ratio between the reduction in variance achieved by a regression model (the total variance of the measured outputs minus the variance of the residuals) and the total variance of the measured outputs. The total variance of the outputs appearing in the denominator includes a contribution from the noise, and so an r^2 of 1 is an unrealistic target and the actual maximum achievable value of r^2 is unclear. Moreover, the reduction of variance obtained using the same data as were employed to to fit the model parameters (the "training data"), which is the factor that appears in the numerator of r^2, includes some "explanation" of noise due to the phenomenon of overfitting, where a chance partial correlation between the model inputs and the noise allows the model to fit these elements of the variability.

Following Sahani and Linden (2003b), we take an alternative approach in this study, compensating for the disadvantages of r^2 in three key analytic steps that overcome the confounding effects of neural response variability on model evaluation and model estimation. First, we derive an unbiased estimate of the total *predictable* (stimulus-dependent) component of the variance in the neural response (see the section on maximum predictable power below). Second, we assess model predictions relative to this noise-independent standard, both on the training data used to estimate the model parameters (as for the r^2) and on test data not used to estimate the parameters, using the standard procedure of cross-validation, described below. For any single recording, the predictive performance of the estimated model on training data and on test data provide, respectively, over- and under-estimates of the predictive power of the version of the model with ideal parameters. When the trial-to-trial variability of the neural response is large, these estimates might bound the predictive power of the ideal version of the model extremely loosely. However, upper and lower estimates of predictive power for a population of similar neural recordings can be extrapolated with respect to the degree of variability in each recording, to obtain an estimate of the fraction of predictable power that would have been explainable given an idealised recording from the same population that exhibited no trial-to-trial variability. In the third and final stage of the analysis, we perform such an extrapolation, to quantify the extent to which either STRF or CGF models can account for auditory cortical and thalamic responses to dynamic random chord stimuli in the zero-noise limit.

Maximum predictable power ("signal power")

In our experiments, a DRC stimulus comprising T random chords was repeated N (= 20) times for each recording. The resulting spike-trains were binned (in 20 ms bins) to yield a set of N response vectors $\{\mathbf{r}^{(n)}\}_{n=1}^{N}$ for each unit, with each response vector formed of T spike counts $(r_1^{(n)}, r_2^{(n)}, \ldots, r_T^{(n)})$. Our objective is to measure the performance of a predictive model in terms of the fraction of *response power* that it successfully predicts, where "power" is used here in the sense of average squared deviation from the mean over time: $P(\mathbf{r}) = \langle (r_t - \langle r_t \rangle)^2 \rangle$, with $\langle \cdot \rangle$ used to denote averages over time. As argued above, only some part of this total response power is predictable, even in principle; fortunately, the magnitude of this *signal power* can be estimated for each neuron by analysing the repeated responses to the same stimulus sequence. We provide here an intuitive derivation for the relevant estimator; see also Sahani and Linden (2003b).

The impossibility of perfect prediction results from the variability in the responses $\mathbf{r}^{(n)}$. To characterise this variability, we divide each response into a reliable and a variable component: $\mathbf{r}^{(n)} = \boldsymbol{\mu} + \boldsymbol{\eta}^{(n)}$, with the variable component $\boldsymbol{\eta}^{(n)}$ defined to have an expected value of zero in every time bin. From the point of view of a predictive model, $\boldsymbol{\eta}^{(n)}$ is unpredictable noise, and indeed we refer to it in the following as "noise" even though it may in fact reflect biologically meaningful but non-stimulus-locked activity. If we could average together an infinite set of responses to the same stimulus, we would obtain the "signal" part $\boldsymbol{\mu}$. This reflects the stimulus-driven response of the neuron under consideration, and is thus the only component that is predictable by a model of the cell's stimulus-response function. However, the average of a finite number of trial responses collected within experimental constraints retains a contribution from the noise, and thus the true signal response $\boldsymbol{\mu}$ cannot be determined. Nevertheless, it is possible to form an unbiased estimator of the power in that response, as follows.

First, the simple property of additivity of variances implies that $P(\mathbf{r}^{(n)}) \stackrel{\mathcal{E}}{=} P(\boldsymbol{\mu}) + \left\langle (\eta_t^{(n)})^2 \right\rangle$ (where the symbol $\stackrel{\mathcal{E}}{=}$ is used to represent "equal in expectation"—i.e., the equality may not hold on any trial, but the expected

values of the left- and right-hand sides are equal). This relationship depends only on the noise component having been defined to have zero expectation, and holds even if the variance or other property of the noise depends on the signal strength as would be expected for a Poisson noise process. We now construct two trial-averaged quantities, similar to the sum-of-squares terms used in the analysis of variance (ANOVA): the power of the average response, and the average power per response. Using $\bar{\cdot}$ to indicate trial averages:

$$P(\overline{\mathbf{r}^{(n)}}) \stackrel{\mathcal{E}}{=} P(\boldsymbol{\mu}) + P(\overline{\boldsymbol{\eta}^{(n)}}) \qquad \text{and} \qquad \overline{P(\mathbf{r}^{(n)})} \stackrel{\mathcal{E}}{=} P(\boldsymbol{\mu}) + \overline{P(\boldsymbol{\eta}^{(n)})}.$$

Assuming the noise in each trial is independent, although the noise in different time bins within a trial need not be, we have: $P(\overline{\boldsymbol{\eta}^{(n)}}) \stackrel{\mathcal{E}}{=} \overline{P(\boldsymbol{\eta}^{(n)})}/N$. Then solving these equations for $P(\boldsymbol{\mu})$ suggests the following estimator for the signal power:

$$\hat{P}(\boldsymbol{\mu}) = \frac{1}{N-1}\left(NP(\overline{\mathbf{r}^{(n)}}) - \overline{P(\mathbf{r}^{(n)})}\right). \tag{S1}$$

A similar estimator for the *noise power* is obtained by subtracting this expression from $\overline{P(\mathbf{r}^{(n)})}$. Both estimators are unbiased, provided only that the noise distribution has defined first and second moments and is independent between trials. Unlike the sum-of-squares terms encountered in an ANOVA, the signal power estimate is not a χ^2 variate even when the noise is normally distributed (indeed, it is not necessarily positive). However, since each of the power terms in Eq. S1 is the mean of at least T numbers, the central limit theorem suggests that $\hat{P}$ will be approximately normally distributed for recordings that are considerably longer than the time-scale of noise correlation (in the experiments considered here, $T = 3000$, equivalent to a duration of 60 s). Its variance is given by:

$$\mathcal{V}ar\left[\hat{P}\right] = \frac{4}{N}\left(\frac{1}{T^2}\boldsymbol{\mu}^{\mathsf{T}}\Sigma\boldsymbol{\mu} - \frac{2}{T}\mu\boldsymbol{\sigma}^{\mathsf{T}}\boldsymbol{\mu} + \mu\sigma\mu\right) + \frac{2}{N(N-1)}\left(\frac{1}{T^2}\mathsf{Tr}\left[\Sigma\Sigma\right] - \frac{2}{T}\boldsymbol{\sigma}^{\mathsf{T}}\boldsymbol{\sigma} + \sigma^2\right), \tag{S2}$$

where Σ is the $(T \times T)$ covariance matrix of the noise, $\boldsymbol{\sigma}$ is a vector formed by averaging each column of Σ, σ is the average of all the elements of Σ and μ is the time-average of the signal $\boldsymbol{\mu}$. Thus, $\mathcal{V}ar\left[\hat{P}\right]$ depends only on the first and second moments of the response distribution; substitution of data-derived estimates of these moments into Eq. S2 yields a standard error bar for the estimator.

In this way we have obtained an estimate $\hat{P}$ (along with corresponding uncertainty) of the maximum possible signal power that any model could accurately predict, having assumed neither a particular distribution nor short-time-scale independence in the noise. Essentially, this signal power is the *stimulus-dependent* power in the neural response, i.e., the part of the response that is, in principle, predictable from the stimulus alone. The signal power therefore provides an absolute yardstick against which the performance of any stimulus-response function model can be judged. If the model is correct, then it should predict all of the signal power in the neural responses for a given stimulus, regardless of the level of noise power.

Upper and lower estimates of model predictive power

The estimate of the signal power forms a reference against which to compare the magnitude of response power accurately predicted by a particular model. This model *predictive power* is not necessarily the power of the predicted response $\boldsymbol{\rho}$, since that prediction may be inaccurate. Instead, as in the numerator of the coefficient of determination, it is given by the difference between the power in the observed response $P(\mathbf{r})$ and the *error power* or power in the residuals $P(\mathbf{r} - \boldsymbol{\rho})$.

The magnitude of this predictive power will depend both on the parameters used for the model prediction, and on the stimulus used to compare prediction to measurement. We define the *true predictive power* of a particular class of model (such as the STRF model) to be the predictive power that would be achieved by the version of the model with "ideal" parameters (e.g., ideal STRF weights or coefficients), which maximise predictive power across all stimulus-response combinations of the type under study (e.g., responses to all possible random chord stimuli). This true predictive power cannot be determined from realistic volumes of experimental data; however, it is possible to obtain a pair of predictive power estimates that are likely to bracket its value, as explained below.

Model parameters (such as the weights or coefficients of the STRF) are commonly estimated by minimising

the mean squared error of the model prediction on the training data. By definition, these least-mean-squares (LMS) parameters produce model predictions for the training data that have minimum possible error, and therefore maximal predictive power. Of course, the resulting maximal value, the *training predictive power*, will inevitably include an element of overfitting to the training data, and so will overestimate the true predictive power of the model with ideal parameters (which would perform best on average for all possible stimulus-response combinations, not just the training data). More precisely, the expected value of the training predictive power of the LMS parameters is an upper bound on the predictive power of the model with ideal parameters. Thus, the measured training predictive power can be considered an *upper estimate* of the true predictive power of the model class.

We can also obtain a *lower estimate*, defined similarly, by empirically measuring the generalization performance of the model by cross-validation. Cross-validation is a standard statistical procedure (Duda and Hart, 1973), in which each data set is repeatedly divided into a "training" segment and a "test" segment (in this study, 9/10 and 1/10 of the full stimulus length, respectively). Model parameters are estimated using responses to the training segment alone; a test prediction is obtained by applying the model to the test segment; and the mean squared difference between this prediction and the observed response to the test segment is calculated. This procedure is repeated multiple times (here, 10 times), on each occasion using a different division of the data into training and test segments. The average of the multiple mean-squared-error figures obtained in this way is the *cross-validation error power*. The difference between this error power and the total response power in the recording is the *cross-validation predictive power*. Cross-validation provides an unbiased estimate of the average generalization performance of the fitted models (as obtained from the training fraction of the available data). Since these models are inevitably overfit to their training data, not the test data, the expected value of this cross-validation predictive power bounds the predictive power of the model with ideal parameters from below, and thereby provides the desired lower estimate of the true predictive power of the model class. These lower estimates may be tightened somewhat by optimising model parameters to improve generalisation performance, for example using the Bayesian smoothing and de-noising techniques applied here (Sahani and Linden, 2003a).

Population extrapolation to zero-noise limit

For any one recording of finite length, the true predictive power of the model class (i.e., the predictive power of the version of the model with ideal parameters) can only be bracketed between the upper and lower estimates defined above. The looseness of these estimates will depend on the variability or noise in the recording. For a recording with high trial-to-trial variability, the model parameters will be more strongly overfit to the noise in the training data. Thus we expect the training predictive power on such a recording to appear high relative to the signal power, and the cross-validation predictive power to appear low. Indeed, in very high-noise conditions, the model may primarily describe the stimulus-independent noisy part of the training data, and so the training predictive power might exceed the estimated signal power ($\hat{P}(\boldsymbol{\mu})$), while the cross-validation predictive power may fall below zero (that is, the predictions made by the model may be worse than a simple unchanging mean rate prediction). Thus, the estimates may not usefully constrain the predictive power measure for a particular recording.

However, for a population of recorded neurons that are relatively homogeneous, it is possible to tighten the estimates of model predictive power *for the population as a whole*, by normalising the upper and lower estimates of model predictive power by the signal power for each recording, plotting these normalised estimates as a function of noise power—also normalised by signal power—for each recording, and then extrapolating across the population to the theoretical zero noise level. *The upper and lower estimates of model predictive power in this zero-noise limit provide the desired noise-independent measure of model predictive performance.* This extrapolation is shown in Fig. 2 of the main text for both STRF and CGF models, and for the populations of auditory cortical and thalamic recordings.

4 Fitting 1-D and 2-D quadratic models (related to Supplementary Fig. S3)

We implemented one- and two-dimensional quadratic models to compare with the CGF model. Like the CGF model, these models are constrained parametrisations of the second-order spectrotemporal Volterra kernel; however, the low-dimensional constraint is not formulated in terms of input-specific contextual gain. Similar models were discussed by Park et al. (2013) in the context of an approximate fitting procedure. While the

estimation method used there was consistent for low-rank models (in the sense that if the data actually arose from a low-rank quadratic model, their approach would converge to the correct parameters as the number of available data grew), the leading eigenvectors of the full second-order term do not estimate the optimal low-rank model when the data arise from a different process. Thus, to provide the fairest comparison to our CGF model fits, we explicitly sought low-rank quadratic forms which were optimal in the sense of regularised least-squares.

A K-dimensional quadratic model takes the form

$$\hat{r}(i) = c + \mathbf{w}^{\mathbf{tf}} \cdot \mathbf{s}(i) + \sum_{k=1}^{K} \lambda_k \big(\mathbf{w}_k^{\mathbf{q}} \cdot \mathbf{s}(i)\big)^2$$

where the vectors $\mathbf{w}_k^{\mathbf{q}}$ ($k = 1 \ldots K$) parametrise the second-order Volterra kernel matrix (V) using K outer products: $V = \sum_{k=1}^{K} \lambda_k \mathbf{w}_k^{\mathbf{q}} \mathbf{w}_k^{\mathbf{q}\mathsf{T}}$. Including the dimension corresponding to the linear term $\mathbf{w}^{\mathbf{tf}}$, this model may also be interpreted as a $(K+1)$-dimensional LN cascade with a second-order polynomial nonlinearity.

A 1-D quadratic model has a similar number of degrees of freedom to the context model (a single quadratic basis component $\mathbf{w}_1^{\mathbf{q}}$ with 720 degrees of freedom in place of the CGF with 324 degrees of freedom). However least-squares fitting of such a model is not straightforward. Thus we first fit a 2-D quadratic model of the form

$$\hat{r}(i) = c + \mathbf{w}^{\mathbf{tf}} \cdot \mathbf{s}(i) + \mathbf{s}(i)^{\mathsf{T}} \mathbf{u} \mathbf{v}^{\mathsf{T}} \mathbf{s}(i)$$

using the same alternating least-squares method as we used to fit the CGF: alternately obtaining least-squares values of $(c, \mathbf{w}^{\mathbf{tf}}, \mathbf{u})$ holding $\mathbf{v}$ fixed, and of $(c, \mathbf{w}^{\mathbf{tf}}, \mathbf{v})$ holding $\mathbf{u}$ fixed. This approach also allowed us to use a regularising prior to improve generalisation — when fitting models for cross-validation we set the prior on $\mathbf{w}^{\mathbf{tf}}$, $\mathbf{u}$ and $\mathbf{v}$ to be the optimal ASD smoothing prior obtained when fitting the STRF model. The least-squares models used to evaluate training fit were unregularised.

Although it exploits a rank 1 decomposition of the quadratic kernel matrix, as long as $\mathbf{u}$ and $\mathbf{v}$ are unconstrained, this model is equivalent to a *two*-dimensional model with λ_k and $\mathbf{w}_k^{\mathbf{q}}$ ($k = 1, 2$) given by the eigenvalues and eigenvectors of $\frac{1}{2}(\mathbf{u}\mathbf{v}^{\mathsf{T}} + \mathbf{v}\mathbf{u}^{\mathsf{T}})$. (The equivalence follows from the observation that $\mathbf{s}^{\mathsf{T}} A \mathbf{s} = 0$ for an antisymmetric matrix A and any vector $\mathbf{s}$, and so only the symmetric part of the product $\mathbf{u}\mathbf{v}^{\mathsf{T}}$ contributes to the model output.) We found the 1-D quadratic model by gradient descent in the mean squared error, constraining $\mathbf{w}_1^{\mathbf{q}}$ to lie within the two-dimensional subspace spanned by the eigenvectors derived from the optimal (regularised or not, as appropriate) 2-D model. Although when using a general structured or natural-sound stimulus, the optimal 1-D quadratic model may not lie within the subspace spanned by the optimal 2-D model, the expected difference vanishes for a independent random stimulus with Gaussian-distributed amplitudes. Numerical experiments suggested that any bias introduced by our two-step estimation process was also small for the independent DRC stimulus.

5 Predicting the STRF for a DRC stimulus from the PRF and CGF (related to Supplementary Fig. S4)

The details of an STRF fit to a nonlinear neural response will depend on details of the stimulus by which the response was evoked. Stimuli with non-trivial statistical structure — such as natural sounds and some artificial stimuli including spectrotemporal ripples — may engage specific nonlinear encoding mechanisms and lead to STRF estimates that substantially misrepresent the neuron's true response properties (Christianson et al., 2008). This general point will also apply to responses with nonlinear context-dependent input-specific gain modulation of the type revealed here, and so an STRF estimated by neglecting contextual effects may differ significantly from the corresponding PRF in ways that reflect the interaction between the context-dependence and the structured statistics of the sound.

However, the DRC stimulus, with its independent and identically distributed (iid) tone pulses, is designed to reduce the effects of such nonlinear distortion (Christianson et al., 2008). In particular, this property means that — provided the dominant combination-dependent nonlinearity in the response is indeed contextual input-specific gain modulation — it is possible to find a closed-form expression for the STRF weights that should be estimated from a DRC stimulus by using the estimated values of the PRF and CGF.

Consider a DRC stimulus with iid pulse energies $s(i, k)$ (where i indexes time and k pulse frequency) that evokes

a measured response $r(i)$ in a neuron whose mean firing rate is accurately described by the quadratic contextual input-specific gain model. Then,

$$r(i) = c + \sum_{j=0}^{J}\sum_{k=1}^{K} w^{\mathbf{tf}}_{j+1,k} s(i-j,k)\left(1 + \sum_{m=0}^{M}\sum_{n=-N}^{N} w^{\boldsymbol{\tau\phi}}_{m+1,n+N+1} s(i-j-m,k+n)\right) + \eta(i)\,, \tag{S3}$$

where c is a firing rate offset, $w^{\mathbf{tf}}_{\cdot,\cdot}$ are the PRF weights, $w^{\boldsymbol{\tau\phi}}_{\cdot,\cdot}$ the CGF weights, and $\eta(i)$ is a noise term with zero mean but otherwise unconstrained distribution. It will be useful to collect the PRF weights into a vector with $L \equiv (J+1)K$ elements, $\mathbf{w^{tf}}$. The subscript notation $\mathbf{w}^{\mathbf{tf}}_{(jk)}$ will then refer to the element of the PRF vector that corresponds to time offset j and frequency bin k in the PRF matrix: that is, $\mathbf{w}^{\mathbf{tf}}_{(jk)} = w^{\mathbf{tf}}_{j+1,k}$. Similarly, we define an L-element stimulus vector $\mathbf{s}(i)$ such that $\mathbf{s}_{(jk)}(i) = s(i-j,k)$; and also an $(L+1)$-element augmented stimulus vector $\tilde{\mathbf{s}}(i) = \begin{bmatrix} 1 \\ \mathbf{s}(i) \end{bmatrix}$

Now consider the estimate of an STRF defined over the same $(J+1)\times K$ region of the stimulus as spanned by the PRF. The STRF model is

$$\hat{r}(i) = c^{\mathrm{STRF}} + \sum_{j=0}^{J}\sum_{k=1}^{K} w^{\mathrm{STRF}}_{j+1,k} s(i-j,k)\,, \tag{S4}$$

where c^{STRF} is model firing rate offset (which might differ from c) and $w^{\mathrm{STRF}}_{\cdot,\cdot}$ are the STRF weights. Again, we define an L-element vector $\mathbf{w^{STRF}}$ with $\mathbf{w}^{\mathbf{STRF}}_{(jk)} = w^{\mathrm{STRF}}_{j+1,k}$, and the $(L+1)$-element vector $\widetilde{\mathbf{w}}^{\mathbf{STRF}} = \begin{bmatrix} c^{\mathrm{STRF}} \\ \mathbf{w^{STRF}} \end{bmatrix}$. Then the least-squares estimate of the STRF parameters is given by the familiar regression form:

$$\widetilde{\mathbf{w}}^{\mathbf{STRF}} = \left\langle \tilde{\mathbf{s}}\tilde{\mathbf{s}}^{\mathsf{T}} \right\rangle^{-1} \langle r\tilde{\mathbf{s}} \rangle\,. \tag{S5}$$

The angle brackets in equation S5 represent averages over time and we drop the explicit time index i from the averaged expressions. We assume that the stimulus used for estimation is long enough for these time-averages to converge to their corresponding expected values (an assumption that also justifies the use of the unregularised maximum-likelihood estimate). The expected value of the estimated STRF for a neuron with a mean firing rate described by the contextual input-specific gain model is then obtained by evaluating the expectations of equation S5 with r set to the value given by equation S3. We perform this evaluation one term at a time.

Consider first the stimulus autocorrelation term $\left\langle \tilde{\mathbf{s}}\tilde{\mathbf{s}}^{\mathsf{T}} \right\rangle^{-1}$. As the tone pulses within the DRC stimulus are iid, the expected value and variance of each stimulus element have constant values, which we write as $\bar{s}$ and σ_s^2 respectively. The expected second moment of each stimulus element is then $\left\langle s(i-j,k)^2 \right\rangle = \bar{s}^2 + \sigma_s^2$; but by independence the cross-moments are just $\langle s(i-j,k)s(i-j',k')\rangle = \bar{s}^2$ when $j \neq j'$ or $k \neq k'$. Assembling these values into matrix form (and writing $\mathbf{1}$ for a vector of L ones and I for the $L \times L$ identity matrix):

$$\left\langle \tilde{\mathbf{s}}\tilde{\mathbf{s}}^{\mathsf{T}} \right\rangle^{-1} = \left\langle \begin{bmatrix} 1 \\ \mathbf{s} \end{bmatrix} \begin{bmatrix} 1 & \mathbf{s}^{\mathsf{T}} \end{bmatrix} \right\rangle^{-1} = \begin{bmatrix} 1 & \langle \mathbf{s} \rangle^{\mathsf{T}} \\ \langle \mathbf{s} \rangle & \left\langle \mathbf{s}\mathbf{s}^{\mathsf{T}} \right\rangle \end{bmatrix}^{-1} = \begin{bmatrix} 1 & \bar{s}\mathbf{1}^{\mathsf{T}} \\ \bar{s}\mathbf{1} & \bar{s}^2\mathbf{1}\mathbf{1}^{\mathsf{T}} + \sigma_s^2 I \end{bmatrix}^{-1} \tag{S6}$$

The inverse follows from the block-matrix identity:

$$\begin{bmatrix} A & B \\ C & D \end{bmatrix}^{-1} = \begin{bmatrix} A^{-1} + A^{-1}BS_{|A}^{-1}CA^{-1} & -A^{-1}BS_{|A}^{-1} \\ -S_{|A}^{-1}CA^{-1} & S_{|A}^{-1} \end{bmatrix} \tag{S7}$$

where $S_{|A} = D - CA^{-1}B$ is the Schur complement of the block A. For the current matrix

$$S_{|A} = \bar{s}^2\mathbf{1}\mathbf{1}^{\mathsf{T}} + \sigma_s^2 I - \bar{s}\mathbf{1}\cdot 1 \cdot \mathbf{1}^{\mathsf{T}}\bar{s} = \sigma_s^2 I \tag{S8}$$

so

$$\left\langle \tilde{\mathbf{s}}\tilde{\mathbf{s}}^{\mathsf{T}} \right\rangle^{-1} = \begin{bmatrix} 1 + \sigma_s^{-2}\bar{s}\mathbf{1}^{\mathsf{T}}\mathbf{1}\bar{s} & -\sigma_s^{-2}\bar{s}\mathbf{1}^{\mathsf{T}} \\ -\sigma_s^{-2}\bar{s}\mathbf{1} & \sigma_s^{-2} I \end{bmatrix} = \sigma_s^{-2} \begin{bmatrix} \sigma_s^2 + L\bar{s}^2 & -\bar{s}\mathbf{1}^{\mathsf{T}} \\ -\bar{s}\mathbf{1} & I \end{bmatrix}\,. \tag{S9}$$

Turning now to the correlation term $\langle r\tilde{\mathbf{s}}\rangle$, we note that as the first element of $\tilde{\mathbf{s}}$ is always 1 we have

$$\langle r\tilde{\mathbf{s}}\rangle_1 = \langle r\rangle = c + \sum_{j=0}^{J}\sum_{k=1}^{K} w^{\mathbf{tf}}_{j+1,k}\bar{s}\left(1 + \sum_{m=0}^{M}\sum_{n=-N}^{N} w^{\boldsymbol{\tau\phi}}_{m+1,n+N+1}\bar{s}\right) + \langle\eta\rangle\,, \tag{S10}$$

where we have used the iid property of the stimulus and the fact that the input gain term does not depend on $s(i-j,k)$. We define $\mathcal{W}_{\text{PRF}} \equiv \sum_{j=0}^{J}\sum_{k=1}^{K} w^{\mathbf{tf}}_{j+1,k}$ and $\mathcal{W}_{\text{CGF}} \equiv \sum_{m=0}^{M}\sum_{n=-N}^{N} w^{\boldsymbol{\tau\phi}}_{m+1,n+N+1}$ and note that the noise has zero mean, giving

$$\langle r\tilde{s}_1\rangle = c + \mathcal{W}_{\text{PRF}}\bar{s} + \mathcal{W}_{\text{PRF}}\mathcal{W}_{\text{CGF}}\bar{s}^2 \equiv \alpha\,, \tag{S11}$$

where the definition of α will be valuable below.

The correlation with the (pq)th element of the stimulus vector is given by

$$\begin{aligned}\left\langle r\mathbf{s}_{(pq)}\right\rangle &= \left\langle \mathbf{s}_{(pq)}\left[c + \sum_{j=0}^{J}\sum_{k=1}^{K} w^{\mathbf{tf}}_{j+1,k}\mathbf{s}_{(jk)}\left(1 + \sum_{m=0}^{M}\sum_{n=-N}^{N} w^{\boldsymbol{\tau\phi}}_{m+1,n+N+1}\mathbf{s}_{(j+m,k+n)}\right)\right]\right\rangle & \text{(S12)}\\ &= c\bar{s} + \sum_{j=0}^{J}\sum_{k=1}^{K} w^{\mathbf{tf}}_{j+1,k}\left\langle\mathbf{s}_{(pq)}\mathbf{s}_{(jk)}\right\rangle + \sum_{j=0}^{J}\sum_{k=1}^{K}\sum_{m=0}^{M}\sum_{n=-N}^{N} w^{\mathbf{tf}}_{j+1,k}w^{\boldsymbol{\tau\phi}}_{m+1,n+N+1}\left\langle\mathbf{s}_{(pq)}\mathbf{s}_{(jk)}\mathbf{s}_{(j+m,k+n)}\right\rangle\,. & \text{(S13)}\end{aligned}$$

Now,

$$\left\langle\mathbf{s}_{(pq)}\mathbf{s}_{(jk)}\right\rangle = \begin{cases}\bar{s}^2 + \sigma_s^2 & \text{if } (pq) = (jk)\\ \bar{s}^2 & \text{otherwise}\,,\end{cases} \tag{S14}$$

and

$$\left\langle\mathbf{s}_{(pq)}\mathbf{s}_{(jk)}\mathbf{s}_{(j+m,k+n)}\right\rangle = \begin{cases}\bar{s}^3 + \sigma_s^2\bar{s} & \text{if } (pq) = (jk) \text{ or } (pq) = (j+m,k+n)\\ \bar{s}^3 & \text{otherwise}\,,\end{cases} \tag{S15}$$

and the case $(jk) = (j+m, k+n)$ does not contribute as the corresponding CGF weight is set to 0. Thus,

$$\begin{aligned}\left\langle r\mathbf{s}_{(pq)}\right\rangle = c\bar{s} + \bar{s}^2\mathcal{W}_{\text{PRF}} + \sigma_s^2\mathbf{w}^{\mathbf{tf}}_{(pq)} + \bar{s}^3\mathcal{W}_{\text{PRF}}\mathcal{W}_{\text{CGF}} + \sigma_s^2\bar{s}\mathcal{W}_{\text{CGF}}\mathbf{w}^{\mathbf{tf}}_{(pq)}\\ + \sigma_s^2\bar{s}\sum_{j=0}^{p-1}\sum_{k=\max(1,q-N)}^{\min(K,q+N)} w^{\mathbf{tf}}_{j+1,k}w^{\boldsymbol{\tau\phi}}_{p-j+1,q-k+N+1}\,.\end{aligned} \tag{S16}$$

Now recall the definition of α from equation S11, and further define $\beta \equiv (1 + \bar{s}\mathcal{W}_{\text{CGF}})$ as well as $\mathbf{w}^{\mathbf{conv}}_{(pq)} \equiv \sum_{jk} w^{\mathbf{tf}}_{j+1,k}w^{\boldsymbol{\tau\phi}}_{p-j+1,q-k+N+1}$ with limits as in equation S16, so that $\mathbf{w}^{\mathbf{conv}}$ is the vector representing the $(J+1)\times K$ region of the 2D convolution between the CGF and PRF that is central in frequency and causal in time. We can then write:

$$\left\langle r\mathbf{s}_{(pq)}\right\rangle = \bar{s}\alpha + \sigma_s^2\beta\mathbf{w}^{\mathbf{tf}}_{(pq)} + \sigma_s^2\bar{s}\mathbf{w}^{\mathbf{conv}}_{(pq)}\,. \tag{S17}$$

Finally, combining equations S9, S11 and S17, we have

$$\widetilde{\mathbf{w}}^{\mathbf{STRF}} = \left\langle\tilde{\mathbf{s}}\tilde{\mathbf{s}}^{\mathsf{T}}\right\rangle^{-1}\langle r\tilde{\mathbf{s}}\rangle = \sigma_s^{-2}\begin{bmatrix}\sigma_s^2 + L\bar{s}^2 & -\bar{s}\mathbf{1}^{\mathsf{T}}\\ -\bar{s}\mathbf{1} & I\end{bmatrix}\begin{bmatrix}\alpha\\ \bar{s}\alpha\mathbf{1} + \sigma_s^2\beta\mathbf{w}^{\mathbf{tf}} + \sigma_s^2\bar{s}\mathbf{w}^{\mathbf{conv}}\end{bmatrix}\,, \tag{S18}$$

which, with some simplification and setting $\mathcal{W}_{\text{conv}} \equiv \sum_{(pq)}\mathbf{w}^{\mathbf{conv}}_{(pq)}$, yields:

$$\widetilde{\mathbf{w}}^{\mathbf{STRF}} = \begin{bmatrix}c - \bar{s}^2\mathcal{W}_{\text{conv}}\\ \beta\mathbf{w}^{\mathbf{tf}} + \bar{s}\mathbf{w}^{\mathbf{conv}}\end{bmatrix}\,. \tag{S19}$$

Thus, the expected weights of the STRF correspond to the weights of the PRF scaled by the factor β and modified by the convolutional factor $\bar{s}\mathbf{w}^{\mathbf{conv}}$.

The accuracy of this prediction is shown in Supplementary Fig. S4.

Supplementary References

Ahrens, M. B., Paninski, L., and Sahani, M. (2008). Inferring input nonlinearities in neural encoding models. *Network*, 19(1):35–67.

Anderson, L. A., Christianson, G. B., and Linden, J. F. (2009). Mouse auditory cortex differs from visual and somatosensory cortices in the laminar distribution of cytochrome oxidase and acetylcholinesterase. *Brain Res.*, 1252:130–142.

Anderson, L. A. and Linden, J. F. (2011). Physiological differences between histologically defined subdivisions in the mouse auditory thalamus. *Hear. Res.*, 274(1-2):48–60.

Christianson, G. B., Sahani, M., and Linden, J. F. (2008). The consequences of response nonlinearities for interpretation of spectrotemporal receptive fields. *J. Neurosci.*, 28(2):446–455.

Duda, R. O. and Hart, P. E. (1973). *Pattern Classification and Scene Analysis.* Oxford University Press.

Lewicki, M. S. (1998). A review of methods for spike sorting: the detection and classification of neural action potentials. *Network*, 9(4):53–78.

Linden, J. F., Liu, R. C., Sahani, M., Schreiner, C. E., and Merzenich, M. M. (2003). Spectrotemporal structure of receptive fields in areas AI and AAF of mouse auditory cortex. *J. Neurophysiol.*, 90(4):2660–2675.

Park, I., Archer, E., Priebe, N. J., and Pillow, J. W. (2013). Spectral methods for neural characterization using generalized quadratic models. *Advances in Neural Information Processing Systems*, 26:2454–2462.

Sahani, M. (1999). *Latent variable models for neural data analysis.* PhD thesis, California Institue of Technology.

Sahani, M. and Linden, J. F. (2003a). Evidence optimization techniques for estimating stimulus-response functions. In Becker, S., Thrun, S., and Obermayer, K., editors, *Advances in neural information processing systems*, pages 301–308. MIT Press, Cambridge, MA.

Sahani, M. and Linden, J. F. (2003b). How linear are auditory cortical responses? In Becker, S., Thrun, S., and Obermayer, K., editors, *Advances in neural information processing systems*, pages 109–116. MIT Press, Cambridge, MA.

Smith, P. H. and Populin, L. C. (2001). Fundamental differences between the thalamocortical recipient layers of the cat auditory and visual cortices. *J. Comp. Neurol.*, 436(4):508–519.
